# Genetic Structure and Selection Signals for Extreme Environment Adaptation in *Lop Sheep* of Xinjiang

**DOI:** 10.3390/biology14040337

**Published:** 2025-03-25

**Authors:** Chenchen Yang, Jieru Wang, Lanshu Bi, Di Fang, Xin Xiang, Abliz Khamili, Waili Kurban, Chunmei Han, Qinghua Gao

**Affiliations:** 1 College of Animal Science and Technology, Tarim University, Alar 843300, China; 10757223066@stumail.taru.edu.cn (C.Y.); difang@taru.edu.cn (D.F.); 10757223056@stumail.taru.edu.cn (X.X.); 2 College of Life Science and Technology, Tarim University, Alar 843300, China; jieru@stumail.taru.edu.cn; 3 Xinjiang Bazhou Animal Husbandry Work Station, Bazhou 841000, China; 18909968577@163.com; 4 Key Laboratory of Livestock and Forage Resources Utilization Around Tarim, Ministry of Agriculture and Rural Areas, Alar 843300, China; 5 Bureau of Agriculture and Rural Development, Ruoqiang County, Bazhou 841000, China; 13779331376@163.com; 6 Agricultural Development Centre, Utamu Township, Ruoqiang County, Bazhou 841000, China; 15352528002@163.com

**Keywords:** *Lop sheep*, genetic diversity, population structure, selection signal

## Abstract

Objective: *Lop sheep* species exhibit remarkable adaptability to desert pastures and extreme arid climates, demonstrating tolerance to rough feeding and high resistance to stress. However, little is known about the population genetic diversity of *Lop sheep* and the genetic mechanisms underlying their adaptability to extreme environments. Methods: Blood samples were collected from a total of 110 individuals comprising 80 Ruoqiang *Lop sheep* and 30 Yuli *Lop sheep*. A total of 110 *Lop sheep* were subjected to whole genome resequencing to analyze genetic diversity, population structure, and signatures of selection in both regions. Results: The genetic diversity of the *Lop sheep* population is substantial, and the degree of inbreeding is low. In comparison to the *Lop sheep* in Yuli County, the genetic diversity and linkage disequilibrium analysis results for the *Lop sheep* population in Ruoqiang County are slightly lower. Population structure analysis indicates that Ruoqiang and Yuli *Lop sheep* have differentiated into two independent groups. Using Yuli *Lop sheep* as the reference group, an analysis of the extreme environmental adaptability selection signal of *Lop sheep* was conducted. The FST and π ratio under the 1% threshold identified 1686 and 863 candidate genes, respectively, with their intersection yielding a total of 122 candidate genes. Functional annotation revealed that these genes are associated with various traits, including immune response (*SLC12A2*, *FOXP1*, *PANX1*, *DYNLRB2*, *RAP1B*, and *SEMA4D*), heat and cold resistance (*DNAJC13*, *PLCB1*, *HIKESHI*, and *PITPNC1*), desert adaptation (*F13A1*, *PANX1*, *ST6GAL1*, *STXBP3*, *ACTN4*, and *ATP6V1A*), and reproductive performance (*RAP1B*, *RAB6A*, *PLCB1*, and *METTL15*). Conclusions: These research findings provide a theoretical foundation for understanding the survival and reproductive characteristics of *Lop sheep* in extreme environments, and they hold practical value for the conservation and utilization of *Lop sheep* genetic resources, as well as for genetic improvement efforts.

## Simple Summary

*Lop sheep* are distributed in the Lop Nur area of the Taklimakan Desert in Xinjiang. This unique local breed is exceptionally well suited for desert pastures, thriving in extremely arid climates, exhibiting resilience to rough feeding, and demonstrating a high tolerance for stress. However, the genetic mechanism of *Lop sheep* adaptation in extreme desert environments has not been studied. In this study, we used whole genome resequencing data to analyze the genetic diversity, population genetic structure, and selection signals of *Lop sheep.* The results showed that the Ruoqiang and Yuli *Lop sheep* produced genetic differentiation; through the analysis of selection signals, we screened out candidate genes related to the adaptability of *Lop sheep* in extreme desert environments. This study provides a theoretical basis for the conservation and subsequent breeding of *Lop sheep* by exploring the genetic characteristics of the *Lop sheep* population.

## 1. Introduction

**Figure 1:**
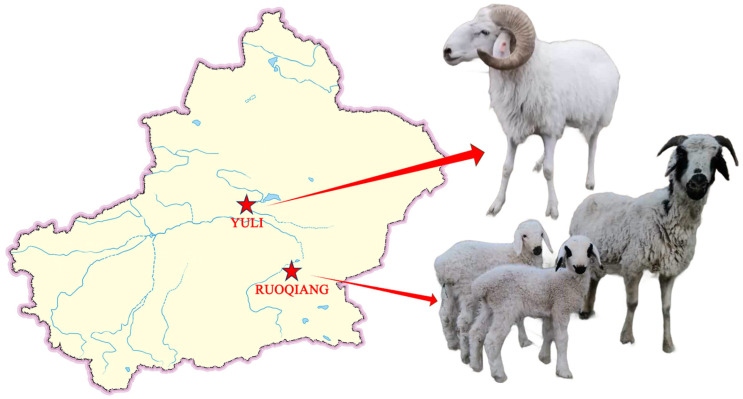
Geographical distribution of *Lop sheep* in Yuli and Ruoqiang counties, Xinjiang, China.

*Lop sheep* are distributed in the Lop Nur area of the Taklamakan Desert in Xinjiang. This unique local breed is exceptionally well suited for desert pastures, thriving in extremely arid climates, exhibiting resilience to rough feeding, and demonstrating a high tolerance for stress [1,2]. Currently, there are two populations of *Lop sheep* located in Ruoqiang County and Yuli County (Figure 1). Ruoqiang County, situated on the southeastern edge of the Taklimakan Desert, experiences perennial drought and a hot climate [3]. Local grazing primarily occurs on desert and semi-desert pastures, with forage types mainly including tamarisk, poplar leaves, reed grass, licorice, and camel thorns [4]. In this area, *Lop sheep* are predominantly grazed using traditional methods. In contrast, Yuli County features two major alluvial plains, where the climate is milder and more humid than that of Ruoqiang County. Pasture resources in Yuli County account for 14.71% of its total land area, with alfalfa and other green fodder being relatively abundant [5]. Here, Lop sheep are primarily fed in sheds alongside traditional grazing practices. Due to regional differences, the genetic characteristics and phenotypes of the *Lop sheep* populations in these two locations vary. Survey results indicate that the lambing rate of *Lop sheep* in Ruoqiang County is higher than that in Yuli County, and the meat quality and flavor are superior. Ruoqiang *Lop sheep* exhibit strong adaptability to the extreme natural environment of Lop Nur, characterized by drought, sandstorms, and high summer temperatures [6,7].

Lop Nur’s remote desert location limits genetic exchange between *Lop sheep* and other breeds, thereby preserving the local breed’s population structure while also subjecting it to greater natural selection pressures. This unique environment has resulted in a distinctive set of physiological traits, including disease resistance, heat tolerance, and drought resilience [8,9]. However, the rapid development of the animal husbandry economy has introduced foreign breeds that adversely affect *Lop sheep* through indiscriminate mating with other local breeds. This practice has led to alterations in the genetic structure of the *Lop sheep* population and a swift decline in its germplasm, making breed protection an urgent priority [10]. A research team previously employed microsatellite markers and mtDNA D-loop sequences to investigate the genetic diversity of the *Lop sheep* population in the Yuli area, revealing that this population possesses significant genetic diversity and high breeding value [1]. The current study utilizes whole-genome resequencing technology to reassess the genetic diversity and population structure characteristics of *Lop sheep* across two regions, providing a comprehensive exploration of their genetic traits. The findings of this research will serve as a theoretical foundation for the protection and future selective breeding of Xinjiang *Lop sheep*.

## 2. Materials and Methods

### 2.1. Animal Care

All animal experiments were conducted in accordance with the “Regulations and Guidelines for the Management of Experimental Animals” established by the Ministry of Science and Technology (Beijing, China, 2020 revision). This study was approved by the Institutional Animal Care and Use Committee of Tarim University, Xinjiang, China (protocol code SYXK2020-009; approval date: 1 January 2020).

### 2.2. Sample Collection and Sequencing

The way we collected the different samples was based on the genealogical information of the Ruoqiang and Yuli *Lop sheep* conservation populations, selection of purebred *Lop sheep* that have not been crossbred for more than three generations, where jugular vein blood samples were collected from 80 and 30 *Lop sheep* from Ruoqiang and Yuli counties, respectively, in Bayin’guoleng Autonomous Prefecture, Xinjiang, China (Supplementary Table S7). No experimental animals were killed during collection. Whole genome (10X) sequencing was performed by a BGI T7 sequencer (Beijing, China) according to the manufacturer’s recommendations. The datasets analyzed during the current study are available, at the China National Center for Bioinformation/Beijing Institute of Genomics, Chinese Academy of Sciences repository (https://ngdc.cncb.ac.cn/gvm/, accessed on 12 December 2024; GVM: GVM000916). In addition, we downloaded 11 Asiatic mouflon (PRJNA624020) at the National Center for Biotechnology Information (NCBI) (https://www.ncbi.nlm.nih.gov/, accessed on 12 December 2024).

### 2.3. Genotyping and Quality Control

Fastp v0.23.2 [11] was employed to assess per-base sequence quality using default parameters. Following quality control, the clean reads were aligned against the sheep reference genome (https://www.ncbi.nlm.nih.gov/datasets/genome/GCF_016772045.1/, accessed on 1 June 2024) utilizing bwa-mem2 v2.2.1 [12]. The command used was as follows: bwa-mem2 mem -R “@RG\tID: $line\tPL: ILLUMINA\tSM: $line” -t 12 > sam. Subsequently, using SAMtools v.1.9 [13], the mapped reads were converted to BAM files, which were sorted using default parameters with commands such as samtools view -bS A.sam > A.sort.bam. GATK v4.1.4.1 [14] was then applied with the command ‘gatk MarkDuplicates -I A.sort.bam -M A.metrics -O A.sort.MarkDup.bam’. SNPs and Indels were called from the BAM files using the GATK HaplotypeCaller module, adhering to GATK best-practice recommendations. The recommended command for this process was ‘gatk HaplotypeCaller -R GCF_016772045.1.fna -I A.sort.MarkDup.bam -O A.g.vcf.gz’. Raw GVCFs, generated from individual samples, were merged using CombineGVCFs and subsequently genotyped with GenotypeGVCFs. We then extracted and filtered SNPs using the GATK SelectVariants module, employing the command ‘gatk SelectVariants -R GCF_016772045.1.fna -V output.vcf.gz -select-type-to-include SNP -O raw.snps.genotype.vcf’. To mitigate potential false-positive calls, we implemented GATK’s “Variant-Filtering” for the selected SNPs, applying the best practice parameters: ‘QD 60.0 || SOR > 3.0 || MQRankSum < −12.5 || ReadPosRankSum < −8.0’. The obtained SNP files require further filtering using VCFtools v0.1.16 [15] to eliminate indels in markers, applying the parameters (--minDP 2 --min-alleles 2 --max-alleles 2). The raw genotype data were merged utilizing PLINK v1.90 (https://www.cog-genomics.org/plink/, accessed on 10 July 2024), excluding SNPs located on sex chromosomes and those with unknown chromosomal locations. Subsequently, PLINK was employed to conduct quality control (QC) on these SNPs, filtering SNP sites and individuals based on the parameters (--geno 0.1 -hwe 0.0000001 -maf 0.05 -mind 0.15). After quality screening, all identified SNPs were further annotated using ANNOVAR (https://annovar.openbioinformatics.org/, accessed on 11 July 2024) based on the gene annotations of the sheep reference genome. This annotation included the locations of SNPs in various genic and intergenic regions, as well as the classification of synonymous and nonsynonymous SNPs within exonic regions.

### 2.4. Genetic Diversity and Population Structure

PLINK v1.90 software was utilized to calculate observed heterozygosity (Ho), expected heterozygosity (He), and nucleotide polymorphism (π). PopLDdecay v3.40 (https://github.com/BGI-shenzhen/PopLDdecay.git, accessed on 25 December 2024) was employed to compute the squared correlation (r^2^) between loci, facilitating linkage disequilibrium (LD) analysis. PLINK parameters such as (--indep -pairwise 25 5 0.2) were applied to prune and eliminate highly linked pairs of SNPs. Execute PLINK commands, including (plink –vcf A.vcf --pca), to conduct principal component analysis (PCA). VCF2Dis software (https://github.com/BGI-shenzhen/VCF2Dis, accessed on 26 December 2024) was used to calculate the P-distance matrix, and subsequently, the ATGC program (http://www.atgc-montpellier.fr/, accessed on 26 December 2024) was employed to construct phylogenetic relationships, specifically generating a phylogenetic tree (NJ-tree). This tree was visualized and analyzed using the iTOL tool (https://itol.embl.de/, accessed on 26 December 2024). Lastly, ADMIXTURE v1.30 (https://dalexander.github.io/admixture/binaries/admixture_linux-1.3.0.tar.gz, accessed on 27 December 2024) was used to perform ancestry component analysis, setting the K values 2, 3, and 5, with results visualized through TBtools (https://github.com/CJ-Chen/TBtools, accessed on 27 December 2024).

### 2.5. Selection of Signal Analyses

To accurately elucidate the adaptability mechanisms of Xinjiang Lop sheep in extreme climatic environments, we employed two selection signal methods: FST and π ratio. By intersecting the results of FST and π ratio, we identified potential candidate gene regions that are indicative of strong natural selection in Lop sheep under extreme climatic conditions.

The genetic differentiation index (FST) is a widely used method for analyzing selection signals. The VCTFTTOLS v0.1.16 software facilitates the assessment of genetic differentiation between two populations. A higher differentiation index indicates a more pronounced difference between the populations [16]. The formula is as follows:

$$FST=\frac{MSP-MSG}{MSP+(nc-1)MSG}$$

*MSG* represents the mean squared error within populations, *MSP* represents the mean squared error between populations, and *nc* is the average sample size corrected for the entire population.

π represents a parameter for nucleotide polymorphism. The π ratio is a method used to analyze selection signals by examining the differences in π between wild and domesticated populations. This approach was introduced to quantify the loss of population polymorphism in domesticated populations compared to their wild counterparts [17]. The formula is as follows:

$$\pi \;ratio=\frac{\pi (wild)}{\pi (domesticcated)}$$

π ratio denotes the ratio of nucleotide diversity between the wild and domesticated populations, π(wild) is the nucleotide diversity of the wild population, and π(domesticated) is the nucleotide diversity of the domesticated population. Positive Log2(π ratio) values indicate that the nucleotide polymorphisms in the window became lower and were selected for domestication; the larger the value of Log2(π ratio), the greater the value of Log2(π ratio), and the higher the value of Log2(π ratio), the greater the value of Log2(π ratio). The larger the Log2(π ratio) value, the higher the intensity of selection by domestication.

VCFTOOLS v0.1.16 software were used to compute FST and π ratio under autosomal conditions, employing a sliding window approach with a 100 kb window size and a 50 kb step size. Subsequently, a top 1% threshold was applied to identify significant selection signals and filter for intersecting genes [18].

### 2.6. Gene Functional Enrichment Analysis

Based on the candidate genes, the intersection genes of FST and π ratio were visualized using VENNY (https://bioinfogp.cnb.csic.es, accessed on 10 November 2024). Annotation was conducted using the GCF_016772045.1_ARS-UI_Ramb_v2.0_genomic.gtf file. Pathway enrichment analysis was performed utilizing Gene Ontology (GO) and the Kyoto Encyclopedia of Genes and Genomes (KEGG) [19] to investigate the biological enrichment of genes under selective pressure. Additionally, gene networking analysis was conducted on overlapping candidate genes obtained through pairwise comparisons. GO terms and KEGG pathways were considered significantly enriched when the *p*-value was less than 0.05. The drawing was completed using R v4.4.3 software(https://www.r-project.org/, accessed on 1 May 2024).

## 3. Results

### 3.1. Sequencing and Identification of SNPs

**Figure 2:**
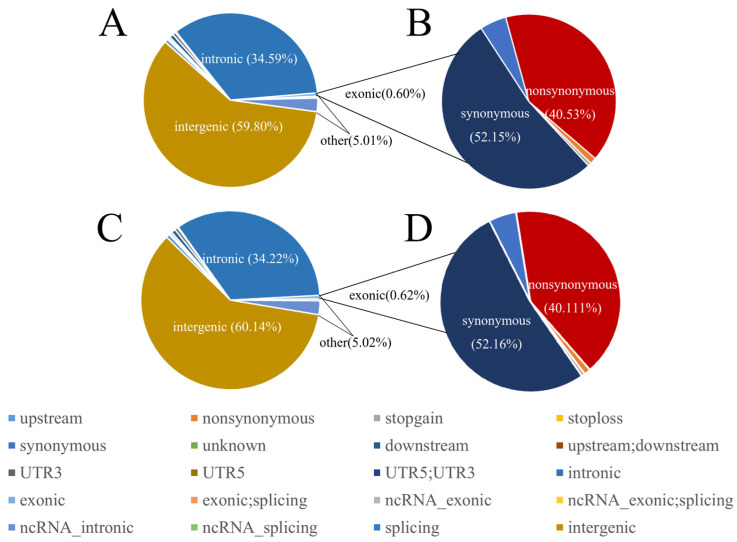
Statistics regarding the whole genome SNP variant types of RuoQiang and YuLi *Lop sheep* using ANNOVAR. (**A**,**B**) and (**C**,**D**) are Ruoqiang and Yuli *Lop sheep*. Plot of the total variant annotation and coding consequence variant annotation.

Whole-genome sequencing of 110 blood samples from *Lop sheep* generated approximately 36 GB of genomic data per sample, with an average sequencing depth of about 10×. A total of 36,416,239 bi-allelic SNPs were detected, with the number of SNPs in Ruoqiang exceeding that in Yuli *Lop sheep.* The polymorphic loci were annotated using ANNOVAR ($Date: 2020-06-07 23:56:37 -0400 (Sun, 7 Jun 2020)) software, revealing that the majority of SNPs in Yuli *Lop sheep* were located in intergenic regions (59.80%) or intronic regions (34.59%), while exons comprised only 0.60% of the total SNPs. Among these, 45,176 (52.15%) were synonymous and 35,613 (40.53%) were non-synonymous. In Ruoqiang *Lop sheep*, the same total of 36,416,239 bi-allelic SNPs was detected, with the majority also found in intergenic regions (60.14%) or intronic regions (34.22%), and exons accounted for 0.62% of the total SNPs. Of these, 44,982 (52.15%) were synonymous SNPs and 34,575 (40.53%) were non-synonymous SNPs (Figure 2A–D, Supplementary Table S1).

### 3.2. Analysis of Genetic Diversity and Linkage Disequilibrium in Lop Sheep Populations

**Figure 3:**
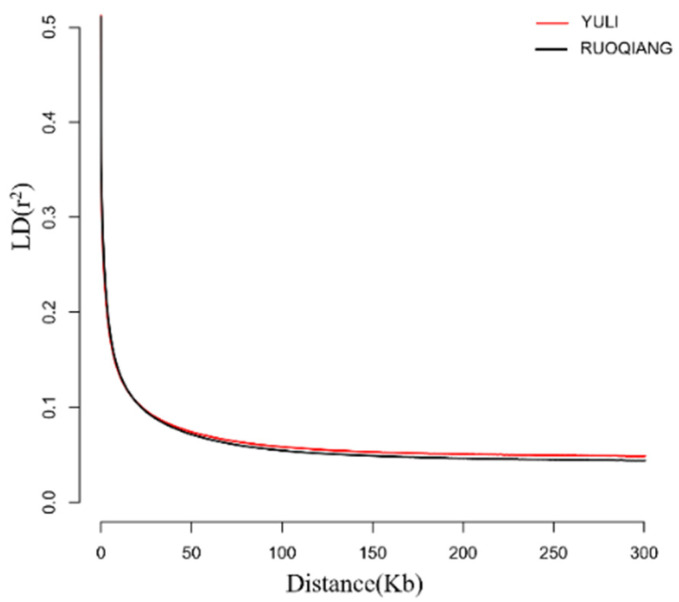
Results of chain imbalance attenuation map of Yuli and Ruoqiang *Lop sheep*.

Through a comparative analysis of genetic diversity indicators, the observed heterozygosity (Ho) of the *Lop sheep* population was found to be 0.2938, while the expected heterozygosity (He) was 0.3011, the nucleotide diversity (π) was 3.36 × 10^−3^, and the inbreeding coefficient (Froh) was 0.0172. These results indicate that the genetic diversity of the *Lop sheep* population is relatively high. In comparison, the heterozygosity value of the Ruoqiang *Lop sheep* is slightly lower than that of the Yuli *Lop sheep*, and the inbreeding coefficient of the Ruoqiang *Lop sheep* is higher than that of the Yuli *Lop sheep* (Table 1). Additionally, the linkage disequilibrium (LD) results suggest that the degree of domestication of the Ruoqiang *Lop sheep* population is lower than that of the Yuli *Lop sheep* population (Figure 3).

### 3.3. Comparison of Population Structure Characteristics of Lop Sheep in Two Regions

**Figure 4:**
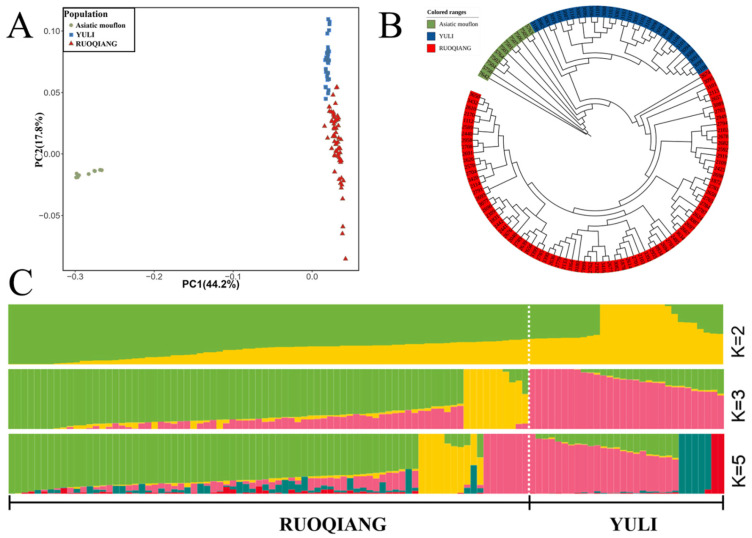
Illustrates a comparison of the population structure characteristics of Yuli and Ruoqiang *Lop sheep*. (**A**) Principal component analysis of Yuli and Ruoqiang *Lop sheep*, with the *X*-axis representing PC1 and the *Y*-axis representing PC2. (**B**) Neighbor-joining (NJ) tree depicting the relationships between the two groups of *Lop sheep* from Yuli and Ruoqiang. (**C**) ADMIXTURE results for Yuli and Ruoqiang *Lop sheep* are presented, showing population sizes k of 2, 3, and 5, with different colors indicating distinct ancestral components.

Principal component analysis (PCA), neighbor-joining (NJ) tree, and ADMIXTURE methods were employed to analyze the genetic structure and relationships of *Lop sheep* in the two regions. The results indicated that when using Asian wild sheep as the reference group, Ruoqiang and Yuli *Lop sheep* were classified into two distinct groups (Figure 4A,B, Supplementary Tables S2 and S3). The ADMIXTURE analysis revealed that at K = 2, the Yuli and Ruoqiang *Lop sheep* populations exhibited clear genetic differentiation. As the K value increased, these two populations displayed the highest proportions of ancestral components corresponding to their respective geographical areas (Figure 4C, Supplementary Table S4).

### 3.4. Selective Signal Analysis

**Figure 5:**
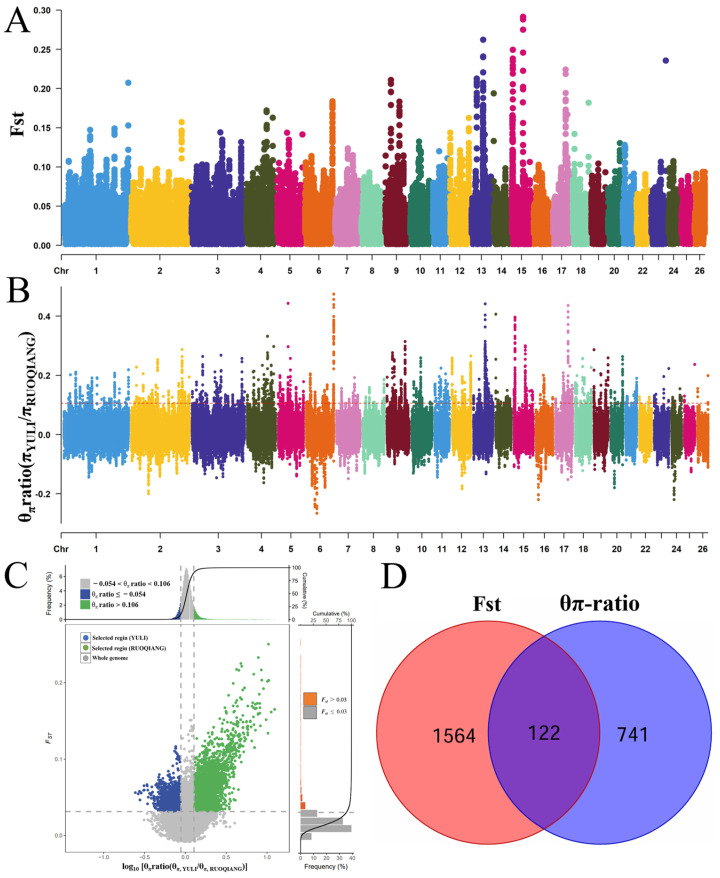
Presents an analysis of population selection signals in the *Lop sheep* populations of Yuli and Ruoqiang. (**A**) Displays the FST Manhattan plot for both Yuli and Ruoqiang. (**B**) Illustrates the Manhattan plot of the π ratio for these populations. (**C**) Provides a selective elimination analysis based on the FST and π ratio. (**D**) Features a Venn diagram depicting the intersection of the results from the FST and π ratio analyses.

**Figure 6:**
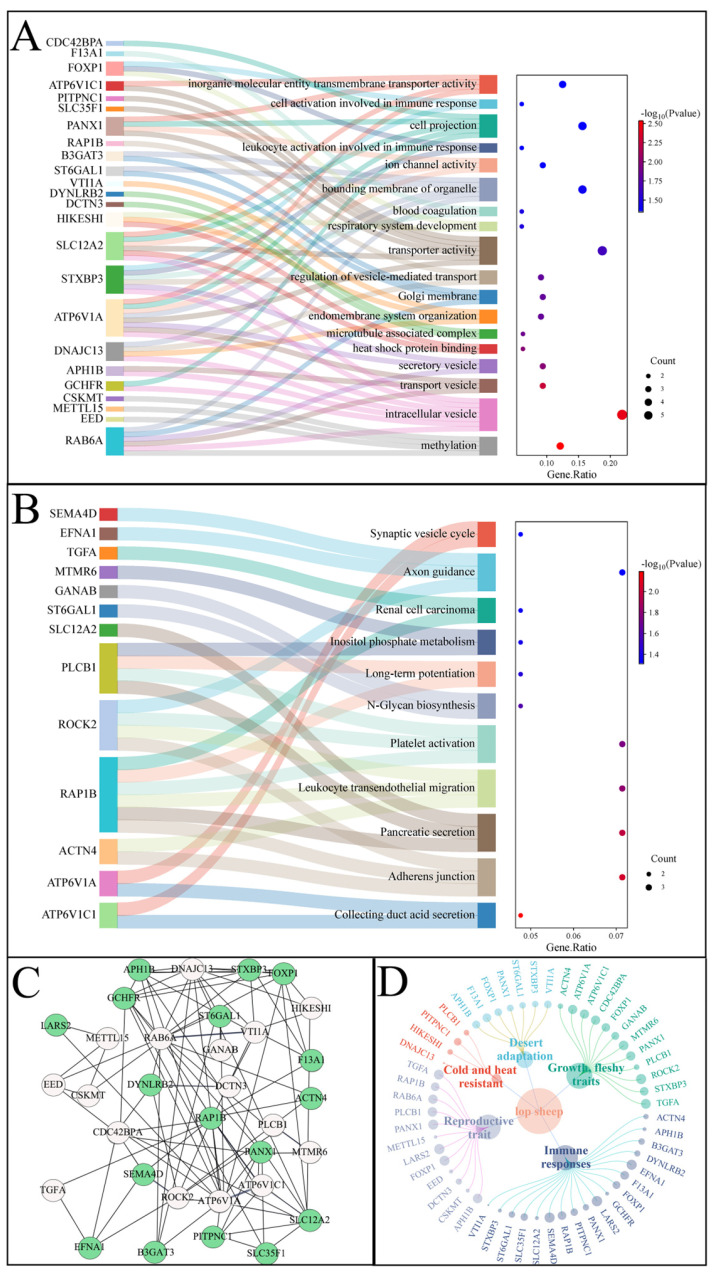
GO and KEGG pathway enrichment analysis shows significant (*p* < 0.05) terms, pathways, and associated genes in Ruoqiang vs. Yuli *Lop sheep* comparison. (**A**) The Sankey dot plot of the Top 18 GO terms. (**B**) The Sankey dot plot of the 11 significant KEGG pathways. The size of circles for each pathway represents counts of associated genes. The color of the circles indicates the *p*-value. (**C**) Gene networking analysis of 33 overlapping genes (*p* < 0.05). Green is identified as a candidate gene for the immune response in *Lop sheep.* (**D**) The network aggregation diagram illustrates 33 candidate genes that are annotated to five major traits (*p* < 0.05).

According to the field investigation of the ecological environment, the feeding mode, and the population size of the Ruoqiang and Yuli *Lop sheep* populations, the Ruoqiang *Lop sheep* has a strong adaptive ability to extreme environments. Secondly, according to the analysis of the genetic structure of the Ruoqiang and Yuli *Lop sheep* populations, it can be concluded that there are genetic differences between the Ruoqiang and Yuli *Lop sheep* populations at present. Consequently, this study utilized Yuli *Lop sheep* as the reference group and conducted selection signal analysis using FST (0.068) and π ratio (0.106). The top 1% were designated as candidate regions, resulting in a total of 1686 and 863 candidate genes identified after screening (Figure 5A–C). Following the elimination of duplicate and invalid genes, the genes identified through FST and π ratio were intersected, yielding a total of 122 candidate genes (Figure 5D, Supplementary Table S5). Subsequent GO and KEGG enrichment analyses revealed that 51 GO terms and 11 KEGG pathways were significantly enriched (*p* < 0.05) (Figure 6A,B, Supplementary Table S6).

Among the identified genes, those most significantly involved in immune response include *APH1B*, *B3GAT3*, *ACTN4*, *DYNLRB2*, *EFNA1*, *F13A1*, *LARS2*, *SEMA4D*, *SLC12A2*, *SLC35F1*, *ST6GAL1*, *STXBP3*, *GCHFR*, *PITPNC1*, *PANX1*, and *RAP1B* (Figure 6C). The genes *DNAJC13*, *PLCB1*, *HIKESHI*, and *PITPNC1* are associated with heat and cold resistance. Additionally, *F13A1*, *PANX1*, *ST6GAL1*, *STXBP3*, *VTI1A*, *APH1B*, and *FOXP1* are linked to the desert adaptability of Lop sheep. The remaining genes are related to growth and meat quality traits, including *ATP6V1A*, *ATP6V1C1*, *GANAB*, *CDC42BPA*, *MTMR6*, *PANX1*, *ROCK2*, *TGFA*, *ACTN4*, *PLCB1*, *STXBP3*, and *FOXP1*, as well as reproductive traits, which encompass *DCTN3*, *EED*, *CSKMT*, *METTL15*, *RAB6A*, *RAP1B*, *APH1B*, *PLCB1*, *PANX1*, *FOXP1*, *LARS2*, and *TGFA* (Figure 6C,D, Supplementary Table S6).

## 4. Discussion

From a whole-genome perspective, the results of He, Ho, π, and Froh indicate that the genetic diversity of the *Lop sheep* population is relatively high. This result aligns with the previous findings of our research group, which utilized microsatellite markers and mtDNA D-loop sequences to investigate the genetic diversity of the Lop sheep population. The genetic diversity of the Yuli *Lop sheep* population in Xinjiang is greater than that of the Ruoqiang *Lop sheep*, and the inbreeding coefficient of the Ruoqiang *Lop sheep* is higher than that of the Yuli Lop sheep. Yuli County is the largest production area for *Lop sheep*, with a population of approximately 65,000. The region features extensive plains and well-developed transportation infrastructure. With the opening of the Silk Road, exchanges among various Yuli *Lop sheep* populations increased significantly, along with hybridization with other sheep breeds, so the inbreeding coefficient within the Yuli *Lop sheep* population is low, indicating that the genetic diversity of Yuli *Lop sheep* is slightly higher; Ruoqiang County serves as another production area for *Lop sheep*, with an estimated population of approximately 11,000. However, the region’s mountainous terrain and the dispersed nature of human settlements pose challenges to its development. The Ruoqiang *Lop sheep* are primarily utilized for traditional grazing, spending most of the year in mountainous areas. This lifestyle restricts interactions between different populations of Ruoqiang *Lop sheep*. Additionally, limited exchanges with other sheep breeds contribute to a lower genetic diversity compared to the Yuli *Lop sheep*. This results in lower genetic diversity compared to Yuli *Lop sheep* and indicates a significant level of inbreeding within the Ruoqiang *Lop sheep* population. The LD results showed that the rate of LD decline was basically the same in Ruoqiang and Yuli *Lop sheep*. The faster the LD decline, the higher the genetic diversity; inbreeding coefficients, if high, were relatively lower. The Ruoqiang *Lop sheep* live in a more hostile natural environment with limited resources and high environmental pressure, which may have led to a stronger bottleneck effect and natural selection, thus increasing the inbreeding rate (Froh) but at the same time decreasing the linkage disequilibrium (LD) in some genomic regions. On the contrary, Yuli *Lop sheep* live in a relatively mild environment with more abundant resources and less influence of natural selection and bottleneck effect; thus, they have lower inbreeding rates and higher levels of linkage disequilibrium. In this study, the higher LD level of Yuli *Lop sheep* may be related to its relatively stable environment and less selective pressure, which made certain regions of its genome maintain a high LD, thus maintaining genetic diversity to a certain extent. On the contrary, Ruoqiang *Lop sheep* may have experienced a decrease in LD in some regions of their genome due to experiencing more intense selective pressure and bottleneck effects, which may be a reason for their reduced genetic diversity.

Population structure analysis indicates that the Ruoqiang and Yuli *Lop sheep* populations share a common ancestor, while significant genetic differentiation exists between the Yuli and Ruoqiang *Lop sheep* populations. According to the literature, the origin and evolution of the *Lop sheep* are closely linked to the Lop people. In the mid-to-late 18th century, as Lop Nur gradually dried up, some *Lop sheep* accompanied the Lop people and migrated to the present-day Milan area of Ruoqiang County, while others moved north to Yuli County, specifically to Kalquke Village [1]. Since Ruoqiang and Yuli counties are situated in distinct geographical environments, *Lop sheep* encounter varying environmental selection pressures. Ruoqiang County is located on the southeastern edge of the Taklimakan Desert, characterized by perennial drought and a hot climate. Grazing in this area primarily occurs on desert and semi-desert grasslands. In contrast, Yuli County features two major alluvial plains, and its climatic conditions are milder and wetter than those of Ruoqiang County; the *Lop sheep* in the two regions have undergone adaptations to their local climate and environment. Due to their prolonged existence in expansive desert areas, communication between the Lop sheep populations in these regions has been limited, leading to phenotypic and genetic differences.

Through GO and KEGG enrichment analyses, a total of 27 immune-related gene pathways were identified, including the *SLC12A2*, *FOXP1*, *PANX1*, *DYNLRB2*, *RAP1B*, and *SEMA4D* genes. The *SLC12A2* pathway and chloride flux represent a ‘reversible switch’ controlling the antiv pro-inflammatory response of a phagocyte after apoptotic cell uptake [20]; the *FOXP1* gene regulates the development of follicular helper and regulatory T-cells, and regulatory T (Treg) cells play central roles in maintaining immune homeostasis and self-tolerance [21]; selective inhibition of Panx1 channels may not only limit acute inflammatory responses, and pannexins play important roles in activation of both post-injury inflammatory response and the subsequent process of tissue regeneration [22]; the *DYNLRB2* gene was found to be associated with innate immunity in C57BL/6 mice [22]; the *RAP1B* gene is crucial for early B cell development, MZ B cell homeostasis, and T-dependent humoral immunity [23]; *SEMA4D* is involved in humoral and cell-based immune responses and is important in adaptive and innate immune responses to hypersensitivity reactions [24]; the synergistic interaction of these genes enables Lop sheep to better adapt to extreme drought conditions, thereby enhancing their survival and disease resistance.

Heat and cold tolerance are significant factors influencing animal production, health, and fertility. In the Lop region, where summer temperatures exceed 40 °C and winter lows drop below −20 °C, the considerable temperature fluctuations between day and night impose stringent demands on the heat and cold resistance of Lop sheep. Genetic screening revealed several relevant genes, including *DNAJC13*, *PLCB1*, *HIKESHI*, and *PITPNC1*. The *DNAJC13* gene is linked to heat tolerance in Dabie Mountain cattle and cold tolerance in Northeast Merino sheep [25,26]. The *PLCB1* gene has been associated with heat tolerance and adaptation to heat stress in the genomic profiles of Barki goats and sheep inhabiting the hot and arid coastal environment of Egypt’s Western Desert [27,28]. HIKESHI-mediated nuclear import plays a crucial role in attenuating and reversing the thermal stress response in human cells, as well as aiding recovery following cell injury due to heat shock [29]. Additionally, the *PITPNC1* gene facilitates thermogenesis in brown adipose tissue under acute cold conditions [30].

Ruoqiang *Lop sheep* primarily inhabit the wild, where local herders employ traditional grazing methods. This lifestyle has enhanced the adaptability of *Lop sheep* to the desert environment. Through enrichment analysis, several genes were identified, including *F13A1*, *PANX1*, *ST6GAL1*, *STXBP3*, and *ATP6V1A*. *F13A1*, a transglutaminase, plays a critical role in hemostasis and wound healing [31]. The *PANX1* gene modulates neural activity, both enhancing and inhibiting it under pathological conditions, such as ischemia, trauma, inflammation, and epilepsy, and is also essential for vascular function [32]. The *ST6GAL1* gene facilitates skin wound healing and reduces scar formation [33]. Additionally, the *STXBP3* gene is involved in platelet activation and secretion; its significance in sensorineural hearing loss and immune disorders has been established [34]. Lastly, *ATP6V1A* has been identified as a candidate gene associated with cerebral visual impairment (CVI) [35].

Currently, the population of *Lop sheep*, including the number of purebred individuals, continues to decline. Effective breed conservation and population expansion largely depend on high fecundity. Presently, the double lambing rate of *Lop sheep* is approximately 120%, with the majority of births being single. Through enrichment analysis, several genes influencing reproductive performance have been identified, including *RAP1B*, *RAB6A*, *PLCB1*, and *METTL15*, among others. The *RAP1B* gene was highlighted in studies examining the expression of fertility-regulating LncRNA in the ovaries of multiparous and single-parous Sal ewes, where gonadotropin-releasing hormone stimulates its expression in gonadotropins [36]. The *RAB6A* gene has been identified as a novel regulator of the meiotic apparatus and maturation processes in mouse oocytes, as well as a cell organelle involved in oocyte maturation, row organization, and cytoskeletal organization [37]. The *PLCB1* gene is implicated in cell proliferation, adhesion, growth, and survival, playing a significant role in regulating the reproductive performance of seasonal estrous sheep by influencing the expression and secretion of GnRH [38,39]. Furthermore, a whole-genome analysis of Guizhou black goats found the *METTL15* gene to be associated with reproductive performance [40]. Collectively, these genes directly or indirectly influence the fertility of *Lop sheep* by regulating ewe ovarian development, oocyte maturation, or hormone secretion. Therefore, identifying genes that control high reproductive traits is crucial for the breeding process of *Lop sheep* with elevated reproductive rates.

## 5. Conclusions

The genetic diversity of the *Lop sheep* population is substantial, and the degree of inbreeding is low. In comparison to the Yuli Lop sheep, the genetic diversity of the Ruoqiang *Lop sheep* population is slightly lower. The *Lop sheep* are categorized into two distinct groups: Ruoqiang and Yuli. Functional enrichment analysis has identified 16 candidate gene regions associated with immune response, 4 related to heat and cold resistance, 7 pertaining to desert adaptability, and 12 concerning reproductive performance in *Lop sheep.* These studies demonstrate the practical value of protecting and utilizing the genetic resources of *Lop sheep*.

## Figures and Tables

**Table 1:** Genetic diversity analysis of *Lop sheep*.

Collection Region	Number	He	Ho	π	Froh
Yuli Lop sheep	30	0.3030	0.2941	3.39 × 10^−3^	−0.0205
Ruoqiang Lop sheep	80	0.2992	0.2934	3.32 × 10^−3^	0.0547
total	120	0.3011	0.2938	3.36 × 10^−3^	0.0172

## Data Availability

The datasets analyzed during the current study are available, China National Center for Bioinformation/Beijing Institute of Genomics, Chinese Academy of Sciences repository, (https://ngdc.cncb.ac.cn/gvm/, accessed on 12 December 2024; GVM: GVM000916). In addition, we downloaded 11 Asiatic mouflon (PRJNA624020) at the National Center for Biotechnology Information (NCBI) (https://www.ncbi.nlm.nih.gov/, accessed on 12 December 2024).

## Supplementary Materials

The following supporting information can be downloaded at: https://www.mdpi.com/article/10.3390/biology14040337/s1, Figure S1: Kinship map of Ruoqiang and Yuli *Lop sheep.* Table S1: Statistics regarding the whole genome SNP variant types of RuoQiang and YuLi *Lop sheep* using ANNOVAR. Table S2: Principal component analysis of Yuli and Ruoqiang *Lop sheep*. Table S3: Neighbor-joining (NJ) tree depicting the relationships between the two groups of *Lop sheep* from Yuli and Ruoqiang. Table S4: ADMIXTURE results for Yuli and Ruoqiang *Lop sheep* are presented, showing population sizes k of 2, 3, and 5. Table S5: FST and π ratio select signal data. Table S6: GO and KEGG results. Table S7: Geographic location of Ruoqiang and Yuli *Lop sheep.*
