# Supplementary material for: Genetic Structure and Selection Signals for Extreme Environment Adaptation in Lop Sheep of Xinjiang
Source: Biology (Basel). 2025 Mar 25;14(4):337. doi: 10.3390/biology14040337 (PMC12025199; doi:10.3390/biology14040337)
Supplement: Supplementary file 1 [file biology-14-00337-s001.zip › Supplementary Table S1.pdf]

Mutation type		Yuli-Lop	Ruoqiang-Lop
Total SNP number		36,687,649	38,856,983
Number of annotated SNP		14,004,249	14,337,764
upstream		91,944	93,046
CDS	nonsynonymous	35,613	34,575
	stopgain	1,010	1,024
	stoploss	538	526
	synonymous	45,176	44,982
	unknown	4,282	4,196
downstream		101,395	103,760
upstream;downstream		3,288	3,222
UTR3		77,838	80,144
UTR5		26,324	27,323
UTR5;UTR3		100	131
intronic		4,792,634	4,958,835
exonic		87,698	86,638
exonic;splicing		12	6
ncRNA_exonic		48,747	49,111
ncRNA_exonic;splicing		2	2
ncRNA_intronic		351,989	361438
ncRNA_splicing		78	90
splicing		198	192
intergenic		8,422,001	8,573,825
