# Supplementary material for: Genetic Structure and Selection Signals for Extreme Environment Adaptation in Lop Sheep of Xinjiang
Source: Biology (Basel). 2025 Mar 25;14(4):337. doi: 10.3390/biology14040337 (PMC12025199; doi:10.3390/biology14040337)
Supplement: Supplementary file 1 [file biology-14-00337-s001.zip › Supplementary Table S2.pdf]

sample	group	PC1	PC2
0180	Ruoqiang-lop	0.034505	-0.003094
0934	Ruoqiang-lop	0.025196	0.026591
1112	Ruoqiang-lop	0.039034	-0.228388
1831	Ruoqiang-lop	0.035473	0.004404
1872	Ruoqiang-lop	0.027709	0.021485
1878	Ruoqiang-lop	0.035217	-0.004701
2040	Ruoqiang-lop	0.029262	0.014044
2102	Ruoqiang-lop	0.02787	0.020637
2103	Ruoqiang-lop	0.02904	-0.001446
2109	Ruoqiang-lop	0.030166	0.028098
2113	Ruoqiang-lop	0.021939	0.0382
2114	Ruoqiang-lop	0.036627	-0.027475
2133	Ruoqiang-lop	0.02844	-0.014469
2170	Ruoqiang-lop	0.041674	-0.234444
2389	Ruoqiang-lop	0.028887	0.020924
2392	Ruoqiang-lop	0.033617	0.01297
2425	Ruoqiang-lop	0.032203	0.030966
2440	Ruoqiang-lop	0.043275	-0.298219
2441	Ruoqiang-lop	0.031322	0.054495
2442	Ruoqiang-lop	0.030726	0.053932
2485	Ruoqiang-lop	0.028316	-0.000906
2578	Ruoqiang-lop	0.038344	-0.064701
2589	Ruoqiang-lop	0.023128	-0.184978
2592	Ruoqiang-lop	0.029974	0.027686
2610	Ruoqiang-lop	0.038529	-0.246844
2614	Ruoqiang-lop	0.037122	-0.019555
2624	Ruoqiang-lop	0.030147	0.017374
2626	Ruoqiang-lop	0.042316	-0.085241
2636	Ruoqiang-lop	0.019558	0.012953
2639	Ruoqiang-lop	0.036488	0.01178
2650	Ruoqiang-lop	0.03599	-0.006585
2656	Ruoqiang-lop	0.036953	-0.005362
2657	Ruoqiang-lop	0.024477	0.034275
2667	Ruoqiang-lop	0.029543	0.041134
2674	Ruoqiang-lop	0.021527	0.038318
2676	Ruoqiang-lop	0.032721	0.003996
2677	Ruoqiang-lop	0.031184	0.001872
2678	Ruoqiang-lop	0.035937	-0.023708
2682	Ruoqiang-lop	0.032748	0.027946
2691	Ruoqiang-lop	0.045665	-0.246665
2693	Ruoqiang-lop	0.032986	3.63E-06
2694	Ruoqiang-lop	0.030014	0.007784
2696	Ruoqiang-lop	0.029295	0.027345

2697	Ruoqiang-lop	0.033516	0.029406
2698	Ruoqiang-lop	0.027163	0.032598
2701	Ruoqiang-lop	0.038633	-0.036362
2703	Ruoqiang-lop	0.028317	0.010246
2704	Ruoqiang-lop	0.038917	-0.059032
2708	Ruoqiang-lop	0.026801	-0.242957
2762	Ruoqiang-lop	0.032605	0.009776
2765	Ruoqiang-lop	0.028299	0.014526
2779	Ruoqiang-lop	0.015742	0.022601
2781	Ruoqiang-lop	0.034913	-0.005353
2784	Ruoqiang-lop	0.032943	-0.001976
2785	Ruoqiang-lop	0.027868	0.015564
2787	Ruoqiang-lop	0.02476	0.004543
2789	Ruoqiang-lop	0.027503	0.031707
2791	Ruoqiang-lop	0.035502	0.001034
2793	Ruoqiang-lop	0.027622	0.024415
2794	Ruoqiang-lop	0.027506	0.029881
2919	Ruoqiang-lop	0.030519	0.020875
2949	Ruoqiang-lop	0.026467	0.014312
2955	Ruoqiang-lop	0.028624	0.026056
2958	Ruoqiang-lop	0.040168	-0.326907
3077	Ruoqiang-lop	0.030639	0.008893
3082	Ruoqiang-lop	0.034089	0.004971
3087	Ruoqiang-lop	0.030158	0.027439
3089	Ruoqiang-lop	0.024877	0.027057
3095	Ruoqiang-lop	0.021911	0.034738
3101	Ruoqiang-lop	0.026815	0.034029
3364	Ruoqiang-lop	0.030052	0.00236
3390	Ruoqiang-lop	0.03321	0.022997
3392	Ruoqiang-lop	0.035743	-0.00543
3399	Ruoqiang-lop	0.034194	-0.020637
3416	Ruoqiang-lop	0.039639	-0.02172
3420	Ruoqiang-lop	0.036899	-0.027589
3432	Ruoqiang-lop	0.045994	-0.350037
3634	Ruoqiang-lop	0.026529	0.048
3650	Ruoqiang-lop	0.046236	-0.350649
7899	Ruoqiang-lop	0.037657	-0.025092
SD20231103080	Yuli-lop	0.020488	0.10953
SD20231103081	Yuli-lop	0.022022	0.105535
SD20231103082	Yuli-lop	0.016965	0.054561
SD20231103083	Yuli-lop	0.020305	0.049309
SD20231103084	Yuli-lop	0.019505	0.060211
SD20231103085	Yuli-lop	0.019451	0.06594
SD20231103086	Yuli-lop	0.019834	0.074808

SD20231103087	Yuli-lop	0.017314	0.054151
SD20231103088	Yuli-lop	0.022196	0.10752
SD20231103089	Yuli-lop	0.017759	0.078506
SD20231103090	Yuli-lop	0.020368	0.081527
SD20231103091	Yuli-lop	0.018764	0.08203
SD20231103092	Yuli-lop	0.02141	0.083387
SD20231103093	Yuli-lop	0.0207	0.051989
SD20231103094	Yuli-lop	0.016652	0.076312
SD20231103096	Yuli-lop	0.019832	0.079306
SD20231103097	Yuli-lop	0.017295	0.069216
SD20231103098	Yuli-lop	0.021344	0.100587
SD20231103099	Yuli-lop	0.019253	0.097946
SD20231103100	Yuli-lop	0.020227	0.084696
SD20231103101	Yuli-lop	0.01973	0.082252
SD20231103102	Yuli-lop	0.020399	0.073968
SD20231103103	Yuli-lop	0.02119	0.076103
SD20231103104	Yuli-lop	0.020098	0.065818
SD20231103105	Yuli-lop	0.021294	0.060892
SD20231103106	Yuli-lop	0.017191	0.044858
SD20231103107	Yuli-lop	0.017453	0.06665
SD20231103108	Yuli-lop	0.019984	0.086779
SD20231103109	Yuli-lop	0.01986	0.082858
SD20231103110	Yuli-lop	0.018879	0.063144
SRR11657500	Asiatic mouflon	-0.2757	-0.013654
SRR11657501	Asiatic mouflon	-0.298112	-0.015902
SRR11657502	Asiatic mouflon	-0.296802	-0.018139
SRR11657503	Asiatic mouflon	-0.282921	-0.016322
SRR11657504	Asiatic mouflon	-0.296572	-0.017888
SRR11657505	Asiatic mouflon	-0.268425	-0.012927
SRR11657506	Asiatic mouflon	-0.274978	-0.013875
SRR11657507	Asiatic mouflon	-0.298685	-0.019043
SRR11657635	Asiatic mouflon	-0.266257	-0.013185
SRR11657640	Asiatic mouflon	-0.296799	-0.01893
SRR11657642	Asiatic mouflon	-0.295147	-0.017333

	PCA(%)
PC1	7.25463
PC2	2.9233
