# Supplementary material for: Genetic Structure and Selection Signals for Extreme Environment Adaptation in Lop Sheep of Xinjiang
Source: Biology (Basel). 2025 Mar 25;14(4):337. doi: 10.3390/biology14040337 (PMC12025199; doi:10.3390/biology14040337)
Supplement: Supplementary file 1 [file biology-14-00337-s001.zip › Supplementary Table S3.pdf]

(((((((((((((((2919:0.11780547,((2698:0.10480542,2425:0.10401458):0.00430036,2109:0.1084106
4):0.01060728):0.00752227,2592:0.12532598):0.00075187,(2794:0.12573512,((2682:0.11226094
,2678:0.10605606):0.01829296,2102:0.12617004):0.00091663):0.00067236):0.00012417,(((((339
2:0.12119783,((((3082:0.10777220,1878:0.10303780):0.01172463,2614:0.11231887):0.00980227
,2701:0.11901773):0.00176156,2103:0.12417294):0.00067717):0.00155671,((((7899:0.11554095,
2762:0.11870005):0.00449397,2392:0.12313803):0.00108432,(3416:0.11638804,2677:0.1201009
6):0.00532093):0.00052069,(((((3390:0.09015161,2636:0.02921539):0.04311479,2779:0.0899172
1):0.01583671,2133:0.10225729):0.00486241,3364:0.12119334):0.00216666,0180:0.12386766):0
.00101911):0.00038862):0.00067064,((((2955:0.10376840,0934:0.10360660):0.02088555,2694:0.
12153245):0.00229032,2793:0.12687743):0.00086353,2485:0.12570159):0.00055964):0.0004415
9,((((((((3650:0.01923603,3432:0.01834597):0.08314651,2610:0.10171699):0.00444653,(2170:0.
10970486,1112:0.10121814):0.00572285):0.00514839,(2589:0.06487973,2440:0.06669927):0.03
862267):0.00504468,((2958:0.05372596,2708:0.05055104):0.03427591,2691:0.09033909):0.019
52924):0.01093780,((3420:0.01724589,2114:0.01672211):0.10552977,(2704:0.10848582,(2626:0
.09799225,2578:0.10226075):0.00185368):0.01177423):0.00281369):0.00285829,(2791:0.100911
21,2693:0.10213079):0.02296523):0.00042812,(((3399:0.10868913,2785:0.11101487):0.0153380
0,((2789:0.11511589,2639:0.10202211):0.00943802,2656:0.12309348):0.00459200):0.00112033,(
(((3077:0.12236352,2696:0.11963448):0.00091622,(2389:0.10681929,1831:0.10568871):0.01513
803):0.00357160,(2676:0.12210025,(2442:0.01806859,2441:0.01912341):0.10354025):0.001800
77):0.00132046,2040:0.12603342):0.00057725):0.00050770):0.00033428):0.00024004,(((((3634:
0.10693504,2667:0.10280496):0.01650482,2697:0.11208418):0.00744755,(3087:0.11580021,262
4:0.11705879):0.00501908):0.00360483,2787:0.12587211):0.00121196,(((2784:0.10520376,2765
:0.10654724):0.00081579,2650:0.10511821):0.01879700,(2781:0.12399123,1872:0.12563177):0.
00018538):0.00127988):0.00031596):0.00016451):0.00039248,(3089:0.12679011,(2949:0.12689
913,2703:0.12422587):0.00084239):0.00055127):0.00032506,(3101:0.12735564,(2657:0.124990
22,2113:0.12591878):0.00019036):0.00026327):0.00053365,3095:0.12593921):0.00016846,2674:
0.12750621):0.00161741,(3106:0.13018362,((((((3107:0.12494340,3103:0.11916260):0.0081951
3,((3104:0.11584098,(3092:0.10195694,3090:0.10746706):0.01232102):0.01187447,(3102:0.120
43921,3097:0.12294379):0.00612341):0.00177050):0.00085192,(((((3108:0.10392272,3099:0.10
819628):0.00691121,3091:0.11564629):0.00339868,3086:0.11981832):0.00525448,3094:0.12178
814):0.00766256,(((3101:0.10638140,3100:0.10719360):0.01543783,3089:0.11813617):0.008439
81,3084:0.12701519):0.00276406):0.00029241):0.00024944,(((3110:0.12505048,3087:0.1294025
2):0.00293712,3105:0.12999888):0.00063155,(((3109:0.11106267,3098:0.10888033):0.00707655
,(3088:0.10716610,(3081:0.10663073,1103:0.10692427):0.00417290):0.00940220):0.00962102,(
3096:0.11418330,3085:0.12314470):0.00718923):0.00399651):0.00025286):0.00061066,(3093:0.
13441415,3083:0.13029385):0.00060578):0.00031436,3082:0.13203275):0.00025750):0.0017669
4):0.04754323,(5763:0.11314730,7505:0.11197370):0.00182872):0.00255544,7506:0.11072673):
0.00052873,(7507:0.07094673,7501:0.07065927):0.04007432):0.00156705,7503:0.10446365):0.0
0408254,(7504:0.11441894,(5764:0.11119391,7502:0.10413009):0.00044506):0.00112343):0.000
65720,5750:0.10819138,7642:0.10364162);
