# Supplementary material for: Genetic Structure and Selection Signals for Extreme Environment Adaptation in Lop Sheep of Xinjiang
Source: Biology (Basel). 2025 Mar 25;14(4):337. doi: 10.3390/biology14040337 (PMC12025199; doi:10.3390/biology14040337)
Supplement: Supplementary file 1 [file biology-14-00337-s001.zip › Supplementary Table S4.pdf]

K=2	
0.589127	0.410873
0.713034	0.286966
0.00001	0.99999
0.567789	0.432211
0.690002	0.309998
0.56086	0.43914
0.650315	0.349685
0.692577	0.307423
0.576355	0.423645
0.690412	0.309588
0.76036	0.23964
0.00001	0.99999
0.568081	0.431919
0.00001	0.99999
0.664975	0.335025
0.631147	0.368853
0.706678	0.293322
0.00001	0.99999
0.798785	0.201215
0.797755	0.202245
0.613337	0.386663
0.294373	0.705627
0.308056	0.691944
0.685936	0.314064
0.00001	0.99999
0.487095	0.512905
0.663824	0.336176
0.027905	0.972095
0.673149	0.326851
0.62864	0.37136
0.54546	0.45454
0.562747	0.437253
0.739274	0.260726
0.75545	0.24455
0.759271	0.240729
0.636295	0.363705
0.616941	0.383059
0.473769	0.526231
0.687176	0.312824
0.00001	0.99999
0.58938	0.41062
0.653024	0.346976
0.703517	0.296483

0.703375	0.296625
0.719566	0.280434
0.412904	0.587096
0.650563	0.349437
0.303014	0.696986
0.156442	0.843558
0.627217	0.372783
0.646624	0.353376
0.698751	0.301249
0.566639	0.433361
0.57913	0.42087
0.688394	0.311606
0.665431	0.334569
0.723554	0.276446
0.558699	0.441301
0.699228	0.300772
0.707515	0.292485
0.675594	0.324406
0.685806	0.314194
0.698834	0.301166
0.00001	0.99999
0.62115	0.37885
0.603513	0.396487
0.713755	0.286245
0.710548	0.289452
0.739076	0.260924
0.729682	0.270318
0.628937	0.371063
0.687975	0.312025
0.559202	0.440798
0.52601	0.47399
0.494953	0.505047
0.00001	0.99999
0.00001	0.99999
0.787739	0.212261
0.00001	0.99999
0.510779	0.489221
0.99999	0.00001
0.99999	0.00001
0.849523	0.150477
0.840197	0.159803
0.878299	0.121701
0.87129	0.12871
0.929391	0.070609

0.84987	0.15013
0.99999	0.00001
0.935847	0.064153
0.975146	0.024854
0.985176	0.014824
0.99999	0.00001
0.844803	0.155197
0.93579	0.06421
0.9185	0.0815
0.91417	0.08583
0.99999	0.00001
0.99999	0.00001
0.981389	0.018611
0.967334	0.032666
0.941015	0.058985
0.939432	0.060568
0.900727	0.099273
0.879992	0.120008
0.814681	0.185319
0.902074	0.097926
0.999188	0.000812
0.943167	0.056833
0.880201	0.119799

K=3		
0.811727	0.063533	0.124741
0.727067	0.026513	0.24642
0.046096	0.953894	0.00001
0.99998	0.00001	0.00001
0.693976	0.043514	0.26251
0.901837	0.041419	0.056744
0.77408	0.049056	0.176864
0.706901	0.044797	0.248302
0.777257	0.06312	0.159622
0.872347	0.000617	0.127035
0.603419	0.027875	0.368706
0.99998	0.00001	0.00001
0.801155	0.084818	0.114027
0.00001	0.99998	0.00001
0.879448	0.00001	0.120542
0.878534	0.016307	0.105159
0.904423	0.00001	0.095567
0.00001	0.99998	0.00001
0.99998	0.00001	0.00001

0.99998	0.00001	0.00001
0.737601	0.079929	0.18247
0.847042	0.152948	0.00001
0.41786	0.492271	0.089869
0.803248	0.013326	0.183426
0.00001	0.99998	0.00001
0.93963	0.06036	0.00001
0.839142	0.01893	0.141928
0.865112	0.134878	0.00001
0.836041	0.022871	0.141088
0.99998	0.00001	0.00001
0.976468	0.023522	0.00001
0.907614	0.057744	0.034642
0.625336	0.036134	0.33853
0.752261	0.00001	0.247729
0.578647	0.036792	0.384561
0.845124	0.06044	0.094436
0.822135	0.061122	0.116743
0.92321	0.062927	0.013863
0.851696	0.00001	0.148294
0.220323	0.779667	0.00001
0.875531	0.038006	0.086463
0.795298	0.044643	0.16006
0.84019	0.00001	0.1598
0.868749	0.00001	0.131241
0.803632	0.007486	0.188882
0.913306	0.086684	0.00001
0.741343	0.047938	0.210719
0.8798	0.12019	0.00001
0.189976	0.726722	0.083303
0.858277	0.029669	0.112054
0.833093	0.022508	0.144399
0.770064	0.024837	0.205099
0.861379	0.04742	0.091202
0.989206	0.010784	0.00001
0.762996	0.038766	0.198237
0.613545	0.104865	0.281591
0.832319	0.00001	0.167671
0.99998	0.00001	0.00001
0.691758	0.038472	0.26977
0.772671	0.018557	0.208772
0.810141	0.021596	0.168263
0.670592	0.062222	0.267186
0.752413	0.028007	0.219581

0.00001	0.99998	0.00001
0.892397	0.014222	0.093381
0.954774	0.013785	0.03144
0.75112	0.020904	0.227977
0.653403	0.046074	0.300524
0.621455	0.038715	0.33983
0.666111	0.029244	0.304645
0.776109	0.057671	0.166221
0.891376	0.00001	0.108614
0.914871	0.036898	0.048231
0.894475	0.076475	0.029049
0.940669	0.059321	0.00001
0.99998	0.00001	0.00001
0.00001	0.99998	0.00001
0.685206	0.00001	0.314784
0.00001	0.99998	0.00001
0.893258	0.101254	0.005487
0.00001	0.00001	0.99998
0.00001	0.00001	0.99998
0.354076	0.024769	0.621155
0.34793	0.032131	0.61994
0.279017	0.025041	0.695942
0.30207	0.013434	0.684497
0.17574	0.00878	0.81548
0.350189	0.023217	0.626593
0.00001	0.00001	0.99998
0.146161	0.014775	0.839063
0.057577	0.007056	0.935367
0.00001	0.006797	0.993193
0.00001	0.00001	0.99998
0.333835	0.03113	0.635034
0.160998	0.012848	0.826155
0.210398	0.005468	0.784133
0.213273	0.014655	0.772073
0.00001	0.00001	0.99998
0.00001	0.00001	0.99998
0.055497	0.000023	0.944481
0.106794	0.00001	0.893196
0.17539	0.00049	0.82412
0.112684	0.022622	0.864694
0.188608	0.031859	0.779533
0.270882	0.020721	0.708397
0.409325	0.031697	0.558978
0.208835	0.026129	0.765036

0.00001	0.00001	0.99998		
0.062414	0.029515	0.908071		
0.282906	0.020323	0.696771		

K=5				
0.739918	0.037106	0.073992	0.134402	0.014582
0.810636	0.020189	0.159936	0.00001	0.009229
0.220109	0.76711	0.00001	0.00001	0.012761
0.715613	0.00001	0.00001	0.238086	0.046281
0.680067	0.029932	0.204701	0.067859	0.017441
0.853519	0.017207	0.00001	0.129253	0.00001
0.702725	0.039926	0.120739	0.070294	0.066316
0.675634	0.033649	0.190531	0.065021	0.035166
0.646806	0.033734	0.128969	0.182275	0.008217
0.936207	0.00001	0.027212	0.00001	0.036561
0.627445	0.02239	0.301976	0.024724	0.023466
0.00001	0.00001	0.00001	0.99996	0.00001
0.724235	0.054026	0.067165	0.135461	0.019113
0.208182	0.791788	0.00001	0.00001	0.00001
0.763237	0.00001	0.081138	0.102841	0.052774
0.9108	0.000259	0.02638	0.054603	0.007958
0.964766	0.00001	0.00001	0.00001	0.035204
0.00001	0.99996	0.00001	0.00001	0.00001
0.00001	0.00001	0.00001	0.00001	0.99996
0.00001	0.00001	0.00001	0.00001	0.99996
0.745528	0.062332	0.120491	0.066448	0.005201
0.00001	0.00001	0.00001	0.99996	0.00001
0.395436	0.458906	0.055942	0.076549	0.013168
0.823968	0.005044	0.11234	0.041156	0.017491
0.165036	0.834934	0.00001	0.00001	0.00001
0.776415	0.026224	0.00001	0.194495	0.002855
0.864045	0.010387	0.06286	0.030357	0.032351
0.00001	0.00001	0.00001	0.99996	0.00001
0.99495	0.00502	0.00001	0.00001	0.00001
0.896409	0.00001	0.00001	0.011104	0.092467
0.875805	0.00067	0.00001	0.123506	0.00001
0.889108	0.030703	0.00001	0.067716	0.012464
0.588172	0.030355	0.283806	0.052609	0.045058
0.858351	0.00001	0.118909	0.00001	0.02272
0.594561	0.031776	0.322905	0.02499	0.025767
0.786287	0.056989	0.023758	0.022061	0.110905
0.824168	0.047384	0.055158	0.052544	0.020746
0.590237	0.00001	0.00001	0.409733	0.00001
0.82798	0.00001	0.08654	0.081333	0.004137

0.00001	0.524672	0.00001	0.475298	0.00001
0.773009	0.018583	0.047183	0.142136	0.01909
0.830754	0.033066	0.084514	0.030971	0.020695
0.779603	0.00001	0.100897	0.048997	0.070492
0.982646	0.000014	0.00001	0.00001	0.017321
0.883614	0.002559	0.095775	0.00001	0.018042
0.734497	0.03456	0.00001	0.230923	0.00001
0.708472	0.033854	0.157861	0.078521	0.021292
0.00001	0.00001	0.00001	0.931165	0.068805
0.192259	0.657324	0.053455	0.096952	0.00001
0.874524	0.018027	0.040654	0.0554	0.011395
0.837185	0.01543	0.0919	0.050939	0.004547
0.890825	0.010889	0.08431	0.003795	0.010181
0.799926	0.028635	0.040173	0.131255	0.00001
0.940624	0.00001	0.00001	0.059346	0.00001
0.698345	0.034363	0.14867	0.048114	0.070508
0.65592	0.09041	0.216261	0.020727	0.016682
0.772523	0.001105	0.107287	0.00001	0.119075
0.836161	0.00001	0.00001	0.163809	0.00001
0.712362	0.029163	0.205551	0.039766	0.013157
0.826179	0.011467	0.131301	0.014364	0.016689
0.85023	0.011801	0.090676	0.025616	0.021677
0.694634	0.05084	0.199662	0.032701	0.022163
0.829049	0.021075	0.133441	0.000717	0.015718
0.00001	0.99996	0.00001	0.00001	0.00001
0.729645	0.001672	0.056437	0.14575	0.066496
0.930333	0.00001	0.00001	0.06458	0.005067
0.791688	0.013758	0.147891	0.010484	0.036179
0.665424	0.035917	0.240231	0.041888	0.01654
0.622497	0.030976	0.279064	0.042835	0.024628
0.691101	0.021618	0.236729	0.033531	0.017021
0.772783	0.042313	0.103206	0.068562	0.013136
0.99996	0.00001	0.00001	0.00001	0.00001
0.856089	0.012503	0.00001	0.131387	0.00001
0.690386	0.047458	0.003156	0.205609	0.05339
0.809106	0.023233	0.00001	0.167641	0.00001
0.00001	0.00001	0.00001	0.99996	0.00001
0.00001	0.99996	0.00001	0.00001	0.00001
0.792903	0.00001	0.189618	0.00001	0.017459
0.00001	0.99996	0.00001	0.00001	0.00001
0.821361	0.073276	0.00001	0.105343	0.00001
0.00001	0.00001	0.99996	0.00001	0.00001
0.00001	0.00001	0.99996	0.00001	0.00001
0.373038	0.021096	0.583567	0.008741	0.013558

0.363583	0.027456	0.580698	0.015665	0.012597
0.310543	0.021985	0.661242	0.006105	0.000125
0.283106	0.007348	0.659377	0.042644	0.007525
0.175828	0.006437	0.795164	0.014723	0.007848
0.352058	0.020229	0.592006	0.020658	0.015049
0.00001	0.00001	0.99996	0.00001	0.00001
0.179288	0.012592	0.8081	0.00001	0.00001
0.084631	0.005363	0.907796	0.00001	0.002201
0.00001	0.001047	0.990107	0.000837	0.007998
0.00001	0.00001	0.99996	0.00001	0.00001
0.34113	0.025033	0.600268	0.024364	0.009205
0.165218	0.011792	0.805319	0.004871	0.0128
0.19073	0.002672	0.767031	0.029626	0.00994
0.234459	0.011895	0.741157	0.005588	0.006901
0.00001	0.00001	0.99996	0.00001	0.00001
0.00001	0.00001	0.99996	0.00001	0.00001
0.080787	0.00001	0.915912	0.00001	0.003281
0.129159	0.00001	0.862983	0.00001	0.007839
0.207221	0.00001	0.791565	0.001194	0.00001
0.142638	0.018303	0.835996	0.00001	0.003053
0.209595	0.027264	0.752988	0.010143	0.00001
0.27407	0.016343	0.679254	0.020685	0.009648
0.404137	0.026319	0.520805	0.027513	0.021225
0.226151	0.024306	0.736089	0.007543	0.005911
0.00001	0.00001	0.99996	0.00001	0.00001
0.060334	0.026034	0.903033	0.004688	0.005911
0.292206	0.017007	0.665056	0.013604	0.012126
