# Supplementary material for: Genetic Structure and Selection Signals for Extreme Environment Adaptation in Lop Sheep of Xinjiang
Source: Biology (Basel). 2025 Mar 25;14(4):337. doi: 10.3390/biology14040337 (PMC12025199; doi:10.3390/biology14040337)
Supplement: Supplementary file 1 [file biology-14-00337-s001.zip › Supplementary Table S5.pdf]

θπ	Fst
SASH1	CASZ1
SLC36A3	PEX14
SLC36A2	TNK2
TUBGCP5	TFRC
CCDC115	LOC121818611
IMP4	PRPF38B
PTPN18	FNDC7
MINDY2	STXBP3
ADAM10	FBLN5
STAU2	TRIP11
C9H8orf89	RAMP1
RDH10	RBM44
RPL7	LRRFIP1
GLDN	RNLS
NIPA1	SLC30A4
NIPA2	LOC114115866
CYFIP1	C7H15orf48
UBOX5	SPATA5L1
AVP	MAPKAP1
OXT	ST6GALNAC3
MRPS26	LOC101123368
PTPRA	HSP90B1
ZEB1	C3H12orf73
DYRK1A	LYPD6B
FASTKD5	CA13
MROH9	E2F5
LOC101104165	RBIS
CCL22	LOC105614203
CX3CL1	SECISBP2
RALY	SEMA4D
EIF2S2	NUP107
RD3	RAP1B
TRAF5	COL21A1
NBEAL1	THRAP3
SEMA3D	SH3D21
KHDRBS1	STK40
LNPK	EVA1B
LZTS3	LSM10
PPCDC	OSCP1
SCAMP5	TNRC6A
PARN	SLC5A11
LTBP1	GLI2
ABCC11	SHISAL1

LONP2	RAB2A
RALGAPA1	ENAH
BRMS1L	MICU1
PRUNE2	LOC114116451
ZC3H13	TMA16
CPB2	TKTL2
LOC101107331	TLL1
STOX2	AMD1
YTHDC1	CDK19
LOC101116729	ANTXR2
UGT2B7	RNF152
CDH24	ST7
PSMB11	EIF3H
PSMB5	NEGR1
C7H14orf93	HMGXB4
AJUBA	SNX19
HAUS4	LOC114109370
BFAR	ZNF703
LOC114110613	ERLIN2
NFAT5	PLPBP
FAM219B	SLC12A2
MPI	DNAH5
SCAMP2	ABCD3
ULK3	TUBGCP6
CPLX3	HDAC10
LMAN1L	MAPK12
CSK	MAPK11
DOCK7	PLXNB2
USP1	DENND6B
COX5A	VAT1L
GLS	KCNMA1
STAT1	ETV6
NELL2	SYNPO
OAZ2	MYOZ3
ZNF609	RBM22
ZER1	DCTN4
ZDHHC12	WRN
PKN3	LOC114114858
SET	GTF3C6
DYNC2I2	STARD13
SPTAN1	LOC101105418
OCIAD1	LOC101105666
CPB1	LOC101123629
RPS20	CHSY1

MOS	NCAM1
PLAG1	PALMD
AFAP1L2	FRRS1
FOXB2	DYTN
NRXN2	MDH1B
RASGRP2	ZDHHC21
PYGM	XYLT1
SF1	CFAP91
MAP4K2	ILDR2
MEN1	RAVER2
TSGA10	RFLNB
C3H2orf15	C11H17orf97
PSD	RPH3AL
FBXL15	ADAM23
CUEDC2	UTRN
C22H10orf95	BAALC
MFSD13A	PHLPP1
ACTR1A	ADAMTS19
SUFU	ANLN
SLC17A6	KIAA0895
LOC105607887	ABCB1
PHYH	LOC101110128
SEPHS1	PDE11A
LOC101122880	SMAD7
MARK3	MAP3K21
DENND5A	LOC101107864
SCUBE2	CRTC1
NAA35	F13A1
KCNB1	NRN1
VTI1A	TACC2
OCIAD2	METTL15
NOP9	HIP1
LTB4R	CCL26
DHRS1	CCL24
CIDEB	RGS12
TGM1	PDZRN4
RABGGTA	ATXN10
NEDD8	WNT7B
TINF2	MAS1
GMPR2	IGF2R
LOC101115461	SLC9A9
CHMP4A	SMARCA2
TSSK4	PARD3
TM9SF1	LOC114117880

MDGA2	LOC101117184
TRIM35	LOC101117691
STMN4	LOC101117955
SASS6	MT3
TRMT13	BBS2
LRRC39	MT4
DBT	C22H10orf90
RFX4	LOC101123223
LOC101116302	LOC101106447
TCF12	F13B
NDUFV2	CFHR5
ANKRD12	ASPM
PANX1	SGPP2
HEPHL1	AK4
ACIN1	DNAJC6
RSPO2	CD109
ELMO1	CEP350
COL19A1	QSOX1
CARD6	UBXN4
RPL37	R3HDM1
PRKAA1	LOC101119992
TTC33	LOC121820152
BBX	ANO3
GRM8	SLC5A12
JAZF1	COP1
MED27	BAHCC1
NDUFA4	LOC101102503
PHF14	IFT43
LOC105611203	TGFB3
CD14	ZNF330
TMCO6	CPD
NDUFA2	GOSR1
IK	AAK1
WDR55	LOC101108521
DND1	ATP10B
LOC101121599	WNT4
HARS1	ACTN4
ZMAT2	CAPN12
LOC101122108	LGALS7
STX18	ADAMTS3
TRPM6	CLEC5A
LYPD2	TAS2R38
THEM6	MGAM
SLURP1	GLCE

PSCA	PAQR5
JRK	YAP1
ARC	TMEM156
LOC101108600	KLHL5
LOC101108857	PRKG1
LOC101114553	BAZ2A
LOC101115061	RBMS2
LOC101114807	GLS2
LOC101115307	CDC14B
LOC101115567	LOC114112940
GALM	LOC114112781
CD55	SRGAP2
LOC101115822	DOCK1
LOC101109119	LOC101104148
LOC101109079	LOC101104659
EFNA5	LOC105610506
PRIM2	LOC101104906
KCTD3	LOC105610505
GPR156	LOC101105656
LGR5	LOC105611918
MCMDC2	ATP6V1C1
LOC121820309	PARP12
TCF24	TBXAS1
PPP1R42	KDM7A
EIF5	LOC101121515
VWA5B1	LOC101122025
COPS5	LOC114113586
CSPP1	CATSPERE
SOX12	ADSS2
C13H20orf96	C12H1orf100
ZCCHC3	IL20RA
DEFB129	NTM
LDLRAD3	NDUFA9
TWSG1	AKAP3
LOC101119226	DYRK4
PRKCH	CPXM2
CCP110	CHST15
GDE1	PUS3
VPS35L	HYLS1
ATG2A	DDX25
PPP2R5B	VSIG10L2
GPHA2	CDON
MAJIN	LRRC39
BATF2	DBT

DGLUCY	RTCA
NSF	LOC101102091
LOC101120590	DAO
C2CD3	SSH1
PPME1	PTPN2
TAS2R40	SEH1L
GSTK1	CEP192
TMEM139	DHX36
CASP2	ARHGEF26
KIF3C	UBE2U
LOC101109692	APH1B
RAB10	RAB8B
ADAMTS3	MAN2A1
ATR	CNGB1
XRN1	LOC101121702
PHKB	ZNF319
LOC101113467	USB1
IMPG1	RAPGEF4
CDH26	KCNG3
FAM217B	LOC101115694
PPP1R3D	EML4
SYCP2	TDRD7
RMDN3	LOC105602084
GCHFR	ZNF613
DNAJC17	LOC101103836
C7H15orf62	ZNF614
ZFYVE19	KCNQ3
PPP1R14D	HHLA1
SPINT1	OC90
ATP8B4	EFR3A
LOC101113787	CSN1S2
TRIP12	NMU
IL20RB	KCNH1
NCK1	NKAIN3
SCN3A	ZNF516
TCF7L1	KCNB2
TGOLN2	LOC101104594
RETSAT	CST7
ELMOD3	ACSS1
SLC35F1	TNIP3
XRRA1	ARHGEF12
SPCS2	LOC121816658
UBE3A	CDH20
MYO5C	GPR158

GNB5	RNF180
BCL2L10	SDK1
LOC101105840	FJX1
NET1	TRIM71
RHOV	SLC12A8
TMEM114	ZNF148
METTL22	FAM71A
NDUFS4	ATF3
CREB3L2	MRLN
LOC101118606	CCDC6
STK17A	LOC114110154
HDAC9	LOC101110889
VWA2	LOC101111139
INSIG2	DENND1A
CCDC88A	TLL2
ZCCHC10	RALGPS1
HSPA4	ANGPTL2
MFN1	SNRNP48
ZNF639	AUH
MADD	KLK6
MYBPC3	KLK5
SPI1	KLK7
LOC121816623	KLK8
SLC39A13	KLK9
DCTN1	LOC101113003
LOC101116640	KLK11
C3H2orf81	KLK10
WDR54	KLK13
RTKN	KLK14
STXBP5L	RAB6A
SDR42E1	MRPL48
HSD17B2	FBXO28
LOC101117342	IL27
LOC101111528	NUPR1
PRKAG3	SGF29
WNT6	LOC101114075
WNT10A	BOLA2B
LOC101121753	SLX1A
C16H5orf22	CORO1A
DROSHA	RASGEF1B
CEP350	TGFA
LOC101103112	C13H20orf96
RHOA	ZCCHC3
GPX1	DEFB129

USP4	RTL6
C19H3orf62	ABTB2
IHO1	CAT
FBXL17	SOD3
AKAP9	LOC101115632
LOC101110611	NUP58
MARVELD2	MDGA2
RAD17	ZNF423
TAF9	TPRG1
AK6	SCUBE1
CCDC125	ARID3C
CDK7	SIGMAR1
DNM1	RPP25L
CIZ1	DCTN3
LCN2	CNTFR
BBLN	TTLL11
PTGES2	SH3PXD2A
SLC25A25	STN1
TCERG1	LOC101120042
GPR151	ADAP1
ZNF286A	GET4
LOC105611617	SUN1
ZNF287	DCST2
LOC101104050	DCST1
COL13A1	ADAM15
LOC101120408	EFNA4
TACC2	EFNA3
BTBD16	EFNA1
BCL11A	BMPR1A
EMC2	MMRN2
KNTC1	ADIRF
SCAMP1	SNCG
LHFPL2	FAM25A
SRSF7	VPS41
SLC34A2	LOC101111593
PIK3R1	OSBPL10
LOC101122678	PICALM
LOC101117745	LOC101109747
LOC101114847	BTNL2
TMEM50A	LOC101110006
RSRP1	LOC101110277
MIPOL1	LOC101103860
FOXA1	C22H10orf82
NUDT4	HSPA12A

LOC114111177	PANX1
HEG1	FAM81B
NOVA1	RPS6KL1
PIBF1	PGF
KLF5	LOC114115884
MFNG	EIF2B2
CARD10	SLC6A16
RUFY2	CD37
DNA2	TEAD2
SLC25A16	DKKL1
MYO5A	PTPN5
AHCTF1	IGSF22
LOC105607283	TMEM86A
AKAP6	PTBP2
HEATR5A	MED26
DTD2	SLC35E1
LOC106991193	LOC101117160
LOC101122274	C5H19orf44
DHX15	LOC101115589
PLAA	SLC25A13
IFT74	MYH15
LRRC19	ATRNL1
SOX6	CNTNAP2
LMX1B	LRIG1
ARL2	DENND5B
LOC114115604	HAO2
P4HA3	HSD3B1
PGM2L1	DNAJC13
SCCPDH	LOC101119869
LOC101118398	ATP10A
CASQ1	GAREM1
PEA15	GXYLT2
DCAF8	SHQ1
PEX19	LOC105609282
COPA	MAGI1
MED23	FEZ2
ENPP3	PARD3B
RNF182	SH3BP5
KIAA1328	CNOT10
LOC101107359	TRIM36
LRRC75A	PGGT1B
WWC1	ANK2
RECK	SYTL2
LOC101111518	CCDC89

LOC101111773	CREBZF
LOC105607666	TMEM126A
LOC114112799	TMEM126B
LOC114112800	DLG2
LOC101108371	LOC101108695
METTL13	IMMP2L
DNM3	LOC101119202
MET	RNF157
GRIK2	FOXJ1
GRM1	EXOC7
NFATC1	GALR2
ATP9B	ZACN
CCL21	SRP68
CCL19	HMGA2
LOC114112952	KPNA7
CCL27	SMURF1
IL11RA	DERA
SIGMAR1	LOC101104225
GALT	CPPED1
ARID3C	RRP12
RPP25L	PGAM1
DCTN3	ZDHHC16
ALKBH3	EXOSC1
C15H11orf96	MMS19
LOC101110483	UBTD1
UBE3D	LOC101120595
DOP1A	ERGIC3
LOC114115358	LOC101117181
TMEM60	SPAG4
RSBN1L	CPNE1
LOC101102976	LOC105606903
ZNF134	DYNLRB2
LOC101115820	CDYL2
LOC105602036	OGDHL
LOC105602037	PARG
LOC101116597	LOC101103187
LOC114108630	LRRC66
RAB11FIP4	SGCB
NF1	SPATA18
THBD	FOXB2
STXBP6	LOC101114620
BSDC1	KAT6B
FAM229A	KCNK1
TSSK3	MAP3K7

HDAC1	XKR4
MARCKSL1	TMEM68
PRDM16	CRPPA
ERP44	SOSTDC1
STX17	LOC101105047
MFSD2B	PLA2R1
LOC101108121	ELOVL5
FKBP1B	OLFML2B
TP53I3	C1H1orf226
SF3B6	LOC101118878
FAM228B	SYT16
PFN4	GRIP1
GDPD3	CAVIN3
MAPK3	ZNF786
TBX6	ZNF398
PPP4C	ZNF282
YPEL3	ZNF212
LOC105604728	ADCY5
ALDOA	QKI
TLCD3B	C9H8orf88
C24H16orf92	TMEM64
DOC2A	LOC114112857
INO80E	KNL1
RALGAPB	RAD51
ADIG	RMDN3
SLC32A1	GCHFR
HNRNPLL	DNAJC17
NMS	C7H15orf62
CHST10	SPTLC1
LONRF2	ZNF654
PCBP1	C1H3orf38
TRIM55	SEMA3A
CRH	GTF2IRD1
RAVER2	SMAD1
PLPPR5	THOC3
AKAP13	CNTNAP4
PTMA	MAD1L1
PDE6D	NOX4
GEMIN6	LOC105612090
LOC101114658	SOX21
HSPA5	ZNF462
RABEPK	CCNG1
PPP6C	NUDCD2
SCAI	HMMR

PEX7	RTN4RL1
SLC35D3	DPH1
LOC114110035	DAAM2
LOC101119087	MOCS1
SPTA1	LOC121819686
LOC101118753	ZNF638
LOC101119785	PRICKLE4
LOC101120044	FRS3
LOC101120294	USP49
LOC105612644	TOMM6
ADAMTS16	MED20
DGKG	LRRC8C
LOC114118401	SAMD12
PTGDR	VSTM2A
SLC24A2	MCU
METRNL	OIT3
NUP107	PLA2G12B
SLC35E3	C18H14orf180
RAP1B	TMEM179
DMBT1	FOXP4
LOC121817630	PNPLA1
LOC101111505	BNIP5
LOC101111242	KLHDC7A
C22H10orf120	LOC101102447
SELL	PRKCQ
SELE	NEK1
METTL18	CXCR4
C12H1orf112	TOR1AIP1
ZNF792	TTC7B
SPON1	SYK
LOC101114669	PHB
TUSC1	ZNF652
LOC101105523	KIF6
PDHX	ALB
SOGA1	LOC101103646
TLDC2	SLC35F1
SAMHD1	FGD3
RNF169	BICD2
LOC101102047	SYS1
LOC101109077	TP53TG5
UBXN2A	DBNDD2
FCRL5	PIGT
FCRL4	LOC105610219
E2F5	LOC101113455

RBIS	LOC101113720
LRRCC1	C3H2orf50
EDRF1	SLC66A3
UROS	ROCK2
LOC101116189	LIMD1
DHX32	LARS2
LOC106991782	ZBTB8B
LOC101106719	LOC114118753
USP19	CTNND2
QARS1	WNT8B
QRICH1	SEC31B
LOC121817252	TGM3
NDUFAF3	SLC8A1
DALRD3	PRKCB
IMPDH2	SASH1
VIRMA	LOC101108776
EEPD1	LOC101109041
IRF5	LOC101109300
TNPO3	LPXN
DDX10	GALK2
LOC114118004	COPS2
LOC101104027	ASCC3
CCN2	SELENOO
LOC101101868	LOC101114079
LOC114109594	LOC114110581
LOC114109612	LOC101102527
SNX13	PDZD9
RAB6A	LOC101115846
MRPL48	CDC7
LOC101104176	GRM5
CDH18	CNGB3
HTR4	CYTH1
HECW1	DNAH17
RCSD1	PACRG
MPZL1	TTYH1
RBBP8	LENG8
SPTLC3	CDC42EP5
PLPP1	LENG9
MTREX	LOC100192427
LOC101115571	MSH2
LOC101108675	GALNT1
LOC101116754	PITPNC1
CDK5RAP1	PSMD12
SNTA1	FAM76B

MOB1B	CEP57
DCK	LZTS1
NRXN1	FLRT1
LOC114109119	TCF4
CTLA4	IL15
GTF2H3	THSD7B
EIF2B1	FMNL2
DDX55	DSTYK
TMED2	TMCC2
RILPL1	MDN1
CAPRIN2	LYRM2
LOC101107776	ANKRD6
USP32	SLC25A15
LOC105616451	MRPS31
CA4	TRAPPC9
FAM83D	CDIN1
DHX35	POGK
LARS2	TADA1
TSPAN13	PLA2G4A
AGR2	MTRR
LOC114115867	KRT2
RBBP6	LOC101109951
LYG2	LOC101110219
MRPL30	KRT74
MITD1	KRT71
LIPT1	LMO1
LOC101111733	RIC3
IRF2BP2	ABT1
DNAJC13	HMGN4
ACP3	LOC105603811
ATRNL1	BTN1A1
LOC101102857	LOC101103021
VDR	UNC13B
WDR11	LOC105607745
LYG1	LOC105608645
HSPB8	DCDC1
SRRM4	CALCR
ADORA1	LOC101116738
CHI3L1	DENND2C
MYBPH	AMPD1
BTBD9	GUCY1A1
HIRIP3	VTI1A
TAOK2	CDH4
LOC105611202	MN1

LOC105615271	STUM
LOC114114955	TASP1
LOC105615366	UBE2V2
RFX3	LOC101118179
MOCS2	MAP3K19
ITGA2	CCNT2
FAM98A	ADAMTS18
LOC114116351	PLCXD2
ZNF804B	SPARCL1
MBNL2	SSTR1
ESCO1	CLEC14A
GREB1L	KIAA1328
BMP1	LOC121818515
SFTPC	PLPPR1
LGI3	CNTN3
REEP4	AMPH
HRURF	HIKESHI
HR	EED
NUDT18	MSC
FHIP2B	LOC114109004
CHST9	ZSCAN2
RAD54B	WDR73
LOC101102647	SEC11A
ARHGAP11A	NMB
CLPX	DPP6
LOC101119218	LPAR3
PARP16	MCOLN2
DSC1	ZNF536
DSC2	HPSE2
LOC101105127	SH3RF1
MSLN	MAST4
CHTF18	SPO11
GNG13	BMP7
RPUSD1	LCA5
LMF1	SH3BGRL2
MED16	LOC121819718
CFD	ZNF354C
R3HDM4	ZNF879
KISS1R	CLPSL2
ARID3A	LHFPL5
WDR18	CLPS
C3H2orf50	SRPK1
SLC66A3	APLF
ROCK2	FBXO48

NR3C2	PLEK
ARHGAP10	EPS8
MPPED1	SLC12A4
EFCAB6	PSMB10
PUM1	LCAT
ABCC2	LOC101110863
LOC101120033	DPEP3
C2H2orf76	DDX28
RPGRIP1	DPEP2NB
HNRNPC	DUS2
AHNAK2	RNASEH2B
CLBA1	PCSK6
GPR132	SNRPA1
LOC105605079	SORCS3
FAM149B1	LOC121819227
DNAJC9	WNK1
CFAP70	GTF2E1
MRPS16	PSTPIP2
BMP10	ATP5F1A
ARHGAP25	PCDH9
PIWIL2	AATF
POLR3D	ACACA
CBLB	LOC121820546
BSPRY	IL7R
WDR31	SPEF2
RNF183	EXOSC9
PRPF4	SMIM43
SEC24A	DPYSL5
CAMLG	CDK15
DDX46	COMMD1
CFAP36	CCT4
MYO1B	FAM161A
DIP2B	IL32
A4GALT	LOC105604626
LOC101115059	ZSCAN10
LOC101106962	ZNF205
ZSCAN4	LOC101120797
MATR3	ZNF213
LOC101122262	LOC121817913
FBXL13	VPS37C
LRRC17	LOC101122563
GSC	LOC114110083
PPP1R21	DGKH
FOXN2	LMBR1

COMMD8	NOM1
ATP10D	MNX1
RNF32	ME1
HDAC7	LOC101105912
DOCK9	AGL
FBXO22	MARCHF3
UBE2Q2	NFATC2
NRG4	ATP9A
RAB3GAP1	DYNC1H1
LOC114113193	LOC105601897
LOC101106246	HSP90AA1
TGFA	PLCB1
PLCB1	LOC105613810
SPZ1	ENY2
LOC101121850	NUDCD1
PANK3	LOC114117287
LOC121816921	PCGF5
SLC7A11	MYO1D
ERCC4	ARID5B
LOC121819707	NAA50
CHN2	ATP6V1A
LOC101104595	SYTL3
LOC105616741	EZR
YWHAZ	LOC121819838
GRIA4	DCLK3
GLI3	GOLGA4
PABPC1L	SP3
YWHAB	THSD4
TOMM34	MEMO1
STK4	B3GAT3
SOX18	EML3
TCEA2	ROM1
C13H20orf204	GANAB
PRPF6	C21H11orf98
SAMD10	INTS5
ZNF512B	CSKMT
UCKL1	LOC101112013
GPR85	LRRN4CL
TLK1	UBXN1
ACBD3	GNG3
LOC106991444	BSCL2
CACNA1D	TTC9C
PEMT	HNRNPUL2
RASD1	LOC101114319

MED9	KLHL3
NT5M	SCFD2
HIF1A	FIP1L1
ATXN7L1	DISC1
TEX14	RFTN2
RAD51C	LOC101109728
PPM1E	LOC101118373
LOC101102994	AK5
CDK8	ZZZ3
RBFOX2	SPSB4
SLC7A1	CCDC30
MTUS2	PPIH
SRBD1	YBX1
SAMD4A	NRG1
SRD5A3	FAM172A
TMEM165	LAPTM4B
ARMC12	RAD18
CLPSL2	OPTN
LHFPL5	SYBU
CLPS	EBAG9
SRPK1	FRMD4B
LOC101102848	CYFIP2
LOC101117235	FNDC9
ABI2	PCDH7
ICE2	RFTN1
ANXA2	DAZL
CLTA	PDE10A
GLIPR2	CDYL
CCIN	GRM3
CCDC39	MAML2
TTC14	MTMR2
DNER	CADM2
GCH1	EDIL3
WDHD1	CUBN
HERC3	LOC101111058
HERC5	LOC101122142
PYURF	LOC114109636
PIGY	NOTCH4
ARHGEF10L	GPSM3
SOCS4	PBX2
MAPK1IP1L	RNF5
NDUFAF6	AGER
PRKN	BANF2
LMBRD1	VIPR2

SULF1	IQGAP2
KCNA1	F2R
MIB1	ADI1
PCDH7	RNASEH1
JAK2	COLEC11
HOXA3	RPS7
HOXA2	ALLC
HOXA1	PLOD1
HOXA4	KIAA2013
UVRAG	GRIK1
LAMA3	URI1
POLQ	CDC42BPA
LOC105615576	CCDC88A
PITPNC1	FAM240A
PSMD12	TDGF1
PTGES3L	LRRC2
RUNDC1	CTNNA2
IFI35	STK32B
RPL27	CYTL1
VAT1	RSU1
RND2	TLE3
BRCA1	MAML3
OSBPL7	PREX2
TBX21	MASP1
NUP58	RTP1
MTMR6	GLG1
LOC106991529	RFWD3
FOXS1	OPCML
LOC101112411	ERAP2
TTLL9	LOC101102729
LOC101103002	STEAP4
PDRG1	EQTN
LIMS2	MOB3B
MYO7B	LOC114113024
GPR17	ZBTB7B
LOC101108561	AIG1
MRTFA	FBN1
LDB1	VAV3
PPRC1	DCP1A
NOLC1	TKT
TATDN2	PRKCD
IRAK2	LOC101109615
LYPD1	LOC106991189
GPR39	MGAT1

IL7	NR4A2
OTOG	CSMD2
CENPV	RFX7
PIGL	DAB1
NCOR1	CFAP20DC
ADAMTS17	GSG1L
DENND5B	LLPH
AMN1	MTMR6
ETFBKMT	SMO
NXPH2	AHCYL2
CAAP1	FOXP1
MTX2	OLFM3
MRE11	RYR1
ANKRD49	MAP4K1
AASDHPPT	EIF3K
TMEM192	LRRC72
LOC121820182	ANKMY2
REV3L	LDLRAD3
COG5	BSDC1
HBP1	ST6GAL1
FOXP1	LIMCH1
LOC101108033	DAPK1
LAPTM5	LOXL3
SAMM50	LOC101107432
PARVB	DOK1
CA12	LOC106990225
APH1B	SEMA4F
LOC101122084	FHIP1A
HES1	
CREG2	
RNF149	
CNOT11	
PLD5	
DCC	
SLC4A4	
IGF2BP2	
SENP2	
LIPH	
PTK2B	
VOPP1	
CTXND2	
SETDB1	
CERS2	
ANXA9	

FAM234A
RGS11
ARHGDIG
PDIA2
AXIN1
MRPL28
PGAP6
NME4
FAM186A
LIMA1
LOC101117532
NECTIN1
ATP6V1A
CCSER1
MINPP1
WDR19
RFC1
LARP4
BIN1
PACC1
PPP2R5A
LRP12
MYRF
TMEM258
FEN1
FADS1
LOC101119869
CLUL1
TYMS
ENOSF1
ADRB2
LRGUK
ARL15
GRID2
CAMK4
ZMYM4
LOC101109655
KIAA0319L
ARHGAP18
SPECC1
CNTNAP5
ADAM23
MCTP1
TCF7L2

ZYG11B
ZYG11A
LOC101116777
LOC105609512
SPSB4
PRG4
TPR
ODR4
GRIN3A
NOL10
DOCK4
LOC114118461
SIK1
SRD5A2
LOC114113944
HNRNPD
PRICKLE2
AP2A2
CHID1
TSPAN4
CD151
POLR2L
CRACR2B
ADGRB3
NGEF
LRP1B
UBB
SCGB3A2
C5H5orf46
ELAPOR1
SARS1
CELSR2
PSRC1
MYBPHL
VIT
AGTPBP1
BCHE
LOC105606568
RP9
NT5C3A
FKBP9
NRP1
ORC5
ZNF804A

MROH6
GSDMD
ZC3H3
LOC121820297
MAFA
PAAF1
GPR63
PTGER3
ZRANB2
BMP6
TEK
LOC121818663
EQTN
MOB3B
DSCAML1
IGFBP2
ZFP37
LOC101109939
LOC101110208
CHPT1
SYCP3
GNPTAB
LOC121820185
NTRK3
BICD1
GABRR3
LOC101104359
LOC114118388
LOC101103944
LOC101104875
LOC101105121
LOC101104620
DAP3
MSTO1
GON4L
LOC101110445
LOC101111392
LOC114118338
SLC35G2
CCDC179
LOC105604019
GAS2
KLF7
MYBPC1

ATP6V1C1
TMSB10
KCMF1
SERPINB11
SERPINB7
LOC121817741
SSBP2
DZIP3
ANKRD39
ANKRD23
CNNM3
CNNM4
LOC101110168
LOC101110440
SOS1
ARHGEF33
MORN2
ARFGEF1
DUSP5
NPAS3
MCHR2
TBC1D30
GNS
ACADSB
LOC101102859
HMX3
KIAA0895L
NOL3
EXOC3L1
ELMO3
E2F4
TMEM208
LRRC29
FHOD1
SLC9A5
PLEKHG4
KCTD19
MTMR9
LOC101118164
SULT6B1
EIF2AK2
GPATCH11
HEATR5B
ENO1

LOC114117228
FNDC3A
MLNR
CALCR
MCPH1
YBX2
SLC2A4
EIF5A
NEURL4
GPS2
LOC105607811
ACAP1
TMEM95
KCTD11
PLSCR3
TNK1
TMEM256
NLGN2
RAP1GDS1
ZC2HC1A
INSM2
ZBTB16
TC2N
FBLN5
SOST
DUSP3
CFAP97D1
GOLGA4
DIS3L2
PKN2
LOC101117028
LOC101117285
CCDC126
LOC106991101
LOC121819395
LOC101119648
LOC114115836
LOC101107872
LOC101119566
LOC101119825
LOC101120085
BCL2L1
TPX2
SLC23A1

PAIP2
MZB1
XDH
PARD3B
PMS1
FAM178B
SEMA4C
PIAS1
PKD1L3
LOC121816502
IST1
ZNF821
TBC1D15
TPH2
NEB
KDM7A
NAIF1
CNTN6
GORASP2
NEGR1
FAM185A
LOC101110703
LOC101111658
SUN2
DNAL4
NPTXR
CBX6
LOC114117623
LOC105606901
PHF20
HACE1
MAGI2
BARD1
TECPR2
ANKRD9
LRRC46
MRPL10
MCF2L2
LAMP3
PSTK
IKZF5
FAM172A
KIAA0825
STEAP3

TRAPPC9
SPRED2
ARHGEF28
AHR
ATP9A
SALL4
COP1
SMC3
DCDC1
MTRR
FASTKD3
C16H5orf49
ADCY2
CTNNA3
EDNRA
ATF7IP2
EMP2
ITGB8
LOC121817729
LOC105604529
LOC105604530
DPYSL3
C2H2orf72
PSMD1
HTR2B
TMEM183A
PPFIA4
HSPD1
LOC106990104
RFTN2
TTBK2
LOC114115593
TUT4
SLIT2
TXN2
FOXRED2
EIF3D
CACNG2
MSH4
RABGGTB
BMP4
BAIAP2L2
PLA2G6
CNTN5

TMC5
BPIFB1
DCTD
LOC101104591
CAV1
CAV2
TNS1
TF
LOC101117129
SRFBP1
NDUFB4
HGD
SLC25A21
SLCO3A1
EPHA6
NARS2
ITFG1
SLC50A1
EFNA1
LOC114113228
DPM3
TRIM46
KRTCAP2
MUC1
THBS3
MTX1
GBA
FAM189B
KIF5C
RUNX2
SUPT3H
AMER2
ST6GAL1
TTC19
ZSWIM7
COL15A1
LOC121817050
SIN3A
MAN2C1
NEIL1
SANBR
UBXN10
PLA2G2C
PLA2G2F

FANCF
LOC101102230
MALRD1
SMYD4
SERPINF1
RPA1
FMNL2
MAP2K3
SLC16A12
HOXA7
HOXA6
HOXA5
LOC101123268
CCDC148
UPP2
LOC106991226
SLITRK1
RNF213
LOC114117238
SSU72
LOC101105090
TMEM240
VWA1
ANKRD65
TMEM88B
MRPL20
CCNL2
LOC101107036
AURKAIP1
AAK1
NR2E1
PPM1H
MACROD2
KCTD9
GNRH1
DOCK5
ZDHHC17
TNS4
CCR7
DYRK4
RAD51AP1
C3H12orf4
EIF4G2
CTR9

LOC101122812
MXRA8
DVL1
C1H1orf185
SHC3
S1PR3
ZBED5
SNAPC1
IFIT3
LOC101102194
IFIT5
CERT1
PKP2
ELP4
PAX6
KDM5A
CCDC77
PTPN12
LOC101121244
LOC101114089
LOC443441
ASTN2
APCDD1L
VAPB
RAB22A
GPM6A
SEMA3C
RNF111
SLTM
CPNE2
PSME3IP1
RSPRY1
NR6A1
CHIA
PIFO
PDE7B
NSMCE2
WASHC5
PRSS12
NDST3
RIMS2
LOC105616742
LOC105606886
TMPRSS15

CHODL
RUNX1
LOC101118932
DLGAP4
MYL9
FXYD2
RPRD1A
C23H18orf21
GREM1
LOC101111905
MBD1
CXXC1
CDK19
RPP30
HTR7
SHQ1
CSMD1
F13A1
KLB
LOC101102109
LOC101117398
IPO4
REC8
FNDC7
STXBP3
AKNAD1
NOX4
TYR
ERC2
RAC2
CYTH4
COLGALT2
PTPRF
KDM4A
LOC101111247
MYO16
FOXO3
ACTN4
CAPN12
LGALS7
LOC101112249
LGALS4
SLC22A3
TSEN2

PPARG
NME9
ARMC8
HNF4G
FAM78B
CPA6
NMRK2
ATCAY
ZFR2
UBASH3B
CRTAM
OBI1
ESRRG
MYOG
CCDC178
PIK3C2A
PSMG4
TUBB2B
LOC101122734
BPHL
RIPK1
DGKB
EPDR1
TRIM14
NANS
ANP32B
FBN2
C6
SS18L1
PSMA7
LSM14B
TAF4
FOXJ2
C3AR1
NECAP1
CLEC4A
NTM
VWA8
LAMA2
KALRN
ANO4
CEPT1
DENND2D
CHI3L2

KCNMB4
CNOT2
FGF12
USP34
AHSA2P
CDK14
KCNK2
RABL3
GTF2E1
SCAF11
SHC4
EID1
CEP152
TOGARAM2
PCARE
CLIP4
APC
LOC106991169
LOC105615370
RASSF2
MRPL46
MRPS11
DET1
C12H1orf174
DFFB
COQ8A
CDC42BPA
GPR68
TNIK
GHR
CCDC88C
DNAJB13
UCP2
NCOA1
ARHGAP24
COL9A1
BABAM2
FAM102A
KIF6
RNASEH2B
LOC101106088
DYNLRB2
CDYL2
SLC12A2

ABCG2
PKD2
CPM
B4GALNT1
LOC101119634
ARHGEF25
PIP4K2C
DTX3
KIF5A
DCTN2
DCAF6
MAP3K10
TTC9B
CCNP
AKT2
SLC45A4
DENND3
CCDC170
INO80D
LOC121818517
NDUFS1
LOC101108695
GABBR2
BCAP29
DUS4L
SLC26A4
CSRP2
FSTL5
ZIC4
CCDC136
FLNC
LOC101105154
ATP6V1F
ATP6V1FNB
PIEZO2
CDK5R2
FEV
GUCY1A2
HOXA10
HOXA9
LOC114118402
RPS13
DRG1
EIF4ENIF1

SFI1
BCAT1
NUDT12
ECH1
ASTN1
PPARGC1B
ATP6V0A2
TCTN2
TRUB2
SWI5
GOLGA2
HNRNPL
BASP1
HIKESHI
EED
UBE2O
SPHK1
PRPSAP1
QRICH2
SEC22A
ADCY5
TMEM250
NACC2
METTL15
DACH1
PTK2
AGO2
ORC2
FAM126B
EBF2
MYO5B
DNAH10
OTUD7A
FMN1
C14H16orf74
GINS2
EMC8
LOC101105179
CXCL12
ALKBH6
SYNE4
SDHAF1
CLIP3
THAP8

WDR62
KCTD17
TMPRSS6
IL2RB
ANKRD55
LOC101108001
TREM2
TREML1
TREML2
NFYA
CCL25
FBN3
PAX2
SLF2
PCSK1
ESR1
SYNE1
ATP10A
WDR1
CLEC4D
CLEC4E
VPS37C
LOC101122563
LOC114110083
PPP2R2B
BAALC
LOC105611918
SOX8
LOC101123171
LOC101108715
ABCC5
BEST3
STMND1
FADS2
EIF4G3
CPE
TSPAN5
PRTFDC1
ENKUR
MACC1
ZHX1
C9H8orf76
FAM83A
MBD6

CDH3
CDH1
LOC101115135
SHISA2
LOC101115632
LOC121819185
C1QTNF6
CCDC32
RPUSD2
KNL1
DDIT3
MARS1
CKS2
SECISBP2
SEMA4D
ST8SIA4
CHST14
ACVR2A
PIK3CG
CDKAL1
LOC101113889
ABHD17C
TMF1
EOGT
LOC114117588
GALNT10
TRIM8
ARL3
SFXN2
WBP1L
LOC101115843
AP1G2
THTPA
ZFHX2
NGDN
HDAC3
DIAPH1
RELL2
FCHSD1
ARAP3
PAN3
ADK
LTO1
FGF19

FGF4
LOC101122312
PTPN23
LOC101122819
KLHL18
LOC101111631
SUCLG2
TFPI
LOC101108428
ITIH4
MUSTN1
ITIH3
ITIH1
GMDS
FOXF2
FOXC1
PSMB7
NEK6
CPLANE1
C1QL2
FHOD3
GABRG2
IL23R
IL12RB2
IVD
BAHD1
LOC101110090
LOC101109601
CEP72
NCOR2
LOC101115905
LOC114115882
PLEKHG3
SPTB
LLPH
MAP2K4
LOC101114791
NNT
PAIP1
TGFBR1
ADAMTS7
SIAH1
LOC114117844
ORC4

TMBIM4
EEF1G
TUT1
EML3
MTA2
B3GAT3
ROM1
GANAB
C21H11orf98
INTS5
CSKMT
LOC101112013
LRRN4CL
UBXN1
BSCL2
