# Supplementary material for: Genetic Structure and Selection Signals for Extreme Environment Adaptation in Lop Sheep of Xinjiang
Source: Biology (Basel). 2025 Mar 25;14(4):337. doi: 10.3390/biology14040337 (PMC12025199; doi:10.3390/biology14040337)
Supplement: Supplementary file 1 [file biology-14-00337-s001.zip › Supplementary Table S6.pdf]

GO

ONTOLOGY	ID	Description	GeneRatio	BgRatio	pvalue	p.adjust	qvalue	geneID	Count
BP	GO:0006479	protein methylation	3/33	26/4831	0.000679	0.162171	0.162171	RAB6A/EED/CSKMT	3
BP	GO:0008213	protein alkylation	3/33	26/4831	0.000679	0.162171	0.162171	RAB6A/EED/CSKMT	3
BP	GO:0016482	cytosolic transport	3/33	30/4831	0.00104	0.165711	0.165711	VTI1A/RAB6A/DNAJC13	3
BP	GO:0032259	methylation	4/33	89/4831	0.002929	0.34996	0.34996	RAB6A/EED/METTL15/CSKMT	4
BP	GO:0043414	macromolecule methylation	3/33	49/4831	0.004321	0.407499	0.407499	RAB6A/EED/CSKMT	3
BP	GO:0042147	retrograde transport, endos	2/33	16/4831	0.005115	0.407499	0.407499	VTI1A/RAB6A	2
BP	GO:1903305	regulation of regulated sec	2/33	18/4831	0.006466	0.441555	0.441555	RAP1B/STXBP3	2
BP	GO:0051048	negative regulation of secr	2/33	21/4831	0.008763	0.45878	0.45878	RAP1B/STXBP3	2
BP	GO:1903531	negative regulation of secr	2/33	21/4831	0.008763	0.45878	0.45878	RAP1B/STXBP3	2
BP	GO:0007030	Golgi organization	2/33	22/4831	0.009598	0.45878	0.45878	VTI1A/HIKESHI	2
BP	GO:0010256	endomembrane system organiz	3/33	77/4831	0.015071	0.529274	0.529274	VTI1A/HIKESHI/DNAJC13	3
BP	GO:0060627	regulation of vesicle-media	3/33	78/4831	0.015602	0.529274	0.529274	RAP1B/DNAJC13/STXBP3	3
BP	GO:0017157	regulation of exocytosis	2/33	32/4831	0.019751	0.529274	0.529274	RAP1B/STXBP3	2
BP	GO:0016197	endosomal transport	2/33	35/4831	0.023395	0.529274	0.529274	VTI1A/RAB6A	2
BP	GO:0030323	respiratory tube developmen	2/33	36/4831	0.024666	0.529274	0.529274	FOXP1/HIKESHI	2
BP	GO:0030324	lung development	2/33	36/4831	0.024666	0.529274	0.529274	FOXP1/HIKESHI	2
BP	GO:0045055	regulated exocytosis	2/33	41/4831	0.031434	0.529274	0.529274	RAP1B/STXBP3	2
BP	GO:0060541	respiratory system developm	2/33	41/4831	0.031434	0.529274	0.529274	FOXP1/HIKESHI	2
BP	GO:0007596	blood coagulation	2/33	42/4831	0.032866	0.529274	0.529274	F13A1/STXBP3	2
BP	GO:0007599	hemostasis	2/33	42/4831	0.032866	0.529274	0.529274	F13A1/STXBP3	2
BP	GO:0050817	coagulation	2/33	44/4831	0.035807	0.529274	0.529274	F13A1/STXBP3	2
BP	GO:0002366	leukocyte activation involv	2/33	49/4831	0.043585	0.529274	0.529274	FOXP1/STXBP3	2
BP	GO:0002263	cell activation involved in	2/33	50/4831	0.045211	0.529274	0.529274	FOXP1/STXBP3	2
BP	GO:0016192	vesicle-mediated transport	5/33	311/4831	0.057582	0.529274	0.529274	VTI1A/RAB6A/RAP1B/DNAJC13/STXBP3	5
BP	GO:0010463	mesenchymal cell proliferat	1/33	10/4831	0.066307	0.529274	0.529274	FOXP1	1
BP	GO:0016073	snRNA metabolic process	1/33	10/4831	0.066307	0.529274	0.529274	INTS5	1
BP	GO:0022406	membrane docking	1/33	10/4831	0.066307	0.529274	0.529274	STXBP3	1
BP	GO:0046324	regulation of glucose impor	1/33	10/4831	0.066307	0.529274	0.529274	STXBP3	1
BP	GO:0050805	negative regulation of syna	1/33	10/4831	0.066307	0.529274	0.529274	RAP1B	1
BP	GO:0051591	response to cAMP	1/33	10/4831	0.066307	0.529274	0.529274	RAP1B	1
BP	GO:1990868	response to chemokine	1/33	10/4831	0.066307	0.529274	0.529274	SLC12A2	1
BP	GO:1990869	cellular response to chemok	1/33	10/4831	0.066307	0.529274	0.529274	SLC12A2	1
BP	GO:0051649	establishment of localizati	6/33	437/4831	0.072127	0.529274	0.529274	VTI1A/RAB6A/RAP1B/HIKESHI/DNAJC13	6

BP	GO:0000381	regulation of alternative m	1/33	11/4831	0.072698	0.529274	0.529274	FAM172A	1
BP	GO:0006890	retrograde vesicle-mediated	1/33	11/4831	0.072698	0.529274	0.529274	RAB6A	1
BP	GO:0034067	protein localization to Gol	1/33	11/4831	0.072698	0.529274	0.529274	RAB6A	1
BP	GO:0036230	granulocyte activation	1/33	11/4831	0.072698	0.529274	0.529274	STXBP3	1
BP	GO:0042093	T-helper cell differentiati	1/33	11/4831	0.072698	0.529274	0.529274	FOXP1	1
BP	GO:0055017	cardiac muscle tissue growt	1/33	11/4831	0.072698	0.529274	0.529274	FOXP1	1
BP	GO:0060038	cardiac muscle cell prolife	1/33	11/4831	0.072698	0.529274	0.529274	FOXP1	1
BP	GO:0060419	heart growth	1/33	11/4831	0.072698	0.529274	0.529274	FOXP1	1
BP	GO:0061028	establishment of endothelia	1/33	11/4831	0.072698	0.529274	0.529274	RAP1B	1
BP	GO:0070527	platelet aggregation	1/33	11/4831	0.072698	0.529274	0.529274	STXBP3	1
BP	GO:1901998	toxin transport	1/33	11/4831	0.072698	0.529274	0.529274	DNAJC17	1
BP	GO:0000380	alternative mRNA splicing,	1/33	12/4831	0.079047	0.529274	0.529274	FAM172A	1
BP	GO:0002287	alpha-beta T cell activatio	1/33	12/4831	0.079047	0.529274	0.529274	FOXP1	1
BP	GO:0002293	alpha-beta T cell different	1/33	12/4831	0.079047	0.529274	0.529274	FOXP1	1
BP	GO:0002294	CD4-positive, alpha-beta T	1/33	12/4831	0.079047	0.529274	0.529274	FOXP1	1
BP	GO:0002444	myeloid leukocyte mediated	1/33	12/4831	0.079047	0.529274	0.529274	STXBP3	1
BP	GO:0010827	regulation of glucose trans	1/33	12/4831	0.079047	0.529274	0.529274	STXBP3	1
BP	GO:0034205	amyloid-beta formation	1/33	12/4831	0.079047	0.529274	0.529274	APH1B	1
BP	GO:0043299	leukocyte degranulation	1/33	12/4831	0.079047	0.529274	0.529274	STXBP3	1
BP	GO:0045214	sarcomere organization	1/33	12/4831	0.079047	0.529274	0.529274	FOXP1	1
BP	GO:0046928	regulation of neurotransmit	1/33	12/4831	0.079047	0.529274	0.529274	RAP1B	1
BP	GO:0051588	regulation of neurotransmit	1/33	12/4831	0.079047	0.529274	0.529274	RAP1B	1
BP	GO:0098900	regulation of action potent	1/33	12/4831	0.079047	0.529274	0.529274	FOXP1	1
BP	GO:0006486	protein glycosylation	2/33	71/4831	0.083992	0.529274	0.529274	ST6GAL1/B3GAT3	2
BP	GO:0006887	exocytosis	2/33	71/4831	0.083992	0.529274	0.529274	RAP1B/STXBP3	2
BP	GO:0043413	macromolecule glycosylation	2/33	71/4831	0.083992	0.529274	0.529274	ST6GAL1/B3GAT3	2
BP	GO:0002200	somatic diversification of	1/33	13/4831	0.085353	0.529274	0.529274	FOXP1	1
BP	GO:0002562	somatic diversification of	1/33	13/4831	0.085353	0.529274	0.529274	FOXP1	1
BP	GO:0016444	somatic cell DNA recombinat	1/33	13/4831	0.085353	0.529274	0.529274	FOXP1	1
BP	GO:0046323	glucose import	1/33	13/4831	0.085353	0.529274	0.529274	STXBP3	1
BP	GO:0046620	regulation of organ growth	1/33	13/4831	0.085353	0.529274	0.529274	FOXP1	1
BP	GO:0098659	inorganic cation import acr	1/33	13/4831	0.085353	0.529274	0.529274	SLC12A2	1
BP	GO:0099587	inorganic ion import across	1/33	13/4831	0.085353	0.529274	0.529274	SLC12A2	1
BP	GO:0070085	glycosylation	2/33	73/4831	0.088088	0.529274	0.529274	ST6GAL1/B3GAT3	2
BP	GO:0002067	glandular epithelial cell d	1/33	14/4831	0.091618	0.529274	0.529274	FOXP1	1

BP	GO:0002275	myeloid cell activation inv	1/33	14/4831	0.091618	0.529274	0.529274	STXBP3	1
BP	GO:0002292	T cell differentiation invo	1/33	14/4831	0.091618	0.529274	0.529274	FOXP1	1
BP	GO:0006040	amino sugar metabolic proce	1/33	14/4831	0.091618	0.529274	0.529274	ST6GAL1	1
BP	GO:0014855	striated muscle cell prolif	1/33	14/4831	0.091618	0.529274	0.529274	FOXP1	1
BP	GO:0046683	response to organophosphoru	1/33	14/4831	0.091618	0.529274	0.529274	RAP1B	1
BP	GO:0048247	lymphocyte chemotaxis	1/33	14/4831	0.091618	0.529274	0.529274	SLC12A2	1
BP	GO:0050435	amyloid-beta metabolic proc	1/33	14/4831	0.091618	0.529274	0.529274	APH1B	1
BP	GO:0001885	endothelial cell developmen	1/33	15/4831	0.097841	0.529274	0.529274	RAP1B	1
BP	GO:0002639	positive regulation of immu	1/33	15/4831	0.097841	0.529274	0.529274	FOXP1	1
BP	GO:0009408	response to heat	1/33	15/4831	0.097841	0.529274	0.529274	HIKESHI	1
BP	GO:0021510	spinal cord development	1/33	15/4831	0.097841	0.529274	0.529274	FOXP1	1
BP	GO:0034109	homotypic cell-cell adhesio	1/33	15/4831	0.097841	0.529274	0.529274	STXBP3	1
BP	GO:0042987	amyloid precursor protein c	1/33	15/4831	0.097841	0.529274	0.529274	APH1B	1
BP	GO:0055007	cardiac muscle cell differe	1/33	15/4831	0.097841	0.529274	0.529274	FOXP1	1
BP	GO:0072678	T cell migration	1/33	15/4831	0.097841	0.529274	0.529274	SLC12A2	1
BP	GO:0050878	regulation of body fluid le	2/33	78/4831	0.098586	0.529274	0.529274	F13A1/STXBP3	2
BP	GO:0051051	negative regulation of tran	2/33	78/4831	0.098586	0.529274	0.529274	RAP1B/STXBP3	2
BP	GO:0043367	CD4-positive, alpha-beta T	1/33	16/4831	0.104023	0.529274	0.529274	FOXP1	1
BP	GO:0008645	hexose transmembrane transp	1/33	17/4831	0.110163	0.529274	0.529274	STXBP3	1
BP	GO:0014074	response to purine-containi	1/33	17/4831	0.110163	0.529274	0.529274	RAP1B	1
BP	GO:0015749	monosaccharide transmembran	1/33	17/4831	0.110163	0.529274	0.529274	STXBP3	1
BP	GO:0016079	synaptic vesicle exocytosis	1/33	17/4831	0.110163	0.529274	0.529274	RAP1B	1
BP	GO:0018022	peptidyl-lysine methylation	1/33	17/4831	0.110163	0.529274	0.529274	CSKMT	1
BP	GO:0032731	positive regulation of inte	1/33	17/4831	0.110163	0.529274	0.529274	PANX1	1
BP	GO:1904659	glucose transmembrane trans	1/33	17/4831	0.110163	0.529274	0.529274	STXBP3	1
BP	GO:0009101	glycoprotein biosynthetic p	2/33	85/4831	0.113845	0.529274	0.529274	ST6GAL1/B3GAT3	2
BP	GO:0042060	wound healing	2/33	86/4831	0.116074	0.529274	0.529274	F13A1/STXBP3	2
BP	GO:0140694	non-membrane-bounded organe	2/33	86/4831	0.116074	0.529274	0.529274	FOXP1/BSCL2	2
BP	GO:0030239	myofibril assembly	1/33	18/4831	0.116263	0.529274	0.529274	FOXP1	1
BP	GO:0034219	carbohydrate transmembrane	1/33	18/4831	0.116263	0.529274	0.529274	STXBP3	1
BP	GO:0035051	cardiocyte differentiation	1/33	18/4831	0.116263	0.529274	0.529274	FOXP1	1
BP	GO:0042982	amyloid precursor protein m	1/33	18/4831	0.116263	0.529274	0.529274	APH1B	1
BP	GO:0055002	striated muscle cell develo	1/33	18/4831	0.116263	0.529274	0.529274	FOXP1	1
BP	GO:0002637	regulation of immunoglobuli	1/33	19/4831	0.122323	0.529274	0.529274	FOXP1	1
BP	GO:0016571	histone methylation	1/33	19/4831	0.122323	0.529274	0.529274	EED	1

BP	GO:0048024	regulation of mRNA splicing	1/33	19/4831	0.122323	0.529274	0.529274	FAM172A	1
BP	GO:0061640	cytoskeleton-dependent cyto	1/33	19/4831	0.122323	0.529274	0.529274	DCTN3	1
BP	GO:0002286	T cell activation involved	1/33	20/4831	0.128342	0.529274	0.529274	FOXP1	1
BP	GO:0006418	tRNA aminoacylation for pro	1/33	20/4831	0.128342	0.529274	0.529274	LARS2	1
BP	GO:0006487	protein N-linked glycosylat	1/33	20/4831	0.128342	0.529274	0.529274	ST6GAL1	1
BP	GO:0019915	lipid storage	1/33	20/4831	0.128342	0.529274	0.529274	BSCL2	1
BP	GO:0030168	platelet activation	1/33	20/4831	0.128342	0.529274	0.529274	STXBP3	1
BP	GO:0043039	tRNA aminoacylation	1/33	20/4831	0.128342	0.529274	0.529274	LARS2	1
BP	GO:0023061	signal release	2/33	94/4831	0.134299	0.529274	0.529274	RAP1B/STXBP3	2
BP	GO:0002377	immunoglobulin production	1/33	21/4831	0.134321	0.529274	0.529274	FOXP1	1
BP	GO:0007269	neurotransmitter secretion	1/33	21/4831	0.134321	0.529274	0.529274	RAP1B	1
BP	GO:0032732	positive regulation of inte	1/33	21/4831	0.134321	0.529274	0.529274	PANX1	1
BP	GO:0043038	amino acid activation	1/33	21/4831	0.134321	0.529274	0.529274	LARS2	1
BP	GO:0046632	alpha-beta T cell different	1/33	21/4831	0.134321	0.529274	0.529274	FOXP1	1
BP	GO:0071346	cellular response to interf	1/33	21/4831	0.134321	0.529274	0.529274	STXBP3	1
BP	GO:0099643	signal release from synapse	1/33	21/4831	0.134321	0.529274	0.529274	RAP1B	1
BP	GO:0009100	glycoprotein metabolic proc	2/33	95/4831	0.136623	0.529274	0.529274	ST6GAL1/B3GAT3	2
BP	GO:0001508	action potential	1/33	22/4831	0.14026	0.529274	0.529274	FOXP1	1
BP	GO:0006879	cellular iron ion homeostas	1/33	22/4831	0.14026	0.529274	0.529274	ATP6V1A	1
BP	GO:0035710	CD4-positive, alpha-beta T	1/33	22/4831	0.14026	0.529274	0.529274	FOXP1	1
BP	GO:0043484	regulation of RNA splicing	1/33	22/4831	0.14026	0.529274	0.529274	FAM172A	1
BP	GO:0045446	endothelial cell differenti	1/33	22/4831	0.14026	0.529274	0.529274	RAP1B	1
BP	GO:0071453	cellular response to oxygen	1/33	22/4831	0.14026	0.529274	0.529274	ATP6V1A	1
BP	GO:0002065	columnar/cuboidal epithelia	1/33	23/4831	0.146159	0.529274	0.529274	FOXP1	1
BP	GO:0008643	carbohydrate transport	1/33	23/4831	0.146159	0.529274	0.529274	STXBP3	1
BP	GO:0010927	cellular component assembly	1/33	23/4831	0.146159	0.529274	0.529274	FOXP1	1
BP	GO:0032611	interleukin-1 beta producti	1/33	23/4831	0.146159	0.529274	0.529274	PANX1	1
BP	GO:0032651	regulation of interleukin-1	1/33	23/4831	0.146159	0.529274	0.529274	PANX1	1
BP	GO:0034763	negative regulation of tran	1/33	23/4831	0.146159	0.529274	0.529274	STXBP3	1
BP	GO:0009266	response to temperature sti	1/33	24/4831	0.15202	0.538938	0.538938	HIKESHI	1
BP	GO:0030856	regulation of epithelial ce	1/33	24/4831	0.15202	0.538938	0.538938	FOXP1	1
BP	GO:0009611	response to wounding	2/33	103/4831	0.15553	0.538938	0.538938	F13A1/STXBP3	2
BP	GO:0007586	digestion	1/33	25/4831	0.157841	0.538938	0.538938	CLPS	1
BP	GO:0034341	response to interferon-gamm	1/33	25/4831	0.157841	0.538938	0.538938	STXBP3	1
BP	GO:0072676	lymphocyte migration	1/33	25/4831	0.157841	0.538938	0.538938	SLC12A2	1

BP	GO:0099504	synaptic vesicle cycle	1/33	25/4831	0.157841	0.538938	0.538938	RAP1B	1
BP	GO:0018193	peptidyl-amino acid modific	3/33	202/4831	0.157848	0.538938	0.538938	RAB6A/ST6GAL1/CSKMT	3
BP	GO:0003158	endothelium development	1/33	26/4831	0.163624	0.546938	0.546938	RAP1B	1
BP	GO:0030010	establishment of cell polar	1/33	26/4831	0.163624	0.546938	0.546938	RAP1B	1
BP	GO:0050905	neuromuscular process	1/33	26/4831	0.163624	0.546938	0.546938	VTI1A	1
BP	GO:1903530	regulation of secretion by	2/33	108/4831	0.167602	0.547012	0.547012	RAP1B/STXBP3	2
BP	GO:0030183	B cell differentiation	1/33	27/4831	0.169368	0.547012	0.547012	FOXP1	1
BP	GO:0050684	regulation of mRNA processi	1/33	27/4831	0.169368	0.547012	0.547012	FAM172A	1
BP	GO:0055072	iron ion homeostasis	1/33	27/4831	0.169368	0.547012	0.547012	ATP6V1A	1
BP	GO:0099003	vesicle-mediated transport	1/33	27/4831	0.169368	0.547012	0.547012	RAP1B	1
BP	GO:0035265	organ growth	1/33	28/4831	0.175074	0.557383	0.557383	FOXP1	1
BP	GO:0098739	import across plasma membra	1/33	28/4831	0.175074	0.557383	0.557383	SLC12A2	1
BP	GO:0001505	regulation of neurotransmit	1/33	29/4831	0.180742	0.557383	0.557383	RAP1B	1
BP	GO:0001678	cellular glucose homeostasi	1/33	29/4831	0.180742	0.557383	0.557383	STXBP3	1
BP	GO:0006836	neurotransmitter transport	1/33	29/4831	0.180742	0.557383	0.557383	RAP1B	1
BP	GO:0032612	interleukin-1 production	1/33	29/4831	0.180742	0.557383	0.557383	PANX1	1
BP	GO:0032652	regulation of interleukin-1	1/33	29/4831	0.180742	0.557383	0.557383	PANX1	1
BP	GO:0001819	positive regulation of cyto	2/33	114/4831	0.182303	0.558596	0.558596	PANX1/FOXP1	2
BP	GO:0046907	intracellular transport	4/33	329/4831	0.183969	0.56011	0.56011	VTI1A/RAB6A/HIKESHI/DNAJC13	4
BP	GO:0002252	immune effector process	2/33	116/4831	0.18725	0.56649	0.56649	FOXP1/STXBP3	2
BP	GO:0006935	chemotaxis	2/33	117/4831	0.189731	0.566821	0.566821	FOXP1/SLC12A2	2
BP	GO:0051046	regulation of secretion	2/33	117/4831	0.189731	0.566821	0.566821	RAP1B/STXBP3	2
BP	GO:0002702	positive regulation of prod	1/33	31/4831	0.191964	0.567158	0.567158	FOXP1	1
BP	GO:0042330	taxis	2/33	118/4831	0.192217	0.567158	0.567158	FOXP1/SLC12A2	2
BP	GO:0000910	cytokinesis	1/33	33/4831	0.203038	0.582555	0.582555	DCTN3	1
BP	GO:0006814	sodium ion transport	1/33	33/4831	0.203038	0.582555	0.582555	SLC12A2	1
BP	GO:0007411	axon guidance	1/33	33/4831	0.203038	0.582555	0.582555	FOXP1	1
BP	GO:0097485	neuron projection guidance	1/33	33/4831	0.203038	0.582555	0.582555	FOXP1	1
BP	GO:0007163	establishment or maintenanc	1/33	34/4831	0.208519	0.582555	0.582555	RAP1B	1
BP	GO:0046631	alpha-beta T cell activatio	1/33	34/4831	0.208519	0.582555	0.582555	FOXP1	1
BP	GO:0048738	cardiac muscle tissue devel	1/33	34/4831	0.208519	0.582555	0.582555	FOXP1	1
BP	GO:0098657	import into cell	1/33	34/4831	0.208519	0.582555	0.582555	SLC12A2	1
BP	GO:1901888	regulation of cell junction	1/33	34/4831	0.208519	0.582555	0.582555	RAP1B	1
BP	GO:0006821	chloride transport	1/33	35/4831	0.213964	0.582555	0.582555	SLC12A2	1
BP	GO:0030073	insulin secretion	1/33	35/4831	0.213964	0.582555	0.582555	STXBP3	1

BP	GO:0055001	muscle cell development	1/33	35/4831	0.213964	0.582555	0.582555	FOXP1	1
BP	GO:0098742	cell-cell adhesion via plas	1/33	35/4831	0.213964	0.582555	0.582555	LGALS7	1
BP	GO:0002285	lymphocyte activation invol	1/33	36/4831	0.219372	0.582555	0.582555	FOXP1	1
BP	GO:0006606	protein import into nucleus	1/33	36/4831	0.219372	0.582555	0.582555	HIKESHI	1
BP	GO:0046916	cellular transition metal i	1/33	36/4831	0.219372	0.582555	0.582555	ATP6V1A	1
BP	GO:0051170	import into nucleus	1/33	36/4831	0.219372	0.582555	0.582555	HIKESHI	1
BP	GO:0070374	positive regulation of ERK1	1/33	36/4831	0.219372	0.582555	0.582555	RAP1B	1
BP	GO:0007219	Notch signaling pathway	1/33	37/4831	0.224745	0.590264	0.590264	APH1B	1
BP	GO:0014706	striated muscle tissue deve	1/33	37/4831	0.224745	0.590264	0.590264	FOXP1	1
BP	GO:0031334	positive regulation of prot	1/33	38/4831	0.230081	0.600977	0.600977	RAP1B	1
BP	GO:0002700	regulation of production of	1/33	39/4831	0.235382	0.608177	0.608177	FOXP1	1
BP	GO:0007519	skeletal muscle tissue deve	1/33	39/4831	0.235382	0.608177	0.608177	FOXP1	1
BP	GO:0030855	epithelial cell differentia	2/33	137/4831	0.240163	0.615339	0.615339	FOXP1/RAP1B	2
BP	GO:0030036	actin cytoskeleton organiza	2/33	139/4831	0.245265	0.615339	0.615339	CDC42BPA/FOXP1	2
BP	GO:0002440	production of molecular med	1/33	41/4831	0.245878	0.615339	0.615339	FOXP1	1
BP	GO:0019882	antigen processing and pres	1/33	41/4831	0.245878	0.615339	0.615339	RAB6A	1
BP	GO:0031032	actomyosin structure organi	1/33	41/4831	0.245878	0.615339	0.615339	FOXP1	1
BP	GO:0060538	skeletal muscle organ devel	1/33	41/4831	0.245878	0.615339	0.615339	FOXP1	1
BP	GO:0070482	response to oxygen levels	1/33	42/4831	0.251073	0.625068	0.625068	ATP6V1A	1
BP	GO:0055076	transition metal ion homeos	1/33	43/4831	0.256234	0.630961	0.630961	ATP6V1A	1
BP	GO:0098609	cell-cell adhesion	2/33	144/4831	0.258045	0.630961	0.630961	LGALS7/STXBP3	2
BP	GO:0015698	inorganic anion transport	1/33	44/4831	0.26136	0.630961	0.630961	SLC12A2	1
BP	GO:0032868	response to insulin	1/33	44/4831	0.26136	0.630961	0.630961	STXBP3	1
BP	GO:0033002	muscle cell proliferation	1/33	44/4831	0.26136	0.630961	0.630961	FOXP1	1
BP	GO:0050679	positive regulation of epit	1/33	44/4831	0.26136	0.630961	0.630961	FOXP1	1
BP	GO:0016050	vesicle organization	1/33	45/4831	0.266452	0.636821	0.636821	DNAJC13	1
BP	GO:0071674	mononuclear cell migration	1/33	45/4831	0.266452	0.636821	0.636821	SLC12A2	1
BP	GO:0034660	ncRNA metabolic process	2/33	148/4831	0.268284	0.638009	0.638009	LARS2/INTS5	2
BP	GO:0097435	supramolecular fiber organi	2/33	150/4831	0.273407	0.643175	0.643175	FOXP1/FBLN5	2
BP	GO:0030029	actin filament-based proces	2/33	151/4831	0.275968	0.643175	0.643175	CDC42BPA/FOXP1	2
BP	GO:0002274	myeloid leukocyte activatio	1/33	47/4831	0.276534	0.643175	0.643175	STXBP3	1
BP	GO:0017038	protein import	1/33	47/4831	0.276534	0.643175	0.643175	HIKESHI	1
BP	GO:0007417	central nervous system deve	2/33	152/4831	0.27853	0.643175	0.643175	FOXP1/STXBP3	2
BP	GO:0010243	response to organonitrogen	2/33	152/4831	0.27853	0.643175	0.643175	RAP1B/STXBP3	2
BP	GO:0030072	peptide hormone secretion	1/33	48/4831	0.281525	0.643772	0.643772	STXBP3	1

BP	GO:0002064	epithelial cell development	1/33	49/4831	0.286482	0.643772	0.643772	RAP1B	1
BP	GO:0002790	peptide secretion	1/33	49/4831	0.286482	0.643772	0.643772	STXBP3	1
BP	GO:0071345	cellular response to cytoki	2/33	157/4831	0.291333	0.643772	0.643772	STXBP3/SLC12A2	2
BP	GO:0016485	protein processing	1/33	50/4831	0.291406	0.643772	0.643772	APH1B	1
BP	GO:0032386	regulation of intracellular	1/33	50/4831	0.291406	0.643772	0.643772	DNAJC13	1
BP	GO:0050804	modulation of chemical syna	1/33	50/4831	0.291406	0.643772	0.643772	RAP1B	1
BP	GO:0051146	striated muscle cell differ	1/33	50/4831	0.291406	0.643772	0.643772	FOXP1	1
BP	GO:0099177	regulation of trans-synapti	1/33	50/4831	0.291406	0.643772	0.643772	RAP1B	1
BP	GO:0007010	cytoskeleton organization	3/33	277/4831	0.293049	0.643772	0.643772	CDC42BPA/FOXP1/CEP350	3
BP	GO:0030198	extracellular matrix organi	1/33	51/4831	0.296297	0.643772	0.643772	FBLN5	1
BP	GO:0043062	extracellular structure org	1/33	51/4831	0.296297	0.643772	0.643772	FBLN5	1
BP	GO:0045229	external encapsulating stru	1/33	51/4831	0.296297	0.643772	0.643772	FBLN5	1
BP	GO:0002699	positive regulation of immu	1/33	52/4831	0.301155	0.648627	0.648627	FOXP1	1
BP	GO:0055082	cellular chemical homeostas	2/33	162/4831	0.304119	0.648627	0.648627	ATP6V1A/STXBP3	2
BP	GO:0015833	peptide transport	1/33	53/4831	0.305981	0.648627	0.648627	STXBP3	1
BP	GO:0030595	leukocyte chemotaxis	1/33	53/4831	0.305981	0.648627	0.648627	SLC12A2	1
BP	GO:0070372	regulation of ERK1 and ERK2	1/33	53/4831	0.305981	0.648627	0.648627	RAP1B	1
BP	GO:0032940	secretion by cell	2/33	163/4831	0.306673	0.648627	0.648627	RAP1B/STXBP3	2
BP	GO:0001817	regulation of cytokine prod	2/33	164/4831	0.309226	0.651084	0.651084	PANX1/FOXP1	2
BP	GO:0007409	axonogenesis	1/33	55/4831	0.315535	0.651084	0.651084	FOXP1	1
BP	GO:0033500	carbohydrate homeostasis	1/33	55/4831	0.315535	0.651084	0.651084	STXBP3	1
BP	GO:0042593	glucose homeostasis	1/33	55/4831	0.315535	0.651084	0.651084	STXBP3	1
BP	GO:0001816	cytokine production	2/33	167/4831	0.316875	0.651084	0.651084	PANX1/FOXP1	2
BP	GO:0071705	nitrogen compound transport	4/33	418/4831	0.318548	0.651084	0.651084	VTI1A/HIKESHI/STXBP3/SLC12A2	4
BP	GO:0070371	ERK1 and ERK2 cascade	1/33	56/4831	0.320264	0.651084	0.651084	RAP1B	1
BP	GO:0006812	cation transport	2/33	169/4831	0.321966	0.651084	0.651084	PANX1/SLC12A2	2
BP	GO:1901698	response to nitrogen compou	2/33	170/4831	0.324509	0.651084	0.651084	RAP1B/STXBP3	2
BP	GO:0042113	B cell activation	1/33	57/4831	0.324962	0.651084	0.651084	FOXP1	1
BP	GO:0043434	response to peptide hormone	1/33	57/4831	0.324962	0.651084	0.651084	STXBP3	1
BP	GO:0034097	response to cytokine	2/33	172/4831	0.329589	0.651084	0.651084	STXBP3/SLC12A2	2
BP	GO:0042391	regulation of membrane pote	1/33	58/4831	0.329628	0.651084	0.651084	FOXP1	1
BP	GO:0042886	amide transport	1/33	58/4831	0.329628	0.651084	0.651084	STXBP3	1
BP	GO:0048638	regulation of developmental	1/33	58/4831	0.329628	0.651084	0.651084	FOXP1	1
BP	GO:1903311	regulation of mRNA metaboli	1/33	58/4831	0.329628	0.651084	0.651084	FAM172A	1
BP	GO:0034504	protein localization to nuc	1/33	59/4831	0.334263	0.652929	0.652929	HIKESHI	1

BP	GO:0140352	export from cell	2/33	174/4831	0.33466	0.652929	0.652929	RAP1B/STXBP3	2
BP	GO:1901137	carbohydrate derivative bio	2/33	174/4831	0.33466	0.652929	0.652929	ST6GAL1/B3GAT3	2
BP	GO:0019725	cellular homeostasis	2/33	176/4831	0.339722	0.659294	0.659294	ATP6V1A/STXBP3	2
BP	GO:0006399	tRNA metabolic process	1/33	61/4831	0.34344	0.659294	0.659294	LARS2	1
BP	GO:0030217	T cell differentiation	1/33	61/4831	0.34344	0.659294	0.659294	FOXP1	1
BP	GO:0061564	axon development	1/33	61/4831	0.34344	0.659294	0.659294	FOXP1	1
BP	GO:0002443	leukocyte mediated immunity	1/33	62/4831	0.347982	0.660061	0.660061	STXBP3	1
BP	GO:0034329	cell junction assembly	1/33	62/4831	0.347982	0.660061	0.660061	RAP1B	1
BP	GO:0046879	hormone secretion	1/33	62/4831	0.347982	0.660061	0.660061	STXBP3	1
BP	GO:0009914	hormone transport	1/33	63/4831	0.352494	0.665977	0.665977	STXBP3	1
BP	GO:0060326	cell chemotaxis	1/33	64/4831	0.356975	0.66962	0.66962	SLC12A2	1
BP	GO:0007517	muscle organ development	1/33	65/4831	0.361427	0.66962	0.66962	FOXP1	1
BP	GO:0009306	protein secretion	1/33	65/4831	0.361427	0.66962	0.66962	STXBP3	1
BP	GO:0048193	Golgi vesicle transport	1/33	65/4831	0.361427	0.66962	0.66962	RAB6A	1
BP	GO:0051235	maintenance of location	1/33	65/4831	0.361427	0.66962	0.66962	BSCL2	1
BP	GO:0035592	establishment of protein lo	1/33	66/4831	0.365848	0.670636	0.670636	STXBP3	1
BP	GO:0006913	nucleocytoplasmic transport	1/33	67/4831	0.37024	0.670636	0.670636	HIKESHI	1
BP	GO:0051169	nuclear transport	1/33	67/4831	0.37024	0.670636	0.670636	HIKESHI	1
BP	GO:0071692	protein localization to ext	1/33	67/4831	0.37024	0.670636	0.670636	STXBP3	1
BP	GO:0045321	leukocyte activation	2/33	189/4831	0.372354	0.670636	0.670636	FOXP1/STXBP3	2
BP	GO:0051049	regulation of transport	3/33	319/4831	0.373346	0.670636	0.670636	RAP1B/DNAJC13/STXBP3	3
BP	GO:0018205	peptidyl-lysine modificatio	1/33	68/4831	0.374602	0.670636	0.670636	CSKMT	1
BP	GO:0048667	cell morphogenesis involved	1/33	68/4831	0.374602	0.670636	0.670636	FOXP1	1
BP	GO:0051604	protein maturation	1/33	68/4831	0.374602	0.670636	0.670636	APH1B	1
BP	GO:0007265	Ras protein signal transduc	1/33	70/4831	0.383239	0.680495	0.680495	RAP1B	1
BP	GO:1901652	response to peptide	1/33	70/4831	0.383239	0.680495	0.680495	STXBP3	1
BP	GO:0046903	secretion	2/33	194/4831	0.384758	0.680495	0.680495	RAP1B/STXBP3	2
BP	GO:0009628	response to abiotic stimulu	2/33	195/4831	0.387227	0.680495	0.680495	HIKESHI/ATP6V1A	2
BP	GO:0070925	organelle assembly	2/33	195/4831	0.387227	0.680495	0.680495	FOXP1/BSCL2	2
BP	GO:0033365	protein localization to org	2/33	196/4831	0.389693	0.680951	0.680951	RAB6A/HIKESHI	2
BP	GO:0002697	regulation of immune effect	1/33	72/4831	0.39176	0.680951	0.680951	FOXP1	1
BP	GO:0048812	neuron projection morphogen	1/33	72/4831	0.39176	0.680951	0.680951	FOXP1	1
BP	GO:0042692	muscle cell differentiation	1/33	73/4831	0.395978	0.682724	0.682724	FOXP1	1
BP	GO:0120039	plasma membrane bounded cel	1/33	73/4831	0.395978	0.682724	0.682724	FOXP1	1
BP	GO:0006886	intracellular protein trans	2/33	199/4831	0.397066	0.682724	0.682724	VTI1A/HIKESHI	2

BP	GO:0048858	cell projection morphogenes	1/33	75/4831	0.404329	0.692721	0.692721	FOXP1	1
BP	GO:0007017	microtubule-based process	2/33	205/4831	0.411699	0.701804	0.701804	DYNLRB2/CEP350	2
BP	GO:0032990	cell part morphogenesis	1/33	77/4831	0.412567	0.701804	0.701804	FOXP1	1
BP	GO:0015031	protein transport	3/33	341/4831	0.415104	0.703616	0.703616	VTI1A/HIKESHI/STXBP3	3
BP	GO:0043254	regulation of protein-conta	1/33	78/4831	0.416645	0.703732	0.703732	RAP1B	1
BP	GO:0055085	transmembrane transport	2/33	208/4831	0.418957	0.704242	0.704242	STXBP3/SLC12A2	2
BP	GO:0060537	muscle tissue development	1/33	79/4831	0.420695	0.704242	0.704242	FOXP1	1
BP	GO:0035295	tube development	2/33	209/4831	0.421367	0.704242	0.704242	FOXP1/HIKESHI	2
BP	GO:0006310	DNA recombination	1/33	80/4831	0.424718	0.704914	0.704914	FOXP1	1
BP	GO:0050900	leukocyte migration	1/33	80/4831	0.424718	0.704914	0.704914	SLC12A2	1
BP	GO:0001775	cell activation	2/33	212/4831	0.428568	0.708843	0.708843	FOXP1/STXBP3	2
BP	GO:0030098	lymphocyte differentiation	1/33	82/4831	0.432683	0.710178	0.710178	FOXP1	1
BP	GO:0006520	cellular amino acid metabol	1/33	83/4831	0.436625	0.710178	0.710178	LARS2	1
BP	GO:0050678	regulation of epithelial ce	1/33	84/4831	0.44054	0.710178	0.710178	FOXP1	1
BP	GO:0001701	in utero embryonic developm	1/33	85/4831	0.44443	0.710178	0.710178	FOXP1	1
BP	GO:0043410	positive regulation of MAPK	1/33	85/4831	0.44443	0.710178	0.710178	RAP1B	1
BP	GO:0007268	chemical synaptic transmiss	1/33	86/4831	0.448293	0.710178	0.710178	RAP1B	1
BP	GO:0010876	lipid localization	1/33	86/4831	0.448293	0.710178	0.710178	BSCL2	1
BP	GO:0016570	histone modification	1/33	86/4831	0.448293	0.710178	0.710178	EED	1
BP	GO:0044089	positive regulation of cell	1/33	86/4831	0.448293	0.710178	0.710178	RAP1B	1
BP	GO:0098916	anterograde trans-synaptic	1/33	86/4831	0.448293	0.710178	0.710178	RAP1B	1
BP	GO:0043603	cellular amide metabolic pr	3/33	359/4831	0.448732	0.710178	0.710178	LARS2/APH1B/ST6GAL1	3
BP	GO:0000375	RNA splicing, via transeste	1/33	87/4831	0.45213	0.710178	0.710178	FAM172A	1
BP	GO:0000377	RNA splicing, via transeste	1/33	87/4831	0.45213	0.710178	0.710178	FAM172A	1
BP	GO:0000398	mRNA splicing, via spliceos	1/33	87/4831	0.45213	0.710178	0.710178	FAM172A	1
BP	GO:0099537	trans-synaptic signaling	1/33	87/4831	0.45213	0.710178	0.710178	RAP1B	1
BP	GO:0007399	nervous system development	3/33	362/4831	0.454275	0.710178	0.710178	FOXP1/STXBP3/SEMA4D	3
BP	GO:0045184	establishment of protein lo	3/33	363/4831	0.456118	0.710178	0.710178	VTI1A/HIKESHI/STXBP3	3
BP	GO:0070727	cellular macromolecule loca	3/33	363/4831	0.456118	0.710178	0.710178	VTI1A/RAB6A/HIKESHI	3
BP	GO:0099536	synaptic signaling	1/33	89/4831	0.459726	0.713471	0.713471	RAP1B	1
BP	GO:0019752	carboxylic acid metabolic p	2/33	226/4831	0.461586	0.71404	0.71404	LARS2/ST6GAL1	2
BP	GO:0006820	anion transport	1/33	90/4831	0.463486	0.714665	0.714665	SLC12A2	1
BP	GO:0034645	cellular macromolecule bios	3/33	368/4831	0.4653	0.715156	0.715156	LARS2/ST6GAL1/B3GAT3	3
BP	GO:0072594	establishment of protein lo	1/33	91/4831	0.46722	0.715516	0.715516	HIKESHI	1
BP	GO:0043436	oxoacid metabolic process	2/33	229/4831	0.468528	0.715516	0.715516	LARS2/ST6GAL1	2

BP	GO:0007018	microtubule-based movement	1/33	93/4831	0.474614	0.720208	0.720208	DYNLRB2	1
BP	GO:0007507	heart development	1/33	93/4831	0.474614	0.720208	0.720208	FOXP1	1
BP	GO:0000904	cell morphogenesis involved	1/33	95/4831	0.481908	0.72146	0.72146	FOXP1	1
BP	GO:0016042	lipid catabolic process	1/33	95/4831	0.481908	0.72146	0.72146	CLPS	1
BP	GO:0050673	epithelial cell proliferati	1/33	95/4831	0.481908	0.72146	0.72146	FOXP1	1
BP	GO:1903131	mononuclear cell differenti	1/33	95/4831	0.481908	0.72146	0.72146	FOXP1	1
BP	GO:0060429	epithelium development	2/33	236/4831	0.484531	0.72146	0.72146	FOXP1/RAP1B	2
BP	GO:0032989	cellular component morphoge	1/33	96/4831	0.485518	0.72146	0.72146	FOXP1	1
BP	GO:0006082	organic acid metabolic proc	2/33	237/4831	0.486795	0.72146	0.72146	LARS2/ST6GAL1	2
BP	GO:0098662	inorganic cation transmembr	1/33	97/4831	0.489103	0.72146	0.72146	SLC12A2	1
BP	GO:0006914	autophagy	1/33	98/4831	0.492665	0.72146	0.72146	VTI1A	1
BP	GO:0061919	process utilizing autophagi	1/33	98/4831	0.492665	0.72146	0.72146	VTI1A	1
BP	GO:0009890	negative regulation of bios	2/33	240/4831	0.493551	0.72146	0.72146	GCHFR/DNAJC17	2
BP	GO:0010628	positive regulation of gene	2/33	240/4831	0.493551	0.72146	0.72146	PANX1/FOXP1	2
BP	GO:0071407	cellular response to organi	1/33	100/4831	0.499715	0.72603	0.72603	RAP1B	1
BP	GO:0071417	cellular response to organo	1/33	100/4831	0.499715	0.72603	0.72603	RAP1B	1
BP	GO:0034330	cell junction organization	1/33	102/4831	0.506671	0.732026	0.732026	RAP1B	1
BP	GO:0007155	cell adhesion	2/33	246/4831	0.506905	0.732026	0.732026	LGALS7/STXBP3	2
BP	GO:0048878	chemical homeostasis	2/33	249/4831	0.513501	0.737143	0.737143	ATP6V1A/STXBP3	2
BP	GO:0010817	regulation of hormone level	1/33	104/4831	0.513533	0.737143	0.737143	STXBP3	1
BP	GO:0034762	regulation of transmembrane	1/33	105/4831	0.516929	0.737995	0.737995	STXBP3	1
BP	GO:0007267	cell-cell signaling	2/33	252/4831	0.520043	0.737995	0.737995	RAP1B/STXBP3	2
BP	GO:0000122	negative regulation of tran	1/33	106/4831	0.520302	0.737995	0.737995	DNAJC17	1
BP	GO:0098660	inorganic ion transmembrane	1/33	106/4831	0.520302	0.737995	0.737995	SLC12A2	1
BP	GO:0007420	brain development	1/33	108/4831	0.52698	0.743057	0.743057	STXBP3	1
BP	GO:0098655	cation transmembrane transp	1/33	108/4831	0.52698	0.743057	0.743057	SLC12A2	1
BP	GO:0008380	RNA splicing	1/33	109/4831	0.530285	0.745518	0.745518	FAM172A	1
BP	GO:0007264	small GTPase mediated signa	1/33	111/4831	0.536828	0.750303	0.750303	RAP1B	1
BP	GO:0042110	T cell activation	1/33	111/4831	0.536828	0.750303	0.750303	FOXP1	1
BP	GO:0007610	behavior	1/33	112/4831	0.540066	0.752629	0.752629	FOXP1	1
BP	GO:1901699	cellular response to nitrog	1/33	113/4831	0.543283	0.75491	0.75491	RAP1B	1
BP	GO:1901700	response to oxygen-containi	2/33	268/4831	0.553992	0.767508	0.767508	RAP1B/STXBP3	2
BP	GO:0060322	head development	1/33	117/4831	0.555931	0.767508	0.767508	STXBP3	1
BP	GO:0006875	cellular metal ion homeosta	1/33	118/4831	0.55904	0.767508	0.767508	ATP6V1A	1
BP	GO:0061061	muscle structure developmen	1/33	118/4831	0.55904	0.767508	0.767508	FOXP1	1

BP	GO:0006811	ion transport	2/33	273/4831	0.56427	0.767508	0.767508	PANX1/SLC12A2	2
BP	GO:0034470	ncRNA processing	1/33	120/4831	0.565194	0.767508	0.767508	INTS5	1
BP	GO:0040008	regulation of growth	1/33	120/4831	0.565194	0.767508	0.767508	FOXP1	1
BP	GO:0043408	regulation of MAPK cascade	1/33	120/4831	0.565194	0.767508	0.767508	RAP1B	1
BP	GO:0006396	RNA processing	2/33	276/4831	0.570359	0.770692	0.770692	FAM172A/INTS5	2
BP	GO:0002521	leukocyte differentiation	1/33	122/4831	0.571265	0.770692	0.770692	FOXP1	1
BP	GO:0051240	positive regulation of mult	2/33	277/4831	0.572375	0.770692	0.770692	PANX1/FOXP1	2
BP	GO:0051301	cell division	1/33	123/4831	0.574269	0.770746	0.770746	DCTN3	1
BP	GO:0009719	response to endogenous stim	2/33	279/4831	0.57639	0.770746	0.770746	RAP1B/STXBP3	2
BP	GO:0048589	developmental growth	1/33	124/4831	0.577253	0.770746	0.770746	FOXP1	1
BP	GO:0045087	innate immune response	1/33	126/4831	0.583161	0.773891	0.773891	STXBP3	1
BP	GO:0006397	mRNA processing	1/33	127/4831	0.586084	0.773891	0.773891	FAM172A	1
BP	GO:0014070	response to organic cyclic	1/33	127/4831	0.586084	0.773891	0.773891	RAP1B	1
BP	GO:0031175	neuron projection developme	1/33	127/4831	0.586084	0.773891	0.773891	FOXP1	1
BP	GO:0045596	negative regulation of cell	1/33	128/4831	0.588988	0.775096	0.775096	FOXP1	1
BP	GO:1901135	carbohydrate derivative met	2/33	288/4831	0.594132	0.775096	0.775096	ST6GAL1/B3GAT3	2
BP	GO:0000226	microtubule cytoskeleton or	1/33	130/4831	0.594737	0.775096	0.775096	CEP350	1
BP	GO:0030003	cellular cation homeostasis	1/33	130/4831	0.594737	0.775096	0.775096	ATP6V1A	1
BP	GO:0009725	response to hormone	1/33	131/4831	0.597581	0.775096	0.775096	STXBP3	1
BP	GO:0055065	metal ion homeostasis	1/33	131/4831	0.597581	0.775096	0.775096	ATP6V1A	1
BP	GO:0006357	regulation of transcription	2/33	291/4831	0.599928	0.775096	0.775096	DNAJC17/FOXP1	2
BP	GO:0006873	cellular ion homeostasis	1/33	132/4831	0.600407	0.775096	0.775096	ATP6V1A	1
BP	GO:0006518	peptide metabolic process	2/33	292/4831	0.601847	0.775096	0.775096	LARS2/APH1B	2
BP	GO:0000165	MAPK cascade	1/33	133/4831	0.603213	0.775096	0.775096	RAP1B	1
BP	GO:0051707	response to other organism	2/33	296/4831	0.609457	0.781021	0.781021	CLPS/STXBP3	2
BP	GO:0043207	response to external biotic	2/33	297/4831	0.611344	0.781343	0.781343	CLPS/STXBP3	2
BP	GO:0009617	response to bacterium	1/33	139/4831	0.619654	0.789359	0.789359	CLPS	1
BP	GO:0071310	cellular response to organi	3/33	460/4831	0.620918	0.789359	0.789359	RAP1B/STXBP3/SLC12A2	3
BP	GO:0009607	response to biotic stimulus	2/33	307/4831	0.629845	0.798583	0.798583	CLPS/STXBP3	2
BP	GO:0000902	cell morphogenesis	1/33	145/4831	0.635433	0.801416	0.801416	FOXP1	1
BP	GO:0030001	metal ion transport	1/33	145/4831	0.635433	0.801416	0.801416	SLC12A2	1
BP	GO:0043009	chordate embryonic developm	1/33	147/4831	0.64055	0.802564	0.802564	FOXP1	1
BP	GO:0060341	regulation of cellular loca	1/33	147/4831	0.64055	0.802564	0.802564	DNAJC13	1
BP	GO:0005975	carbohydrate metabolic proc	1/33	149/4831	0.645597	0.802564	0.802564	GANAB	1
BP	GO:0055080	cation homeostasis	1/33	149/4831	0.645597	0.802564	0.802564	ATP6V1A	1

BP	GO:0044419	biological process involved	2/33	316/4831	0.645934	0.802564	0.802564	CLPS/STXBP3	2
BP	GO:0006366	transcription by RNA polyme	2/33	317/4831	0.647689	0.802564	0.802564	DNAJC17/FOXP1	2
BP	GO:0009792	embryo development ending i	1/33	150/4831	0.648095	0.802564	0.802564	FOXP1	1
BP	GO:0040011	locomotion	2/33	319/4831	0.651179	0.804299	0.804299	FOXP1/SLC12A2	2
BP	GO:0048666	neuron development	1/33	152/4831	0.65304	0.804519	0.804519	FOXP1	1
BP	GO:0098771	inorganic ion homeostasis	1/33	153/4831	0.655487	0.805457	0.805457	ATP6V1A	1
BP	GO:0050801	ion homeostasis	1/33	155/4831	0.660331	0.809329	0.809329	ATP6V1A	1
BP	GO:0034220	ion transmembrane transport	1/33	157/4831	0.665109	0.811026	0.811026	SLC12A2	1
BP	GO:0046649	lymphocyte activation	1/33	157/4831	0.665109	0.811026	0.811026	FOXP1	1
BP	GO:1902531	regulation of intracellular	2/33	331/4831	0.671575	0.816826	0.816826	MOB3B/RAP1B	2
BP	GO:0071702	organic substance transport	3/33	498/4831	0.676198	0.820362	0.820362	VTI1A/HIKESHI/STXBP3	3
BP	GO:0045944	positive regulation of tran	1/33	164/4831	0.681324	0.822526	0.822526	FOXP1	1
BP	GO:0006629	lipid metabolic process	2/33	337/4831	0.681423	0.822526	0.822526	CLPS/BSCL2	2
BP	GO:0043085	positive regulation of cata	1/33	171/4831	0.696776	0.83894	0.83894	APH1B	1
BP	GO:0048468	cell development	2/33	348/4831	0.698881	0.839359	0.839359	FOXP1/RAP1B	2
BP	GO:0050877	nervous system process	1/33	175/4831	0.705277	0.843914	0.843914	VTI1A	1
BP	GO:0045892	negative regulation of DNA-	1/33	177/4831	0.709441	0.843914	0.843914	DNAJC17	1
BP	GO:0051130	positive regulation of cell	1/33	178/4831	0.711501	0.843914	0.843914	RAP1B	1
BP	GO:1902679	negative regulation of RNA	1/33	178/4831	0.711501	0.843914	0.843914	DNAJC17	1
BP	GO:1903507	negative regulation of nucl	1/33	178/4831	0.711501	0.843914	0.843914	DNAJC17	1
BP	GO:0006955	immune response	2/33	361/4831	0.71853	0.849307	0.849307	FOXP1/STXBP3	2
BP	GO:0051093	negative regulation of deve	1/33	182/4831	0.719601	0.849307	0.849307	FOXP1	1
BP	GO:0040007	growth	1/33	183/4831	0.721592	0.849559	0.849559	FOXP1	1
BP	GO:0002684	positive regulation of immu	1/33	185/4831	0.725531	0.852098	0.852098	FOXP1	1
BP	GO:1902533	positive regulation of intr	1/33	187/4831	0.729417	0.854562	0.854562	RAP1B	1
BP	GO:0044087	regulation of cellular comp	1/33	189/4831	0.733249	0.856951	0.856951	RAP1B	1
BP	GO:0009888	tissue development	2/33	375/4831	0.738529	0.857266	0.857266	FOXP1/RAP1B	2
BP	GO:0042592	homeostatic process	2/33	375/4831	0.738529	0.857266	0.857266	ATP6V1A/STXBP3	2
BP	GO:0008284	positive regulation of cell	1/33	192/4831	0.738899	0.857266	0.857266	FOXP1	1
BP	GO:0016071	mRNA metabolic process	1/33	194/4831	0.742601	0.859475	0.859475	FAM172A	1
BP	GO:0030182	neuron differentiation	1/33	197/4831	0.748059	0.861586	0.861586	FOXP1	1
BP	GO:0072359	circulatory system developm	1/33	198/4831	0.749853	0.861586	0.861586	FOXP1	1
BP	GO:0030097	hemopoiesis	1/33	199/4831	0.751634	0.861586	0.861586	FOXP1	1
BP	GO:0051253	negative regulation of RNA	1/33	199/4831	0.751634	0.861586	0.861586	DNAJC17	1
BP	GO:0051241	negative regulation of mult	1/33	202/4831	0.756906	0.865553	0.865553	FOXP1	1

BP	GO:0008283	cell population proliferati	2/33	393/4831	0.762526	0.869239	0.869239	FOXP1/RAP1B	2
BP	GO:1901701	cellular response to oxygen	1/33	206/4831	0.763767	0.869239	0.869239	RAP1B	1
BP	GO:0048534	hematopoietic or lymphoid o	1/33	208/4831	0.767126	0.870989	0.870989	FOXP1	1
BP	GO:0048699	generation of neurons	1/33	211/4831	0.772079	0.874535	0.874535	FOXP1	1
BP	GO:0002520	immune system development	1/33	218/4831	0.78324	0.88508	0.88508	FOXP1	1
BP	GO:0098542	defense response to other o	1/33	220/4831	0.786331	0.886477	0.886477	STXBP3	1
BP	GO:0010558	negative regulation of macr	1/33	223/4831	0.790887	0.889515	0.889515	DNAJC17	1
BP	GO:0045934	negative regulation of nucl	1/33	225/4831	0.793871	0.890776	0.890776	DNAJC17	1
BP	GO:0009790	embryo development	1/33	231/4831	0.80258	0.898438	0.898438	FOXP1	1
BP	GO:0048646	anatomical structure format	1/33	234/4831	0.806799	0.901051	0.901051	FOXP1	1
BP	GO:0031327	negative regulation of cell	1/33	236/4831	0.809563	0.902031	0.902031	DNAJC17	1
BP	GO:0045893	positive regulation of DNA-	1/33	241/4831	0.816307	0.902445	0.902445	FOXP1	1
BP	GO:1903508	positive regulation of nucl	1/33	241/4831	0.816307	0.902445	0.902445	FOXP1	1
BP	GO:0120036	plasma membrane bounded cel	1/33	242/4831	0.817628	0.902445	0.902445	FOXP1	1
BP	GO:0006412	translation	1/33	243/4831	0.818939	0.902445	0.902445	LARS2	1
BP	GO:1902680	positive regulation of RNA	1/33	244/4831	0.820241	0.902445	0.902445	FOXP1	1
BP	GO:0016477	cell migration	1/33	247/4831	0.824094	0.902445	0.902445	SLC12A2	1
BP	GO:0043043	peptide biosynthetic proces	1/33	249/4831	0.826618	0.902445	0.902445	LARS2	1
BP	GO:0022402	cell cycle process	1/33	251/4831	0.829107	0.902445	0.902445	DCTN3	1
BP	GO:0022008	neurogenesis	1/33	254/4831	0.832775	0.902445	0.902445	FOXP1	1
BP	GO:0030030	cell projection organizatio	1/33	254/4831	0.832775	0.902445	0.902445	FOXP1	1
BP	GO:0071495	cellular response to endoge	1/33	254/4831	0.832775	0.902445	0.902445	RAP1B	1
BP	GO:0044093	positive regulation of mole	1/33	255/4831	0.833981	0.902445	0.902445	APH1B	1
BP	GO:0044281	small molecule metabolic pr	2/33	460/4831	0.836321	0.902445	0.902445	LARS2/ST6GAL1	2
BP	GO:0006259	DNA metabolic process	1/33	257/4831	0.836367	0.902445	0.902445	FOXP1	1
BP	GO:2000026	regulation of multicellular	1/33	259/4831	0.83872	0.902946	0.902946	FOXP1	1
BP	GO:0010648	negative regulation of cell	1/33	265/4831	0.845583	0.907449	0.907449	RAP1B	1
BP	GO:0023057	negative regulation of sign	1/33	266/4831	0.846699	0.907449	0.907449	RAP1B	1
BP	GO:0051254	positive regulation of RNA	1/33	274/4831	0.85535	0.91467	0.91467	FOXP1	1
BP	GO:0045595	regulation of cell differen	1/33	280/4831	0.861526	0.915223	0.915223	FOXP1	1
BP	GO:0043604	amide biosynthetic process	1/33	282/4831	0.863527	0.915223	0.915223	LARS2	1
BP	GO:0048870	cell motility	1/33	282/4831	0.863527	0.915223	0.915223	SLC12A2	1
BP	GO:0051674	localization of cell	1/33	282/4831	0.863527	0.915223	0.915223	SLC12A2	1
BP	GO:0010557	positive regulation of macr	1/33	285/4831	0.866476	0.915296	0.915296	FOXP1	1
BP	GO:0009967	positive regulation of sign	1/33	287/4831	0.868408	0.915296	0.915296	RAP1B	1

BP	GO:0050790	regulation of catalytic act	1/33	288/4831	0.869363	0.915296	0.915296	APH1B	1
BP	GO:0006508	proteolysis	1/33	290/4831	0.871255	0.915296	0.915296	APH1B	1
BP	GO:0003008	system process	1/33	302/4831	0.882056	0.923246	0.923246	VTI1A	1
BP	GO:0031328	positive regulation of cell	1/33	303/4831	0.882916	0.923246	0.923246	FOXP1	1
BP	GO:0002682	regulation of immune system	1/33	305/4831	0.884616	0.923246	0.923246	FOXP1	1
BP	GO:0009891	positive regulation of bios	1/33	312/4831	0.890382	0.924046	0.924046	FOXP1	1
BP	GO:0010647	positive regulation of cell	1/33	312/4831	0.890382	0.924046	0.924046	RAP1B	1
BP	GO:0023056	positive regulation of sign	1/33	313/4831	0.891182	0.924046	0.924046	RAP1B	1
BP	GO:0045935	positive regulation of nucl	1/33	317/4831	0.894328	0.9253	0.9253	FOXP1	1
BP	GO:0042127	regulation of cell populati	1/33	331/4831	0.904658	0.933967	0.933967	FOXP1	1
BP	GO:0006952	defense response	1/33	352/4831	0.918341	0.944633	0.944633	STXBP3	1
BP	GO:0065003	protein-containing complex	1/33	353/4831	0.918942	0.944633	0.944633	RAP1B	1
BP	GO:0051172	negative regulation of nitr	1/33	358/4831	0.921887	0.945626	0.945626	DNAJC17	1
BP	GO:0007049	cell cycle	1/33	367/4831	0.926927	0.948761	0.948761	DCTN3	1
BP	GO:0031324	negative regulation of cell	1/33	384/4831	0.935602	0.955593	0.955593	DNAJC17	1
BP	GO:0043933	protein-containing complex	1/33	398/4831	0.941988	0.958897	0.958897	RAP1B	1
BP	GO:0033554	cellular response to stress	1/33	400/4831	0.942848	0.958897	0.958897	HIKESHI	1
BP	GO:0051128	regulation of cellular comp	1/33	417/4831	0.94968	0.963794	0.963794	RAP1B	1
BP	GO:0048584	positive regulation of resp	1/33	428/4831	0.953671	0.965482	0.965482	RAP1B	1
BP	GO:0065009	regulation of molecular fun	1/33	433/4831	0.955383	0.965482	0.965482	APH1B	1
BP	GO:0050793	regulation of developmental	1/33	451/4831	0.96105	0.968979	0.968979	FOXP1	1
BP	GO:0010605	negative regulation of macr	1/33	462/4831	0.964162	0.968979	0.968979	DNAJC17	1
BP	GO:0044248	cellular catabolic process	1/33	468/4831	0.965757	0.968979	0.968979	VTI1A	1
BP	GO:1901575	organic substance catabolic	1/33	478/4831	0.968263	0.968979	0.968979	CLPS	1
BP	GO:0009653	anatomical structure morpho	1/33	481/4831	0.968979	0.968979	0.968979	FOXP1	1
CC	GO:0033178	proton-transporting two-sec	2/32	13/5930	0.002121	0.078071	0.069161	ATP6V1C1/ATP6V1A	2
CC	GO:0031410	cytoplasmic vesicle	7/32	386/5930	0.003809	0.078071	0.069161	GCHFR/RAB6A/APH1B/DNAJC13/ATP6V1A	7
CC	GO:0097708	intracellular vesicle	7/32	386/5930	0.003809	0.078071	0.069161	GCHFR/RAB6A/APH1B/DNAJC13/ATP6V1A	7
CC	GO:0033176	proton-transporting V-type	2/32	19/5930	0.004556	0.078071	0.069161	ATP6V1C1/ATP6V1A	2
CC	GO:0016324	apical plasma membrane	3/32	64/5930	0.004758	0.078071	0.069161	ATP6V1A/STXBP3/SLC12A2	3
CC	GO:0031982	vesicle	7/32	403/5930	0.004838	0.078071	0.069161	GCHFR/RAB6A/APH1B/DNAJC13/ATP6V1A	7
CC	GO:0030133	transport vesicle	3/32	67/5930	0.005411	0.078071	0.069161	RAB6A/APH1B/ATP6V1A	3
CC	GO:0045177	apical part of cell	3/32	81/5930	0.009157	0.115613	0.102419	ATP6V1A/STXBP3/SLC12A2	3
CC	GO:0099503	secretory vesicle	3/32	85/5930	0.010448	0.117248	0.103867	RAB6A/ATP6V1A/STXBP3	3
CC	GO:0005875	microtubule associated comp	2/32	33/5930	0.013422	0.129161	0.114421	DCTN3/DYNLRB2	2

CC	GO:0016469	proton-transporting two-sec	2/32	34/5930	0.014213	0.129161	0.114421	ATP6V1C1/ATP6V1A	2
CC	GO:0000139	Golgi membrane	3/32	98/5930	0.015346	0.129161	0.114421	RAB6A/ST6GAL1/B3GAT3	3
CC	GO:0030141	secretory granule	2/32	57/5930	0.03745	0.261379	0.23155	ATP6V1A/STXBP3	2
CC	GO:0120025	plasma membrane bounded cel	5/32	350/5930	0.037616	0.261379	0.23155	CDC42BPA/PANX1/GCHFR/ATP6V1A/SLC1	5
CC	GO:0098588	bounding membrane of organe	5/32	353/5930	0.038819	0.261379	0.23155	RAB6A/ST6GAL1/DNAJC13/ATP6V1A/B3G	5
CC	GO:0042995	cell projection	5/32	365/5930	0.043868	0.276916	0.245314	CDC42BPA/PANX1/GCHFR/ATP6V1A/SLC1	5
CC	GO:0022624	proteasome accessory comple	1/32	12/5930	0.062924	0.335282	0.297019	PSMD12	1
CC	GO:0031519	PcG protein complex	1/32	12/5930	0.062924	0.335282	0.297019	EED	1
CC	GO:0005921	gap junction	1/32	13/5930	0.067991	0.335282	0.297019	PANX1	1
CC	GO:0016471	vacuolar proton-transportin	1/32	13/5930	0.067991	0.335282	0.297019	ATP6V1A	1
CC	GO:0036477	somatodendritic compartment	2/32	82/5930	0.071741	0.335282	0.297019	GCHFR/SLC12A2	2
CC	GO:0035097	histone methyltransferase c	1/32	14/5930	0.073032	0.335282	0.297019	EED	1
CC	GO:0005794	Golgi apparatus	4/32	312/5930	0.08489	0.345844	0.306376	VTI1A/RAB6A/ST6GAL1/B3GAT3	4
CC	GO:0005911	cell-cell junction	2/32	98/5930	0.097418	0.345844	0.306376	PANX1/RAP1B	2
CC	GO:0000315	organellar large ribosomal	1/32	20/5930	0.102724	0.345844	0.306376	MRPL48	1
CC	GO:0005762	mitochondrial large ribosom	1/32	20/5930	0.102724	0.345844	0.306376	MRPL48	1
CC	GO:0030286	dynein complex	1/32	20/5930	0.102724	0.345844	0.306376	DYNLRB2	1
CC	GO:0005811	lipid droplet	1/32	21/5930	0.107583	0.345844	0.306376	RAP1B	1
CC	GO:0005902	microvillus	1/32	22/5930	0.112416	0.345844	0.306376	ATP6V1A	1
CC	GO:0016328	lateral plasma membrane	1/32	23/5930	0.117223	0.345844	0.306376	SLC12A2	1
CC	GO:0031901	early endosome membrane	1/32	23/5930	0.117223	0.345844	0.306376	DNAJC13	1
CC	GO:0034708	methyltransferase complex	1/32	24/5930	0.122005	0.345844	0.306376	EED	1
CC	GO:0042470	melanosome	1/32	24/5930	0.122005	0.345844	0.306376	GCHFR	1
CC	GO:0045335	phagocytic vesicle	1/32	24/5930	0.122005	0.345844	0.306376	STXBP3	1
CC	GO:0048770	pigment granule	1/32	24/5930	0.122005	0.345844	0.306376	GCHFR	1
CC	GO:0098590	plasma membrane region	3/32	228/5930	0.123271	0.345844	0.306376	ATP6V1A/STXBP3/SLC12A2	3
CC	GO:0005776	autophagosome	1/32	29/5930	0.145543	0.386124	0.342058	VTI1A	1
CC	GO:0070382	exocytic vesicle	1/32	29/5930	0.145543	0.386124	0.342058	RAB6A	1
CC	GO:0000313	organellar ribosome	1/32	31/5930	0.154786	0.386124	0.342058	MRPL48	1
CC	GO:0005761	mitochondrial ribosome	1/32	31/5930	0.154786	0.386124	0.342058	MRPL48	1
CC	GO:0070161	anchoring junction	2/32	131/5930	0.156743	0.386124	0.342058	PANX1/RAP1B	2
CC	GO:0030139	endocytic vesicle	1/32	34/5930	0.168468	0.405126	0.358892	STXBP3	1
CC	GO:0043005	neuron projection	2/32	142/5930	0.17785	0.417627	0.369966	GCHFR/SLC12A2	2
CC	GO:0000502	proteasome complex	1/32	37/5930	0.181936	0.417627	0.369966	PSMD12	1
CC	GO:0005773	vacuole	2/32	152/5930	0.197422	0.425885	0.377283	VTI1A/ATP6V1A	2

CC	GO:0030027	lamellipodium	1/32	41/5930	0.199566	0.425885	0.377283	CDC42BPA	1
CC	GO:0005802	trans-Golgi network	1/32	43/5930	0.208242	0.425885	0.377283	RAB6A	1
CC	GO:1905369	endopeptidase complex	1/32	43/5930	0.208242	0.425885	0.377283	PSMD12	1
CC	GO:0030659	cytoplasmic vesicle membran	2/32	161/5930	0.215276	0.425885	0.377283	DNAJC13/SLC12A2	2
CC	GO:0009925	basal plasma membrane	1/32	45/5930	0.216826	0.425885	0.377283	SLC12A2	1
CC	GO:0098858	actin-based cell projection	1/32	45/5930	0.216826	0.425885	0.377283	ATP6V1A	1
CC	GO:0012506	vesicle membrane	2/32	163/5930	0.219268	0.425885	0.377283	DNAJC13/SLC12A2	2
CC	GO:0043025	neuronal cell body	1/32	49/5930	0.233726	0.428674	0.379753	SLC12A2	1
CC	GO:0045178	basal part of cell	1/32	49/5930	0.233726	0.428674	0.379753	SLC12A2	1
CC	GO:0015630	microtubule cytoskeleton	3/32	314/5930	0.238747	0.428674	0.379753	DCTN3/DYNLRB2/CEP350	3
CC	GO:0030425	dendrite	1/32	52/5930	0.246169	0.428674	0.379753	GCHFR	1
CC	GO:0097447	dendritic tree	1/32	52/5930	0.246169	0.428674	0.379753	GCHFR	1
CC	GO:1905368	peptidase complex	1/32	52/5930	0.246169	0.428674	0.379753	PSMD12	1
CC	GO:0015934	large ribosomal subunit	1/32	54/5930	0.254355	0.435422	0.38573	MRPL48	1
CC	GO:0031965	nuclear membrane	1/32	56/5930	0.262455	0.4418	0.391381	GCHFR	1
CC	GO:0030054	cell junction	3/32	334/5930	0.268181	0.444037	0.393363	PANX1/RAP1B/STXBP3	3
CC	GO:0005769	early endosome	1/32	60/5930	0.2784	0.446324	0.395389	DNAJC13	1
CC	GO:0098793	presynapse	1/32	60/5930	0.2784	0.446324	0.395389	STXBP3	1
CC	GO:0044297	cell body	1/32	65/5930	0.297862	0.462832	0.410013	SLC12A2	1
CC	GO:0098791	Golgi apparatus subcompartm	1/32	65/5930	0.297862	0.462832	0.410013	RAB6A	1
CC	GO:0098796	membrane protein complex	3/32	359/5930	0.305627	0.467702	0.414327	APH1B/ATP6V1C1/ATP6V1A	3
CC	GO:0005774	vacuolar membrane	1/32	75/5930	0.335273	0.503374	0.445928	ATP6V1A	1
CC	GO:0031252	cell leading edge	1/32	76/5930	0.338906	0.503374	0.445928	CDC42BPA	1
CC	GO:0044391	ribosomal subunit	1/32	78/5930	0.346114	0.506631	0.448813	MRPL48	1
CC	GO:0010008	endosome membrane	1/32	85/5930	0.370749	0.529717	0.469265	DNAJC13	1
CC	GO:0005759	mitochondrial matrix	1/32	86/5930	0.374194	0.529717	0.469265	MRPL48	1
CC	GO:0015629	actin cytoskeleton	1/32	87/5930	0.37762	0.529717	0.469265	DCTN3	1
CC	GO:0016607	nuclear speck	1/32	90/5930	0.387792	0.531577	0.470912	HIKESHI	1
CC	GO:0005874	microtubule	1/32	91/5930	0.391146	0.531577	0.470912	DYNLRB2	1
CC	GO:1902494	catalytic complex	3/32	418/5930	0.394735	0.531577	0.470912	PSMD12/EED/DYNLRB2	3
CC	GO:0005635	nuclear envelope	1/32	94/5930	0.401104	0.533046	0.472213	GCHFR	1
CC	GO:0098798	mitochondrial protein-conta	1/32	110/5930	0.451601	0.59236	0.524759	MRPL48	1
CC	GO:0031984	organelle subcompartment	2/32	294/5930	0.476024	0.61639	0.546046	RAB6A/BSCL2	2
CC	GO:0005615	extracellular space	2/32	316/5930	0.514926	0.658323	0.583193	LGALS7/FBLN5	2
CC	GO:0099513	polymeric cytoskeletal fibe	1/32	139/5930	0.532815	0.672679	0.595912	DYNLRB2	1

CC	GO:0005813	centrosome	1/32	147/5930	0.553085	0.681238	0.603494	CEP350	1
CC	GO:0098797	plasma membrane protein com	1/32	147/5930	0.553085	0.681238	0.603494	APH1B	1
CC	GO:0005840	ribosome	1/32	155/5930	0.572501	0.696658	0.617154	MRPL48	1
CC	GO:0140513	nuclear protein-containing	2/32	370/5930	0.602493	0.724426	0.641753	EED/INTS5	2
CC	GO:0016604	nuclear body	1/32	172/5930	0.611086	0.726114	0.643248	HIKESHI	1
CC	GO:0005815	microtubule organizing cent	1/32	189/5930	0.646287	0.740738	0.656204	CEP350	1
CC	GO:0005768	endosome	1/32	192/5930	0.65217	0.740738	0.656204	DNAJC13	1
CC	GO:0005783	endoplasmic reticulum	2/32	406/5930	0.654302	0.740738	0.656204	PANX1/BSCL2	2
CC	GO:0099512	supramolecular fiber	1/32	194/5930	0.656039	0.740738	0.656204	DYNLRB2	1
CC	GO:0099081	supramolecular polymer	1/32	197/5930	0.661764	0.740738	0.656204	DYNLRB2	1
CC	GO:0045202	synapse	1/32	200/5930	0.667398	0.740738	0.656204	STXBP3	1
CC	GO:1990234	transferase complex	1/32	207/5930	0.680191	0.746731	0.661513	EED	1
CC	GO:1990904	ribonucleoprotein complex	1/32	223/5930	0.707667	0.762988	0.675914	MRPL48	1
CC	GO:0140535	intracellular protein-conta	1/32	230/5930	0.718956	0.762988	0.675914	PSMD12	1
CC	GO:0005789	endoplasmic reticulum membr	1/32	232/5930	0.722103	0.762988	0.675914	BSCL2	1
CC	GO:0098827	endoplasmic reticulum subco	1/32	234/5930	0.725216	0.762988	0.675914	BSCL2	1
CC	GO:0042175	nuclear outer membrane-endo	1/32	243/5930	0.738811	0.769277	0.681486	BSCL2	1
CC	GO:0099080	supramolecular complex	1/32	276/5930	0.783302	0.80728	0.715152	DYNLRB2	1
CC	GO:0031967	organelle envelope	1/32	316/5930	0.82745	0.835725	0.74035	GCHFR	1
CC	GO:0031975	envelope	1/32	316/5930	0.82745	0.835725	0.74035	GCHFR	1
CC	GO:0005739	mitochondrion	1/32	475/5930	0.931371	0.931371	0.825081	MRPL48	1
MF	GO:0031072	heat shock protein binding	2/32	27/5513	0.010466	0.345131	0.328845	HIKESHI/SLC12A2	2
MF	GO:0005261	cation channel activity	3/32	90/5513	0.014827	0.345131	0.328845	PANX1/ATP6V1A/SLC12A2	3
MF	GO:0022890	inorganic cation transmembr	4/32	176/5513	0.017964	0.345131	0.328845	PANX1/ATP6V1C1/ATP6V1A/SLC12A2	4
MF	GO:0005215	transporter activity	6/32	388/5513	0.022163	0.345131	0.328845	PANX1/SLC35F1/PITPNC1/ATP6V1C1/AT	6
MF	GO:0008324	cation transmembrane transp	4/32	191/5513	0.023514	0.345131	0.328845	PANX1/ATP6V1C1/ATP6V1A/SLC12A2	4
MF	GO:0030674	protein-macromolecule adapt	2/32	43/5513	0.025421	0.345131	0.328845	VTI1A/APH1B	2
MF	GO:0015078	proton transmembrane transp	2/32	46/5513	0.028825	0.345131	0.328845	ATP6V1C1/ATP6V1A	2
MF	GO:0060090	molecular adaptor activity	2/32	55/5513	0.040047	0.345131	0.328845	VTI1A/APH1B	2
MF	GO:0005216	ion channel activity	3/32	136/5513	0.043265	0.345131	0.328845	PANX1/ATP6V1A/SLC12A2	3
MF	GO:0015318	inorganic molecular entity	4/32	235/5513	0.045387	0.345131	0.328845	PANX1/ATP6V1C1/ATP6V1A/SLC12A2	4
MF	GO:0016874	ligase activity	2/32	62/5513	0.049739	0.345131	0.328845	LARS2/ATP6V1A	2
MF	GO:0022853	active ion transmembrane tr	2/32	64/5513	0.05265	0.345131	0.328845	ATP6V1A/SLC12A2	2
MF	GO:0008373	sialyltransferase activity	1/32	10/5513	0.056597	0.345131	0.328845	ST6GAL1	1
MF	GO:0009678	pyrophosphate hydrolysis-dr	1/32	10/5513	0.056597	0.345131	0.328845	ATP6V1A	1

MF	GO:0030544	Hsp70 protein binding	1/32	10/5513	0.056597	0.345131	0.328845	HIKESHI	1
MF	GO:0015267	channel activity	3/32	152/5513	0.056906	0.345131	0.328845	PANX1/ATP6V1A/SLC12A2	3
MF	GO:0022803	passive transmembrane trans	3/32	152/5513	0.056906	0.345131	0.328845	PANX1/ATP6V1A/SLC12A2	3
MF	GO:0022857	transmembrane transporter a	5/32	372/5513	0.060854	0.345131	0.328845	PANX1/SLC35F1/ATP6V1C1/ATP6V1A/SL	5
MF	GO:0030246	carbohydrate binding	2/32	73/5513	0.066472	0.345131	0.328845	LGALS7/GANAB	2
MF	GO:0140142	nucleocytoplasmic carrier a	1/32	12/5513	0.067538	0.345131	0.328845	HIKESHI	1
MF	GO:0015075	ion transmembrane transport	4/32	272/5513	0.070442	0.345131	0.328845	PANX1/ATP6V1C1/ATP6V1A/SLC12A2	4
MF	GO:0005484	SNAP receptor activity	1/32	13/5513	0.072962	0.345131	0.328845	VTI1A	1
MF	GO:0016504	peptidase activator activit	1/32	13/5513	0.072962	0.345131	0.328845	APH1B	1
MF	GO:0015020	glucuronosyltransferase act	1/32	14/5513	0.078356	0.345131	0.328845	B3GAT3	1
MF	GO:0016278	lysine N-methyltransferase	1/32	15/5513	0.083719	0.345131	0.328845	CSKMT	1
MF	GO:0016279	protein-lysine N-methyltran	1/32	15/5513	0.083719	0.345131	0.328845	CSKMT	1
MF	GO:0008168	methyltransferase activity	2/32	85/5513	0.086543	0.345131	0.328845	METTL15/CSKMT	2
MF	GO:0017022	myosin binding	1/32	16/5513	0.089052	0.345131	0.328845	RAB6A	1
MF	GO:0019905	syntaxin binding	1/32	16/5513	0.089052	0.345131	0.328845	STXBP3	1
MF	GO:0016741	transferase activity, trans	2/32	89/5513	0.0936	0.345131	0.328845	METTL15/CSKMT	2
MF	GO:0051087	chaperone binding	1/32	17/5513	0.094355	0.345131	0.328845	SLC12A2	1
MF	GO:0015294	solute:cation symporter act	1/32	18/5513	0.099628	0.345131	0.328845	SLC12A2	1
MF	GO:0001221	transcription coregulator b	1/32	19/5513	0.104871	0.345131	0.328845	FOXP1	1
MF	GO:0004812	aminoacyl-tRNA ligase activ	1/32	20/5513	0.110085	0.345131	0.328845	LARS2	1
MF	GO:0016875	ligase activity, forming ca	1/32	20/5513	0.110085	0.345131	0.328845	LARS2	1
MF	GO:0019003	GDP binding	1/32	20/5513	0.110085	0.345131	0.328845	RAP1B	1
MF	GO:0019829	ATPase-coupled cation trans	1/32	20/5513	0.110085	0.345131	0.328845	ATP6V1A	1
MF	GO:0005262	calcium channel activity	1/32	21/5513	0.115269	0.351874	0.33527	PANX1	1
MF	GO:0005319	lipid transporter activity	1/32	23/5513	0.12555	0.357795	0.340912	PITPNC1	1
MF	GO:0008047	enzyme activator activity	2/32	107/5513	0.127238	0.357795	0.340912	APH1B/CLPS	2
MF	GO:0022804	active transmembrane transp	2/32	109/5513	0.13114	0.357795	0.340912	ATP6V1A/SLC12A2	2
MF	GO:0016757	glycosyltransferase activit	2/32	111/5513	0.135071	0.357795	0.340912	ST6GAL1/B3GAT3	2
MF	GO:0000149	SNARE binding	1/32	25/5513	0.135715	0.357795	0.340912	STXBP3	1
MF	GO:0008276	protein methyltransferase a	1/32	25/5513	0.135715	0.357795	0.340912	CSKMT	1
MF	GO:0046873	metal ion transmembrane tra	2/32	114/5513	0.141018	0.359763	0.342787	PANX1/SLC12A2	2
MF	GO:0008170	N-methyltransferase activit	1/32	27/5513	0.145766	0.359763	0.342787	CSKMT	1
MF	GO:0015293	symporter activity	1/32	27/5513	0.145766	0.359763	0.342787	SLC12A2	1
MF	GO:0015085	calcium ion transmembrane t	1/32	30/5513	0.16063	0.386578	0.368336	PANX1	1
MF	GO:0004674	protein serine/threonine ki	2/32	125/5513	0.163296	0.386578	0.368336	CDC42BPA/CDK19	2

MF	GO:0004553	hydrolase activity, hydroly	1/32	34/5513	0.180059	0.40731	0.38809	GANAB	1
MF	GO:0140104	molecular carrier activity	1/32	34/5513	0.180059	0.40731	0.38809	HIKESHI	1
MF	GO:0001217	DNA-binding transcription r	1/32	36/5513	0.18961	0.40731	0.38809	FOXP1	1
MF	GO:0001227	DNA-binding transcription r	1/32	36/5513	0.18961	0.40731	0.38809	FOXP1	1
MF	GO:0015108	chloride transmembrane tran	1/32	36/5513	0.18961	0.40731	0.38809	SLC12A2	1
MF	GO:0042626	ATPase-coupled transmembran	1/32	37/5513	0.194345	0.409891	0.390549	ATP6V1A	1
MF	GO:0140101	catalytic activity, acting	1/32	41/5513	0.213017	0.439485	0.418747	LARS2	1
MF	GO:0003924	GTPase activity	2/32	150/5513	0.215954	0.439485	0.418747	RAB6A/RAP1B	2
MF	GO:0008757	S-adenosylmethionine-depend	1/32	44/5513	0.226746	0.453492	0.432093	CSKMT	1
MF	GO:0016301	kinase activity	3/32	297/5513	0.246711	0.481618	0.458892	CDC42BPA/MOB3B/CDK19	3
MF	GO:0008194	UDP-glycosyltransferase act	1/32	49/5513	0.249113	0.481618	0.458892	B3GAT3	1
MF	GO:0015103	inorganic anion transmembra	1/32	51/5513	0.257883	0.490401	0.46726	SLC12A2	1
MF	GO:0015291	secondary active transmembr	1/32	52/5513	0.262231	0.490626	0.467475	SLC12A2	1
MF	GO:0015399	primary active transmembran	1/32	57/5513	0.283603	0.49074	0.467584	ATP6V1A	1
MF	GO:0016798	hydrolase activity, acting	1/32	57/5513	0.283603	0.49074	0.467584	GANAB	1
MF	GO:0005525	GTP binding	2/32	182/5513	0.285326	0.49074	0.467584	RAB6A/RAP1B	2
MF	GO:0004672	protein kinase activity	2/32	183/5513	0.287502	0.49074	0.467584	CDC42BPA/CDK19	2
MF	GO:0052689	carboxylic ester hydrolase	1/32	58/5513	0.287804	0.49074	0.467584	LARS2	1
MF	GO:0032561	guanyl ribonucleotide bindi	2/32	185/5513	0.291852	0.49074	0.467584	RAB6A/RAP1B	2
MF	GO:0019001	guanyl nucleotide binding	2/32	186/5513	0.294027	0.49074	0.467584	RAB6A/RAP1B	2
MF	GO:0106310	protein serine kinase activ	1/32	60/5513	0.296136	0.49074	0.467584	CDC42BPA	1
MF	GO:0005524	ATP binding	4/32	489/5513	0.314559	0.513927	0.489676	CDC42BPA/LARS2/CDK19/ATP6V1A	4
MF	GO:0008017	microtubule binding	1/32	66/5513	0.32057	0.516473	0.492102	CEP350	1
MF	GO:0032559	adenyl ribonucleotide bindi	4/32	500/5513	0.329507	0.519407	0.494898	CDC42BPA/LARS2/CDK19/ATP6V1A	4
MF	GO:0061135	endopeptidase regulator act	1/32	69/5513	0.332476	0.519407	0.494898	APH1B	1
MF	GO:0016772	transferase activity, trans	3/32	355/5513	0.340171	0.519407	0.494898	CDC42BPA/MOB3B/CDK19	3
MF	GO:0016758	hexosyltransferase activity	1/32	71/5513	0.340301	0.519407	0.494898	B3GAT3	1
MF	GO:0061134	peptidase regulator activit	1/32	77/5513	0.363247	0.547229	0.521407	APH1B	1
MF	GO:0008092	cytoskeletal protein bindin	2/32	228/5513	0.384135	0.55913	0.532747	RAB6A/CEP350	2
MF	GO:0004712	protein serine/threonine/ty	1/32	84/5513	0.389041	0.55913	0.532747	CDC42BPA	1
MF	GO:0016746	acyltransferase activity	1/32	84/5513	0.389041	0.55913	0.532747	F13A1	1
MF	GO:0016773	phosphotransferase activity	2/32	231/5513	0.390427	0.55913	0.532747	CDC42BPA/CDK19	2
MF	GO:0008509	anion transmembrane transpo	1/32	88/5513	0.403322	0.570554	0.543631	SLC12A2	1
MF	GO:0015631	tubulin binding	1/32	95/5513	0.427541	0.597527	0.569331	CEP350	1
MF	GO:0017111	ribonucleoside triphosphate	2/32	256/5513	0.441777	0.610073	0.581286	RAB6A/RAP1B	2

MF	GO:0000978	RNA polymerase II cis-regul	1/32	103/5513	0.454054	0.614082	0.585106	FOXP1	1
MF	GO:0019901	protein kinase binding	1/32	106/5513	0.463686	0.614082	0.585106	SLC12A2	1
MF	GO:0003682	chromatin binding	1/32	107/5513	0.46686	0.614082	0.585106	FOXP1	1
MF	GO:0008134	transcription factor bindin	1/32	107/5513	0.46686	0.614082	0.585106	FOXP1	1
MF	GO:0000987	cis-regulatory region seque	1/32	110/5513	0.476273	0.614082	0.585106	FOXP1	1
MF	GO:0016462	pyrophosphatase activity	2/32	276/5513	0.481247	0.614082	0.585106	RAB6A/RAP1B	2
MF	GO:0016817	hydrolase activity, acting	2/32	279/5513	0.487031	0.614082	0.585106	RAB6A/RAP1B	2
MF	GO:0016818	hydrolase activity, acting	2/32	279/5513	0.487031	0.614082	0.585106	RAB6A/RAP1B	2
MF	GO:0019904	protein domain specific bin	1/32	116/5513	0.494619	0.616945	0.587833	RAB6A	1
MF	GO:0019900	kinase binding	1/32	119/5513	0.503557	0.621411	0.592089	SLC12A2	1
MF	GO:0030234	enzyme regulator activity	2/32	300/5513	0.526446	0.642818	0.612485	APH1B/CLPS	2
MF	GO:0000977	RNA polymerase II transcrip	1/32	134/5513	0.545999	0.656178	0.625215	FOXP1	1
MF	GO:0140098	catalytic activity, acting	1/32	135/5513	0.5487	0.656178	0.625215	LARS2	1
MF	GO:0000976	transcription cis-regulator	1/32	161/5513	0.613705	0.71724	0.683396	FOXP1	1
MF	GO:0001067	transcription regulatory re	1/32	162/5513	0.616014	0.71724	0.683396	FOXP1	1
MF	GO:0000981	DNA-binding transcription f	1/32	163/5513	0.618311	0.71724	0.683396	FOXP1	1
MF	GO:0042803	protein homodimerization ac	1/32	166/5513	0.62512	0.717959	0.684081	ST6GAL1	1
MF	GO:0005102	signaling receptor binding	2/32	368/5513	0.640268	0.728148	0.693788	PANX1/SEMA4D	2
MF	GO:0140657	ATP-dependent activity	1/32	186/5513	0.6676	0.75186	0.716382	ATP6V1A	1
MF	GO:0005509	calcium ion binding	1/32	194/5513	0.683252	0.752938	0.717409	FBLN5	1
MF	GO:0140640	catalytic activity, acting	1/32	195/5513	0.685158	0.752938	0.717409	LARS2	1
MF	GO:1990837	sequence-specific double-st	1/32	199/5513	0.69267	0.752938	0.717409	FOXP1	1
MF	GO:0044877	protein-containing complex	1/32	200/5513	0.694521	0.752938	0.717409	RAP1B	1
MF	GO:0003690	double-stranded DNA binding	1/32	220/5513	0.729346	0.772569	0.736114	FOXP1	1
MF	GO:0042802	identical protein binding	2/32	434/5513	0.730037	0.772569	0.736114	FOXP1/ST6GAL1	2
MF	GO:0003700	DNA-binding transcription f	1/32	222/5513	0.732609	0.772569	0.736114	FOXP1	1
MF	GO:0016788	hydrolase activity, acting	1/32	247/5513	0.770315	0.805014	0.767028	LARS2	1
MF	GO:0043565	sequence-specific DNA bindi	1/32	254/5513	0.779915	0.807769	0.769653	FOXP1	1
MF	GO:0046983	protein dimerization activi	1/32	303/5513	0.837029	0.851714	0.811524	ST6GAL1	1
MF	GO:0140110	transcription regulator act	1/32	303/5513	0.837029	0.851714	0.811524	FOXP1	1
MF	GO:0003723	RNA binding	1/32	353/5513	0.880411	0.888067	0.846162	DNAJC17	1
MF	GO:0019899	enzyme binding	1/32	370/5513	0.89243	0.89243	0.850319	SLC12A2	1

KEGG

ID	Description	GeneRatic	BgRatio	pvalue	p.adjust	qvalue	geneID	Count

oas04966	Collecting duct acid secret	2/42	28/9674	0.006476	0.44612	0.428275	ATP6V1C1/ATP6V1A	2
oas04520	Adherens junction	3/42	100/9674	0.009189	0.44612	0.428275	ACTN4/RAP1B/ROCK2	3
oas04972	Pancreatic secretion	3/42	106/9674	0.010769	0.44612	0.428275	RAP1B/PLCB1/SLC12A2	3
oas04670	Leukocyte transendothelial	3/42	122/9674	0.015712	0.44612	0.428275	ACTN4/RAP1B/ROCK2	3
oas04611	Platelet activation	3/42	128/9674	0.017845	0.44612	0.428275	RAP1B/PLCB1/ROCK2	3
oas00510	N-Glycan biosynthesis	2/42	57/9674	0.02526	0.487293	0.467801	ST6GAL1/GANAB	2
oas04720	Long-term potentiation	2/42	69/9674	0.035941	0.487293	0.467801	RAP1B/PLCB1	2
oas00562	Inositol phosphate metaboli	2/42	74/9674	0.040823	0.487293	0.467801	PLCB1/MTMR6	2
oas05211	Renal cell carcinoma	2/42	77/9674	0.043866	0.487293	0.467801	TGFA/RAP1B	2
oas04360	Axon guidance	3/42	187/9674	0.047062	0.487293	0.467801	ROCK2/EFNA1/SEMA4D	3
oas04721	Synaptic vesicle cycle	2/42	82/9674	0.04912	0.487293	0.467801	ATP6V1C1/ATP6V1A	2
oas04062	Chemokine signaling pathway	3/42	196/9674	0.052809	0.487293	0.467801	RAP1B/PLCB1/ROCK2	3
oas04510	Focal adhesion	3/42	207/9674	0.060275	0.487293	0.467801	ACTN4/RAP1B/ROCK2	3
oas04970	Salivary secretion	2/42	96/9674	0.06495	0.487293	0.467801	PLCB1/SLC12A2	2
oas05323	Rheumatoid arthritis	2/42	98/9674	0.067336	0.487293	0.467801	ATP6V1C1/ATP6V1A	2
oas04015	Rap1 signaling pathway	3/42	218/9674	0.068214	0.487293	0.467801	RAP1B/PLCB1/EFNA1	3
oas04070	Phosphatidylinositol signal	2/42	99/9674	0.06854	0.487293	0.467801	PLCB1/MTMR6	2
oas04933	AGE-RAGE signaling pathway	2/42	103/9674	0.073427	0.487293	0.467801	NOX4/PLCB1	2
oas04980	Cobalamin transport and met	1/42	18/9674	0.075393	0.487293	0.467801	MTRR	1
oas05146	Amoebiasis	2/42	113/9674	0.086116	0.487293	0.467801	ACTN4/PLCB1	2
oas00532	Glycosaminoglycan biosynthe	1/42	21/9674	0.087407	0.487293	0.467801	B3GAT3	1
oas04014	Ras signaling pathway	3/42	245/9674	0.089601	0.487293	0.467801	TGFA/RAP1B/EFNA1	3
oas00534	Glycosaminoglycan biosynthe	1/42	24/9674	0.099269	0.487293	0.467801	B3GAT3	1
oas05417	Lipid and atherosclerosis	3/42	259/9674	0.101685	0.487293	0.467801	RAP1B/PLCB1/ROCK2	3
oas04071	Sphingolipid signaling path	2/42	127/9674	0.104891	0.487293	0.467801	PLCB1/ROCK2	2
oas03013	Nucleocytoplasmic transport	2/42	134/9674	0.114667	0.487293	0.467801	NUP107/NUP58	2
oas05014	Amyotrophic lateral scleros	4/42	441/9674	0.122941	0.487293	0.467801	SIGMAR1/NUP107/PSMD12/NUP58	4
oas04270	Vascular smooth muscle cont	2/42	142/9674	0.126117	0.487293	0.467801	PLCB1/ROCK2	2
oas04915	Estrogen signaling pathway	2/42	143/9674	0.127567	0.487293	0.467801	TGFA/PLCB1	2
oas05010	Alzheimer disease	4/42	450/9674	0.129727	0.487293	0.467801	APH1B/NOX4/PLCB1/PSMD12	4
oas04010	MAPK signaling pathway	3/42	306/9674	0.146574	0.487293	0.467801	TGFA/RAP1B/EFNA1	3
oas04921	Oxytocin signaling pathway	2/42	157/9674	0.148276	0.487293	0.467801	PLCB1/ROCK2	2
oas04130	SNARE interactions in vesic	1/42	37/9674	0.148953	0.487293	0.467801	VTI1A	1
oas04934	Cushing syndrome	2/42	158/9674	0.149782	0.487293	0.467801	RAP1B/PLCB1	2
oas03030	DNA replication	1/42	38/9674	0.152662	0.487293	0.467801	RNASEH2B	1

oas05017	Spinocerebellar ataxia	2/42	162/9674	0.155836	0.487293	0.467801	PLCB1/PSMD12	2
oas05143	African trypanosomiasis	1/42	39/9674	0.156355	0.487293	0.467801	PLCB1	1
oas04148	Efferocytosis	2/42	165/9674	0.160408	0.487293	0.467801	PANX1/LOC101115632	2
oas04145	Phagosome	2/42	169/9674	0.166545	0.487293	0.467801	ATP6V1C1/ATP6V1A	2
oas04150	mTOR signaling pathway	2/42	170/9674	0.168085	0.487293	0.467801	ATP6V1C1/ATP6V1A	2
oas04022	cGMP-PKG signaling pathway	2/42	173/9674	0.172723	0.487293	0.467801	PLCB1/ROCK2	2
oas04110	Cell cycle	2/42	175/9674	0.175827	0.487293	0.467801	KNL1/E2F5	2
oas04973	Carbohydrate digestion and	1/42	47/9674	0.185341	0.487293	0.467801	PLCB1	1
oas04141	Protein processing in endop	2/42	183/9674	0.188334	0.487293	0.467801	GANAB/UBXN1	2
oas00514	Other types of O-glycan bio	1/42	48/9674	0.188895	0.487293	0.467801	ST6GAL1	1
oas00970	Aminoacyl-tRNA biosynthesis	1/42	48/9674	0.188895	0.487293	0.467801	LARS2	1
oas03050	Proteasome	1/42	49/9674	0.192434	0.487293	0.467801	PSMD12	1
oas04310	Wnt signaling pathway	2/42	186/9674	0.193057	0.487293	0.467801	PLCB1/ROCK2	2
oas04530	Tight junction	2/42	186/9674	0.193057	0.487293	0.467801	ACTN4/ROCK2	2
oas03022	Basal transcription factors	1/42	50/9674	0.195958	0.487293	0.467801	GTF2E1	1
oas04621	NOD-like receptor signaling	2/42	193/9674	0.204142	0.487293	0.467801	PANX1/PLCB1	2
oas04975	Fat digestion and absorptio	1/42	53/9674	0.20644	0.487293	0.467801	CLPS	1
oas00190	Oxidative phosphorylation	2/42	196/9674	0.208916	0.487293	0.467801	ATP6V1C1/ATP6V1A	2
oas04814	Motor proteins	2/42	197/9674	0.210511	0.487293	0.467801	KIF6/DYNLRB2	2
oas04961	Endocrine and other factor-	1/42	57/9674	0.220208	0.500473	0.480454	PLCB1	1
oas04330	Notch signaling pathway	1/42	63/9674	0.240425	0.516408	0.495751	APH1B	1
oas05206	MicroRNAs in cancer	2/42	216/9674	0.241019	0.516408	0.495751	FOXP1/EFNA1	2
oas04730	Long-term depression	1/42	64/9674	0.243744	0.516408	0.495751	PLCB1	1
oas04929	GnRH secretion	1/42	64/9674	0.243744	0.516408	0.495751	PLCB1	1
oas04927	Cortisol synthesis and secr	1/42	67/9674	0.253618	0.525964	0.504926	PLCB1	1
oas05203	Viral carcinogenesis	2/42	233/9674	0.268524	0.525964	0.504926	ACTN4/GTF2E1	2
oas05223	Non-small cell lung cancer	1/42	73/9674	0.272988	0.525964	0.504926	TGFA	1
oas04024	cAMP signaling pathway	2/42	237/9674	0.275005	0.525964	0.504926	RAP1B/ROCK2	2
oas04924	Renin secretion	1/42	74/9674	0.276168	0.525964	0.504926	PLCB1	1
oas04810	Regulation of actin cytoske	2/42	239/9674	0.278246	0.525964	0.504926	ACTN4/ROCK2	2
oas04918	Thyroid hormone synthesis	1/42	78/9674	0.288755	0.525964	0.504926	PLCB1	1
oas05214	Glioma	1/42	78/9674	0.288755	0.525964	0.504926	TGFA	1
oas04971	Gastric acid secretion	1/42	79/9674	0.291868	0.525964	0.504926	PLCB1	1
oas05212	Pancreatic cancer	1/42	79/9674	0.291868	0.525964	0.504926	TGFA	1
oas04742	Taste transduction	1/42	82/9674	0.301127	0.525964	0.504926	PLCB1	1

oas04020	Calcium signaling pathway	2/42	257/9674	0.307368	0.525964	0.504926	TGFA/PLCB1	2
oas04012	ErbB signaling pathway	1/42	85/9674	0.310268	0.525964	0.504926	TGFA	1
oas04911	Insulin secretion	1/42	88/9674	0.319293	0.525964	0.504926	PLCB1	1
oas01521	EGFR tyrosine kinase inhibi	1/42	89/9674	0.322275	0.525964	0.504926	TGFA	1
oas03083	Polycomb repressive complex	1/42	90/9674	0.325245	0.525964	0.504926	EED	1
oas04540	Gap junction	1/42	90/9674	0.325245	0.525964	0.504926	PLCB1	1
oas05163	Human cytomegalovirus infec	2/42	269/9674	0.326688	0.525964	0.504926	PLCB1/ROCK2	2
oas04610	Complement and coagulation	1/42	91/9674	0.328202	0.525964	0.504926	F13A1	1
oas04912	GnRH signaling pathway	1/42	94/9674	0.336997	0.532968	0.511649	PLCB1	1
oas05132	Salmonella infection	2/42	278/9674	0.341099	0.532968	0.511649	ROCK2/DYNLRB2	2
oas05215	Prostate cancer	1/42	99/9674	0.351407	0.537778	0.516267	TGFA	1
oas04713	Circadian entrainment	1/42	101/9674	0.357084	0.537778	0.516267	PLCB1	1
oas04925	Aldosterone synthesis and s	1/42	101/9674	0.357084	0.537778	0.516267	PLCB1	1
oas04916	Melanogenesis	1/42	104/9674	0.36551	0.538799	0.517247	PLCB1	1
oas03008	Ribosome biogenesis in euka	1/42	106/9674	0.371067	0.538799	0.517247	RPP25L	1
oas05210	Colorectal cancer	1/42	107/9674	0.373828	0.538799	0.517247	TGFA	1
oas04750	Inflammatory mediator regul	1/42	109/9674	0.379314	0.538799	0.517247	PLCB1	1
oas04922	Glucagon signaling pathway	1/42	109/9674	0.379314	0.538799	0.517247	PLCB1	1
oas04725	Cholinergic synapse	1/42	113/9674	0.390146	0.545591	0.523767	PLCB1	1
oas04724	Glutamatergic synapse	1/42	114/9674	0.392825	0.545591	0.523767	PLCB1	1
oas04350	TGF-beta signaling pathway	1/42	119/9674	0.406049	0.547927	0.52601	E2F5	1
oas04928	Parathyroid hormone synthes	1/42	119/9674	0.406049	0.547927	0.52601	PLCB1	1
oas04726	Serotonergic synapse	1/42	121/9674	0.411259	0.547927	0.52601	PLCB1	1
oas04935	Growth hormone synthesis, s	1/42	122/9674	0.413848	0.547927	0.52601	PLCB1	1
oas05142	Chagas disease	1/42	123/9674	0.416425	0.547927	0.52601	PLCB1	1
oas04919	Thyroid hormone signaling p	1/42	125/9674	0.421546	0.548888	0.526933	PLCB1	1
oas04722	Neurotrophin signaling path	1/42	127/9674	0.426624	0.549773	0.527782	RAP1B	1
oas04926	Relaxin signaling pathway	1/42	132/9674	0.439128	0.557572	0.535269	PLCB1	1
oas04974	Protein digestion and absor	1/42	133/9674	0.441597	0.557572	0.535269	LOC101122563	1
oas04728	Dopaminergic synapse	1/42	136/9674	0.44894	0.561175	0.538728	PLCB1	1
oas04380	Osteoclast differentiation	1/42	139/9674	0.456188	0.564589	0.542006	CALCR	1
oas05165	Human papillomavirus infect	2/42	359/9674	0.465438	0.57039	0.547574	ATP6V1C1/ATP6V1A	2
oas05016	Huntington disease	2/42	363/9674	0.471252	0.57083	0.547997	PLCB1/PSMD12	2
oas04371	Apelin signaling pathway	1/42	147/9674	0.475065	0.57083	0.547997	PLCB1	1
oas05322	Systemic lupus erythematosu	1/42	151/9674	0.484262	0.57083	0.547997	ACTN4	1

oas04072	Phospholipase D signaling p	1/42	153/9674	0.488802	0.57083	0.547997	PLCB1	1
oas05135	Yersinia infection	1/42	153/9674	0.488802	0.57083	0.547997	ROCK2	1
oas04723	Retrograde endocannabinoid	1/42	157/9674	0.497764	0.57083	0.547997	PLCB1	1
oas04936	Alcoholic liver disease	1/42	157/9674	0.497764	0.57083	0.547997	NOX4	1
oas04261	Adrenergic signaling in car	1/42	161/9674	0.506573	0.575651	0.552625	PLCB1	1
oas04151	PI3K-Akt signaling pathway	2/42	396/9674	0.517806	0.583115	0.55979	TGFA/EFNA1	2
oas04218	Cellular senescence	1/42	169/9674	0.52374	0.584531	0.56115	E2F5	1
oas05225	Hepatocellular carcinoma	1/42	183/9674	0.55239	0.611051	0.586609	TGFA	1
oas04613	Neutrophil extracellular tr	1/42	208/9674	0.599423	0.657262	0.630972	PLCB1	1
oas05205	Proteoglycans in cancer	1/42	219/9674	0.618556	0.672343	0.64545	ROCK2	1
oas04820	Cytoskeleton in muscle cell	1/42	236/9674	0.646382	0.696533	0.668671	FMNL2	1
oas05169	Epstein-Barr virus infectio	1/42	241/9674	0.654182	0.698913	0.670956	PSMD12	1
oas05415	Diabetic cardiomyopathy	1/42	248/9674	0.664821	0.704259	0.676089	PLCB1	1
oas04144	Endocytosis	1/42	261/9674	0.683737	0.718211	0.689483	VPS37C	1
oas05208	Chemical carcinogenesis - r	1/42	278/9674	0.706908	0.736363	0.706908	NOX4	1
oas05020	Prion disease	1/42	329/9674	0.766917	0.788981	0.757422	PSMD12	1
oas05012	Parkinson disease	1/42	332/9674	0.770046	0.788981	0.757422	PSMD12	1
oas04080	Neuroactive ligand-receptor	1/42	383/9674	0.817371	0.830662	0.797435	CALCR	1
oas05168	Herpes simplex virus 1 infe	1/42	433/9674	0.854479	0.86137	0.826915	SRPK1	1
oas05171	Coronavirus disease - COVID	1/42	472/9674	0.878211	0.878211	0.843082	F13A1	1
