# Supplementary material for: Genetic Structure and Selection Signals for Extreme Environment Adaptation in Lop Sheep of Xinjiang
Source: Biology (Basel). 2025 Mar 25;14(4):337. doi: 10.3390/biology14040337 (PMC12025199; doi:10.3390/biology14040337)
Supplement: Supplementary file 1 [file biology-14-00337-s001.zip › Supplementary Table S7.pdf]

Supplementary Table S7

Collection region	Number	Altitude（m）	annual rainfall（mm）	latitude	
Yuli County	30	800 ~ 2782	43	E: 84°02′∽89°58′	N: 40°10′∽41°39′
Ruoqiang County	80	768 ~ 6900	28.5	E: 86°45′ ~ 93°45′	N: 36°05′ ~ 41°23′
