# Supplementary figures and images for: Genetic Structure and Selection Signals for Extreme Environment Adaptation in Lop Sheep of Xinjiang
Source: Biology (Basel). 2025 Mar 25;14(4):337. doi: 10.3390/biology14040337 (PMC12025199; doi:10.3390/biology14040337)

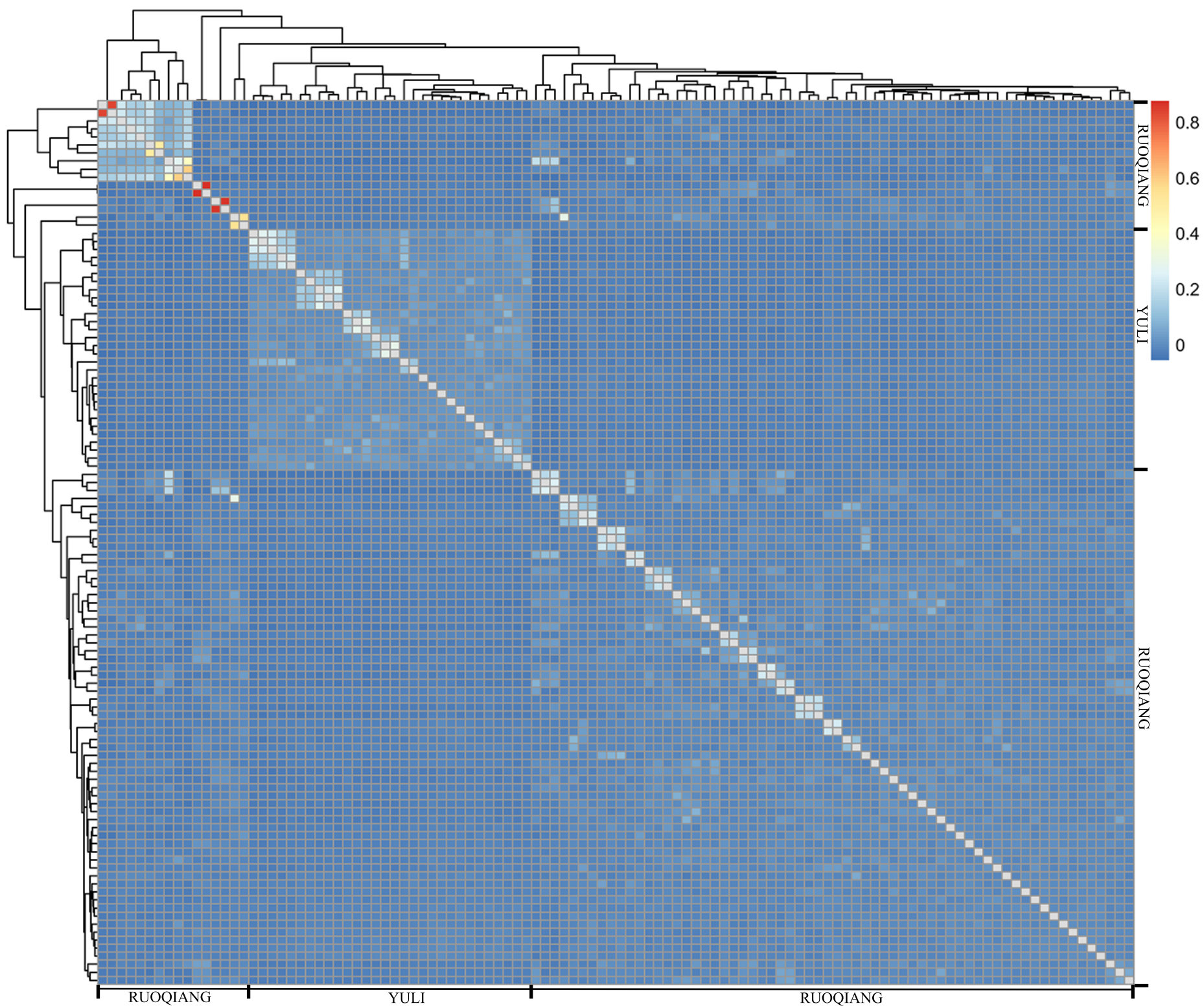

Supplement: Supplementary file 1 [file biology-14-00337-s001.zip › Supplementary Figure S1.pdf]
